# Supplementary material for: Effects of minimum unit pricing for alcohol in South Africa across different drinker groups and wealth quintiles: a modelling study
Source: BMJ Open. 2021 Aug 9;11(8):e052879. doi: 10.1136/bmjopen-2021-052879 (PMC8354280; doi:10.1136/bmjopen-2021-052879)
Supplement: Supplementary data [file bmjopen-2021-052879supp001.pdf]

Contents

Supplementary Material 2
Price to consumption 2
1. Estimating baseline consumption using South African Demographic and Health Survey (SADHS) 2
2. Uplifting consumption 5
3. Uplifting peak consumption 8
4. Wealth quintiles 8
5. International Alcohol Control Study 2014 for prices 9
6. Base prices by subgroup 10
7. Adjusting the elasticities 11
8. Individual spend, tax and retail revenue 12
Consumption to harm 13
9. Relative risks 13
10. Potential impact fractions 14
11. Socioeconomic gradients of ill health 14
12. Distributing baseline deaths and cases and calculating probabilities 15
13. Baseline health and lagged health impact 16
14. Hospital multipliers and costs 17
15. Sensitivity Analysis 19
16. Healthcare cost savings by quintile 23
References 24

Supplementary Material

Price to consumption

Our model starts by estimating mean and peak alcohol consumption at current alcohol prices at the individual level. The proportion of alcohol consumption which is homebrew is also estimated. This process utilised both alcohol frequency questions and seven day recall questions asked in the same survey. As survey data significantly underreports consumption we calibrate these estimates to market research data using statistical methods established in the literature [1-3]. Following the shift of mean consumption, peak consumption is re-estimated using a simple regression model created at baseline. We categorise drinkers into three exhaustive and mutually exclusive groups; moderate (less than 15 standard drinks per week); occasional binge (less than 15 drinks per week but more than 5 on one occasion); and heavy (15 or more drinks per week). A standard drink in South Africa is currently 15ml or 12 grams of pure ethanol. We compute a regression model for wealth quintiles using the South African Demographic and Health Survey (SADHS) data and use it to predict wealth quintiles in the International Alcohol Control (IAC) dataset to generate price distributions for wealth and drinker groups. Alcohol is treated as one commodity due to data constraints.

1. Estimating baseline consumption using South African Demographic and Health Survey (SADHS)

The SADHS survey asked the following questions:

Table 1: Survey questions

Survey	Alcohol questions<br>[answers]
**SADHS 2016**	Have you ever consumed a drink that contains alcohol such as beer, wine, ciders, spirits, or sorghum beer?<br>Probe: Even one drink?<br>[yes, no]<br><br>Was this within the last 12 months?<br>[yes, no]<br><br>In the last 12 months, how frequently have you had at least one drink?<br>[5 or more days a week, 1-4 days per week, 1-3 days a month, less often than once a month]<br><br>During each of the last 7 days, how many standard drinks did you have?<br>[use showcard, record total number of drinks consumed each day starting with the day before the day of the interview and proceeding backwards]<br><br>During the last 7 days, how many standard home-made beers or other homemade alcohol did you have?<br>[use showcard, record number]<br><br>In the past 30 days, have you consumed five or more standard drinks on at least one occasion?<br>[yes, no]

Process of adjusting the SADHS estimates

Drinkers were categorised by their drinking frequency and by whether or not they had reported any drinking in the last seven days.

Table 2: Frequency of alcohol consumption responses

Drinking occasion frequency	count	Reported drinks in last 7 days	Reported zero drinks in last 7 days
5 or more days a week	293	266	27 (6 binge, 21 drinker)
1-4 days per week	668	565	103 (29 binge, 74 drinker)
1-3 days per month	1163	799	364 (all drinkers)
less often than once a month	1187	404	783 (all drinkers)
NA	7025		

Readjusting those with a seven day drinking pattern (pink numbers)

The pink numbers are respondents who say they only drink 1 -3 days per month or less often than once a month but have drunk in the last 7 days. If this were multiplied by 52 it would be an overestimate. Therefore, we assumed for those that drink 1-3 days per month we have captured their one drinking week in the month and multiply by 12 to get their annual consumption. There are 799 people in this category. We assumed for those who drink less often than once a month but who did drink in the last week we have caught their one drinking week that occurs every two months. We multiplied by six, to get the annual figure. There are 404 people in this category. The yellow numbers do not require adjustment as respondents report drinking every week and have a seven day drinking pattern.

Readjusting those without a seven day drinking pattern but who say they drink (blue and red numbers)

For those with a drink frequency of five or more days per week we used the mean standard drinks for drinkers who reported the same frequency but who do have a seven day pattern, there are 27 people that this applies to (blue).

For those with a drink frequency of 1 – 4 days per week we used the mean standard drinks for drinkers who report the same frequency but who do have a seven day pattern, there are 103 people in this group (blue).

For those with a drink frequency of 1 – 3 days per month we used the mean adjusted annual drinks (adjusted in 2.2.1.3.1) of the equivalent frequency group who did report a drinking pattern. There are 364 drinkers in this group (red).

All of the above estimates were computed for sex and binge drinking subgroups.

For those with a drink frequency of less than once per month we used the mean adjusted annual drinks (adjusted in 2.2.1.3.1) of the equivalent frequency group who did report a drinking pattern. This is computed for subgroups based on sex and binge drinking. There are 783 people in this group (red)

Process of adjusting peak drinks

Using the same process as above we applied a peak drink to those observations without one. As an additional check we validated that all those reporting binge drinking had a peak drink at minimum of 5.

Comparing the adjusted SADHS data with the estimates using only 7 day recall as expected prevalence of drinking increases and per capita estimates reduce (Table 6). The prevalence estimates are now broadly similar to the NiDs and GISAH estimates (Table 3).

Table 3: Comparing adjusted with unadjusted statistics

	**Prevalence of drinking**		**Sample size: drinkers**	**Annual litres of alcohol - per capita**			**Annual litres of alcohol - Just drinkers**		
	Female	Male		Total	Female	Male	Total	Female	Male
**SADHS (7 day recall only) Population weights applied**	9.3%	32.7%	n = 1949 (report drinks in the 7 day recall) females = 571 males = 1378	2.2	0.65	4.5	10.6	6.54	12.2
**SADHS adjusted (7 day recall plus adjustments based on frequency questions) Population weights applied**	18%	54%	n = 3311 females = 1125 males = 2186	1.65	0.50	3.4	5.0	2.59	6.25

Figure 1: Density plot of female drinkers before and after the shift

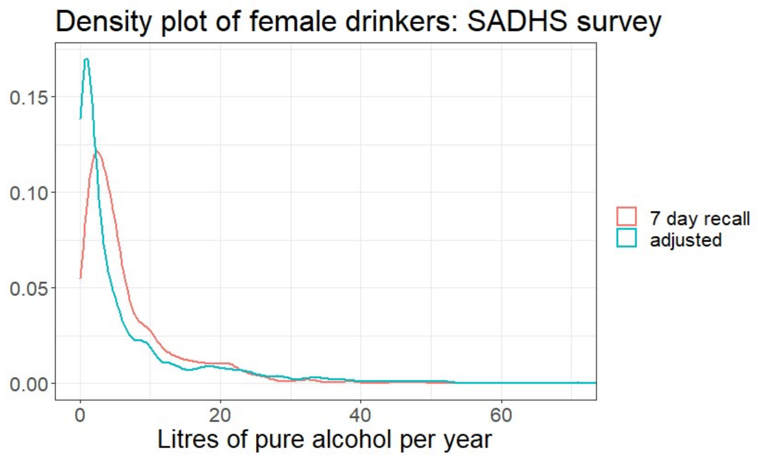

Figure 2: Density plot of male drinkers before and after the shift

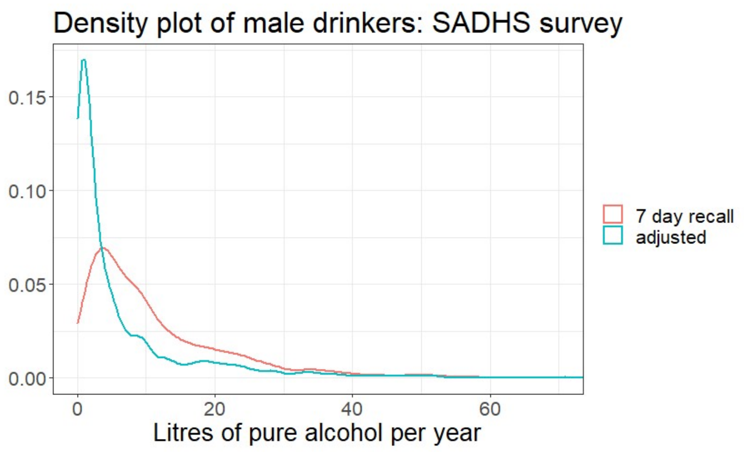

Incorporating the frequency data into the seven day recall moves the distribution towards the left (Figures 1 and 2). This is logical as the sample will now include those drinkers who stated that they drink but did not record any for the last seven days, it also adjusted down those who claim to drink less than weekly but who did recall drinks for the last seven days. This pattern gives some confidence in the dataset and utilises the strengths of capturing heavy drinking well and including occasional drinkers.

2. Uplifting consumption

Surveys provide important data about drinking patterns within the population but total consumption estimates are far smaller than that indicated by administrative sources [4]. As this is a global phenomenon there are established statistical calibration methods in the academic literature. The steps are broadly as follows:

- compute the ratio between survey and sales per capita consumption (known as coverage)
- use this ratio to adjust the mean for each subpopulation of interest
- use the new mean to estimate an associated standard deviation based on a published relationship, estimated using regression on a large global dataset [2]

$$\hat{\sigma}_{shifted} = 1.174 \times \hat{\mu}_{shifted} + 1.003 \times female$$

- use the new mean and standard deviation to generate the shape and rate parameters and fit a gamma distribution

This method relies on three assumptions. Firstly that the sales data accurately reflects per capita consumption. Secondly, that the true proportion of abstainers has been captured by the survey, and finally that under-estimation of consumption is the same across all population groups.

Two additional key limitations have been identified with regards to this method. Firstly, there is no empirical evidence that under-coverage is distributed as implied by the shifts needed to fit the adjusted consumption to the gamma. Secondly, that shifting consumption to a gamma can artificially reduce the long tail of heavy drinkers [3]. To address the second point a proposed method is to fit a gamma distribution to the survey and for each percentile of the distribution calculate the percentage consumption increase and apply these percentage shifts to the corresponding percentile of the survey data.

The following steps outline, in detail, how we calibrated the SADHS dataset to Euromonitor figures:

- First a cap was applied to all drinkers of 68 litres of alcohol per year or 150 grams of alcohol per day. As the model includes long term effects (20 years) the cap is needed as a higher level of alcohol cannot be sustained in the long term [5]. This cap impacted one woman and ten men. Of this small group only two men drunk both homebrew and recorded alcohol and so their total consumption was reduced to 68 litres and then split into recorded and homebrew using their previous percentage split.

- Survey coverage level was calculated as the difference between total per capita consumption recorded in the SADHS survey and per capita consumption using Euromonitor recorded sales data for 2018. 80% of the sales data is used to account for spillage,

stockpiling and tourist consumption. This sales figure was then increased to take account of the 4.15% of total alcohol consumed in the SADHS survey reported as homebrew (representing unrecorded alcohol in the model). The comparison of total consumption according to the survey and the adjusted official sales data was used to calculate a coverage of 27%.

- For female and male subgroups the mean litres of alcohol was adjusted by the multiplication factor. This adjusted mean was used to estimate an associated standard deviation based on a previously established relationship between the two. These were then used to fit a "shifted" gamma distribution (maintaining the cap of 68 litres), calculated for male and females separately.

- A gamma distribution was fitted to the original sample of drinkers, by sex, and percentiles were taken across this and the shifted distribution. Percentage differences in consumption were calculated. These increases were then applied to the percentiles of the original survey sample.

- Each individual's total consumption was split into homebrew and recorded alcohol using the original percentage split (this assumes underreporting is equal across homebrew and recorded alcohol).

- Results were compared visually and via a table (Table 7 and Figures 8 and 9). There is a small difference between the two methods, more visible for males than females. It appears adjusting by percentiles only makes a difference at the extremes, lowering the left hand peak slightly but also falling below the Gamma shifted distribution after 60 litres of alcohol per year for men meaning there is a smaller number of the very high drinkers.

The percentile adjusted distribution was used for the main model base on expert opinion.

Table 4: Comparing pre and post shift data

Females – litres of alcohol per year	Mean	Min	Max
**SADHS Survey data (weighted mean and capped)**	2.57	0.09	68
**Gamma fitted to survey (difference due to weights)**	2.50	0.09	68
**Gamma shift**	10.78	1	68
**Adjusting each percentile (weighted mean)**	10.74	0.5	68
Males – litres of alcohol per year			
**SADHS Survey data (weighted mean and capped)**	6.13	0.09	68
**Gamma fitted to survey (difference due to weights)**	5.6	0.09	68
**Gamma distribution shifted**	18.55	1	68
**Adjusting each percentile (weighted mean)**	19.2	0.5	68

Figure 3: Comparing distributions pre and post shift females

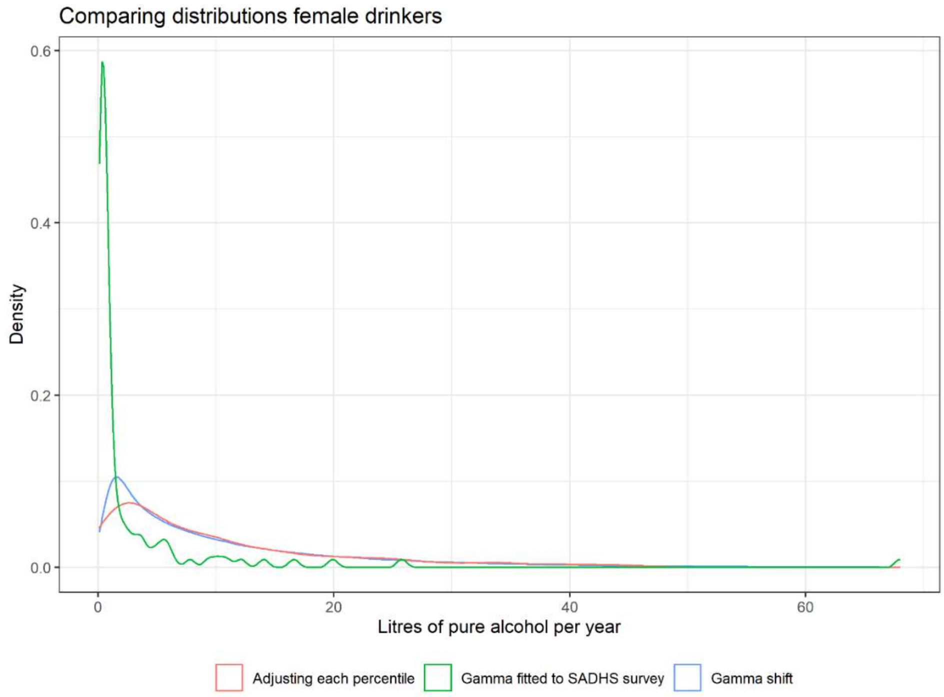

Figure 4: Comparing distributions pre and post shift males

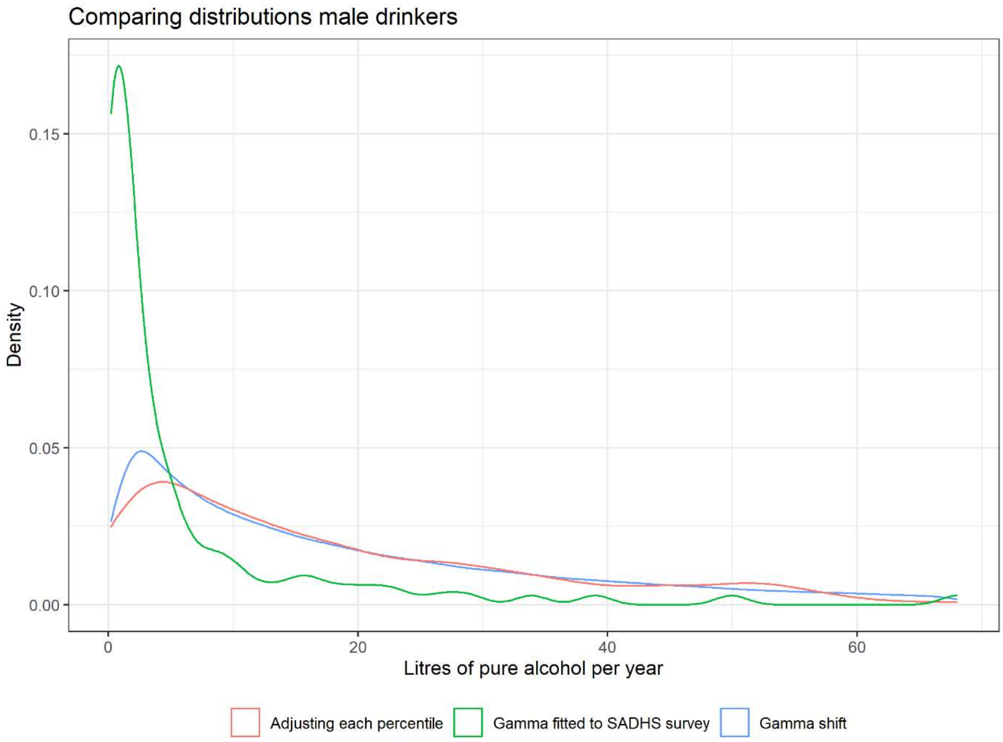

3. Uplifting peak consumption

Peak drinking measures the highest number of drinks consumed on a single drinking occasion and therefore relates to intoxication which is associated with harms such as road injury, interpersonal violence and self-harm. Following the method used in the Sheffield Alcohol Policy Model [6], the following linear regression model was fitted, for all drinkers, to the non-shifted SADHS data, relating peak drinking to mean consumption, age and sex.

$$peak_{\ ij}(SADHS) = \beta_0 + \ \beta_1 \times ageband + \ \beta_2 \ \times sex$$

The model was used to compute fitted values for the non-shifted data. The model assumes there is a linear relationship between peak and mean consumption, the magnitude of which is allowed to vary by age and sex.

After the mean consumption was shifted as above the corresponding new peak consumption was computed using the following formula:

$$peak_{\ ij}(shifted) = \text{peak}_{ij}\ (SADHS) \times \left(\frac{E(peak_{ij}(shifted))}{E(peak_{ij}(SADHS))}\right)$$

The linear relationship between mean and peak estimated from the SADHS survey is maintained for the shifted mean and peak consumption, this assumes individuals under reported peak and mean consumption by the same magnitude. The method also assumes the prediction error for the model is of the same magnitude for all levels of consumption.

The predictions were checked to ensure that peak estimates were not below mean daily drinking. There were 88 people (out of the 3311 drinkers) for whom this was true. These people had their peak drinking increased to match their mean daily drinking.

4. Wealth quintiles

In order to match wealth groups between the two datasets an ordered choice model was created using SADHS data with wealth quintile (1 – 5) as the dependent variable, using the MASS package in R [7]. Wealth groups were chosen as the best available measure to capture socioeconomic status that allowed us to match between the SADHS and IAC dataset. Although income was asked in the IAC dataset many of the respondents refused to answer resulting in a very small sample.

All the variables that were common across the two datasets were included in the initial model, these were not just asset ownership but also age, sex, educational level and population group (race). Stepwise regression was performed using the step.AIC function. This chooses the best variables to include by running the regression with all variables in and then taking one out and computing a goodness of fit measure (the AIC). If the goodness of fit measure is improved then that model is preferred, it runs this for many models until it finds the model with the highest AIC. This method resulted in the selection of the following variables: age, sex, population group, education level, car, landline, electricity, fridge, computer, radio, tv. The only variable it removed was mobile phone which fitted anecdotally with conversations we had with stakeholders in South Africa regarding how much poorer people prioritise mobile phones.

The goodness of fit matrix evaluates the success of the model, comparing the closeness of the predicted and observed outcome (Table 5). The model never predicts the poorest as the richest or the richest as the poorest.

Table 5: Goodness of fit matrix

	Prediction					
Actual		Poorest	Poorer	Middle	Richer	Richest
	Poorest	1300	593	196	9	0
	Poorer	299	975	744	192	17
	Middle	62	612	1042	595	26
	Richer	5	236	763	818	244
	Richest	0	10	108	422	1068

5. International Alcohol Control Study 2014 for prices

The IAC dataset provides prices by drinking location by beverage, by container size and also asks whether the individual binge drinks, demographic data is also collected. The survey asked for the price in Rands by location, for example they ask for the price of a beer paid at a pub for each container size. There are 17 drinking locations (12 on trade and 5 off trade) and 12 drink types. On-trade is where the alcohol is consumed on the premises it is purchased (e.g. hotels, restaurants, pubs), off-trade is where the alcohol is consumed off the premises it was purchased at (e.g. supermarket or bottle store).

Prices were disaggregated by population subgroups rather than by drink type (wine/beer/spirits etc). This was consistent with the South Africa specific price elasticities which were calculated for drinker groups whilst treating alcohol as a single commodity. The IAC respondents were categorised into drinker groups using the definitions above. Each price was weighted by the number of units (e.g. bottles, glasses, cans) sold, the container size of those units and the number of drinking occasions in 6 months (Figure 5). Every price observation was validated using data from the South African Consumer Price Index. Prices were increased to 2018 to account for inflation.

Figure 5: Distribution of off-trade and on-trade prices, standard drink is 15ml or 12grams of pure ethanol.

On-trade is where the alcohol is consumed on the premises it is purchased (e.g. hotels, restaurants, pubs), off-trade is where the alcohol is consumed off the premises it was purchased at (e.g. supermarket or bottle store).

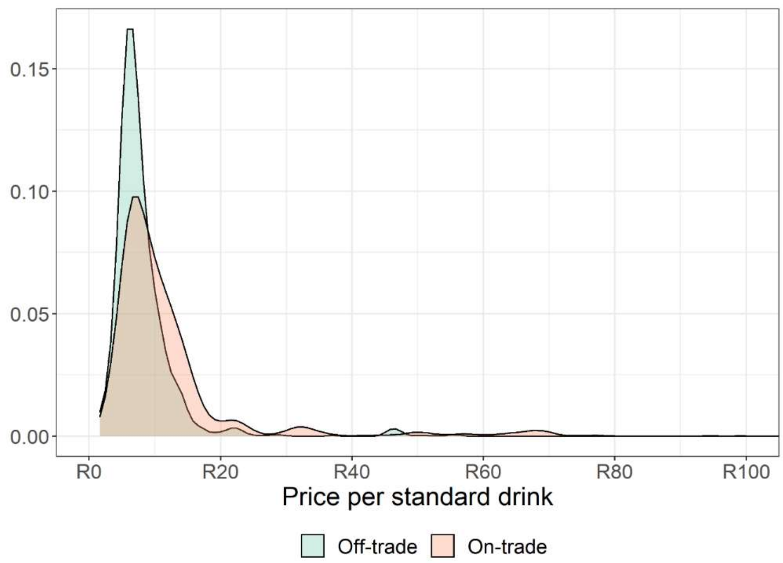

The off-trade wine prices were adjusted using data from the South Africa Wine Industry Statistics [8] who report the proportions of still wine sold (which makes up 93% of total volume of wine sold) in the off-trade in 2018 that falls within different price bands, this data was used to adjust downwards the off-trade wine (Table 6). The price observations were sorted in ascending order and a cumulative volume variable created. The price closest to the 49th percentile was then adjusted down to R3.74 and all prices below adjusted using the same proportion. The prices at the very bottom were adjusted so they could not go below R2.50. The same adjustment process was applied to each of the four groups.

Table 6: Price distribution for off-trade wine

Retail price per litre of wine for 2018	Price per standard drink (15ml) assuming 12% abv	Cumulative percentage of total still wine sold at price SAWIS data	Cumulative percentage of IAC data for off-trade wine pre-adjustment	Cumulative percentage of IAC data for off-trade wine post-adjustment
**Less than R30**	Less than R3.75	49%	33%	51%
**> R30 – R48**	> R3.75 – R6	82%	60%	83%
**> R48 - R72**	> R6 – R9	89%	77%	89%
**> R72 - 108**	> R9 – R13.5	95%	89%	95%
**> R108**	> R13.5	100%	100%	100%

As the Tschwane prices were collected in one locality, they were validated against national data sources. Beer is by far the most popular drink, accounting for over 50% of the alcohol sold so beer prices are critical. We accessed data from the South Africa Consumer Price Index for January 2020 to compare the Gauteng province (where Tshwane is located) with other provinces. Beer, which accounts for over 50% of alcohol sold in South Africa, Guateng is at R13.76 for a 330ml can. The average across the eight prices listed above is R13.66 which is very close to Guateng's price, therefore we assume the same price distributions across the whole of South Africa.

Finally, prices were validated with all stakeholders including individuals resident in townships who could provide anecdotal evidence relating to cheap alcohol available at shebeens.

6. Base prices by subgroup

All IAC drinkers were now categorised by drinker type and by wealth quintile (Table 7). Wealth quintile was predicted using the ordered choice model created using the SADHS data. Drinkers in the lowest wealth quintile appear the least likely to drink in moderation leaving a very small sample size (this is not weighted by number of drinks). It is therefore not possible to create price distributions for all 15 categories.

Table 7: Count of IAC price observations and respondents within each category

	Moderate obs (individuals)	Occasional Binge obs (individuals)	Heavy obs (individuals)
**Poorest**	2 (2)	29 (23)	35 (24)
**Poorer**	8 (8)	23 (18)	28 (20)
**Middle**	11 (11)	132 (90)	88 (40)
**Richer**	23 (20)	95 (59)	60 (30)
**Richest**	93 (68)	135 (93)	101 (50)

The mean price for each of these drinker categories demonstrates there is wealth gradient (Table 8).

Table 8 Mean price of standard alcoholic drink (15ml of pure alcohol) for each subgroup

	Moderate	Occasional Binge	Heavy
**Poorest**	R6.79	R7.97	R7.78
**Poorer**	R9.43	R10.0	R9.65
**Middle**	R10.2	R10.1	R9.23
**Richer**	R11.3	R13.4	R10.6
**Richest**	R11.7	R11.1	R12.8

In order to ensure adequate sample size the poorest/poorer/middle and richer/richest categories were aggregated for moderate drinkers (Table 9). This represents the final group of prices used in the model.

Table 9 Mean price of standard alcoholic drink (15ml of pure alcohol) within each subgroup

	Moderate	Occasional Binge	Heavy
**Poorest**	R9.13	R7.97	R7.78
**Poorer**	R9.13	R10.0	R9.65
**Middle**	R9.13	R10.1	R9.23
**Richer**	R11.6	R13.4	R10.6
**Richest**	R11.6	R11.1	R12.8

7. Adjusting the elasticities

The starting point for elasticities -0.4, -0.22 and -0.18 for moderate, occasional binge and heavy drinkers respectively [9]. We adjusted these elasticities to incorporate an income gradient using -0.86 and -0.5 elasticity for low and high socioeconomic status [10]. To remain on the conservative side we will count the bottom two quintiles as low SES and the top three as high.

Table 10: Elasticities by wealth and drinker group

Drinker type	Q1	Q2	Q3	Q4	Q5
Moderate	-0.53	-0.53	-0.31	-0.31	-0.31
Occasional binge	-0.29	-0.29	-0.17	-0.17	-0.17
Heavy drinkers	-0.24	-0.24	-0.14	-0.14	-0.14

8. Individual spend, tax and retail revenue

Alcohol consumption expenditure
The total retail spend at baseline, and each scenario, was computed by adding up all the individual spends multiplied by their population weights. When the SADHS consumption estimates were shifted to calibrate to market research data only 80% of the consumption figure was used to take account of spillage, stockpiling and tourism, but the 20% of alcohol remains in the headline sales revenue. Therefore to make it comparable we estimate the total sales revenue by increasing the modelled alcohol consumption revenue by 1.25 (100/80).

Government revenue, VAT, excise tax and retail revenue
The following steps outline how we computed government and retail revenue:

1. Calculate VAT by assuming 15% of the base retail spend is VAT
2. Import 2018 base excise tax from Treasury Budget Report [11]
3. Calculate total volume consumed of alcohol at all four scenarios (baseline/R5/R10/R15)
4. Calculate the percentage change in volume from baseline for each of the three policies
5. Apply the percentage change in volume to base excise tax (we assume a fixed ratio between volume and excise tax)
6. Calculate retail revenue by: spend - vat - excise tax

It is likely this is a conservative approach to modelling excise tax revenue as generally the cheaper alcohol, which this policy targets, generates a lower proportion of excise tax than the more expensive, so we can consider this a lower band on the excise tax revenue.

Consumption to harm

9. Relative risks

Relative risks were calculated for each of the health outcomes of interest at baseline, and each policy scenario using published relative risk equations [12,13]. The same relative risk equations are used for morbidity (or prevalence) and mortality. HIV risk is derived from a stepped function for mean drinking differing by socioeconomic status, intentional injuries and road injury from a continuous function of mean drinking differing by whether the individual binge drinks, liver cirrhosis and breast cancer from a continuous function of mean drinking, for breast cancer this is only for females (Table 10).

Table 11: Relative risk equations used

**Health Condition**	**Relative risk<br>Current drinkers**	**Relative risk former drinkers**	**ICD-10 codes**
**HIV**	Low SES<br>$RR = 2.99$<br>if x > 61/49 grams per day (males/females)<br>$RR = 1.94$ if x > 0<br>$RR = 1$ otherwise<br><br>Higher SES<br>$RR = 1.54$<br>if x > 61/49 grams per day (males/females)<br>$RR = 1$ otherwise	RR = 1	B20-24
**Intentional Injuries**<br><br>**(self-harm and interpersonal violence)**	Drinkers<br>$RR = \exp(00199800266267306 \,.\, x)$<br><br>Heavy episodic drinkers (HED)<br>$RR = \exp(00199800266267306 \,.\, x + 0.647103242058538)$	RR = 1	ICD-10 codes: X60 – Y09<br>Y35 –36<br>Y870<br>Y871
**Road Injury**<br><br>**(pedestrian, cyclist, motorcyclist, motor vehicle, other road)**	Drinkers<br>$RR = \exp(0.00299550897979837 \,.\, x)$<br><br>Heavy episodic drinking<br>$RR = \exp(0.00299550897979837 \,.\, x + 0.959350221334602)$	RR = 1	V01–04,<br>V06,<br>V09–80,<br>V87,<br>V89,<br>V99
**Breast Cancer**	Females only<br>$RR = \exp(0.01018 \,.\, x)$	RR = 1	C50
**Liver**	if x <= 1<br>$1 + x.\exp((\beta_1 + \beta_2).\sqrt{\frac{1 + 0.1699981689453125}{100}})$<br><br>If x > 1<br>$\exp((\beta_1 + \beta_2).\sqrt{\frac{x + 0.1699981689453125}{100}})$	RR = 3.26 for both females and males	K70, K74

	Female<br>b1 = 2.351821<br>b2 = 0.9002139<br><br>Male<br>b1 = 1.687111<br>b2 = 1.106413		
x = grams of alcohol consumed per day among current drinkers<br>HED = drinking 60 grams or more on one drinking occasion			

10. Potential impact fractions

Potential impact fractions (PIFs) were calculated by dividing relative risk under each policy by relative risk at baseline. These incorporated population weights and were computed by sex *(i)*, wealth group *(j)* and drinker group *(k)*.

$$PIF_{ijk} = \frac{\text{relative risk}_{ijk}\text{ (policy)}}{\text{relative risk}_{ijk}\text{ (baseline)}}$$

11. Socioeconomic gradients of ill health

Health outcomes in South Africa are not evenly distributed throughout the population, with the poor often bearing a higher burden of disease, depending on the illness. Data analysis was carried out using General Household Survey (GHS) data for 2018. The ordered choice regression model computed previously, using SADHS data, was applied to the GHS data to split the survey population into wealth quintiles compatible with the foundational dataset (SADHS). Percentage within each wealth quintile with the disease was computed (Table 11). Liver cirrhosis was not one of the health conditions included in the survey and breast cancer was not specifically included although the broader category of cancer was. Sensitivity analysis was carried out using alternative gradients.

Table 12: Raw count of General Household Survey data 2018

	**poorest**	**poorer**	**middle**	**richer**	**richest**
**15+ raw count**<br>(648 NAs)	4966	11462	14396	9633	7630
**HIV**<br>raw count<br>percentage	395<br>0.08	684<br>0.06	614<br>0.04	155<br>0.02	41<br>0.005
**Intentional injuries***<br>raw count<br>percentage	11<br>0.002	30<br>0.0027	24<br>0.0018	11<br>0.0012	3<br>0.0002
**Road injuries****<br>raw count<br>percentage	7<br>0.0016	26<br>0.0022	22<br>0.0016	32<br>0.0033	13<br>0.00015
**Cancer**<br>raw count<br>percentage	2<br>0.00038	27<br>0.0012	41<br>0.0026	27<br>0.0029	68<br>0.008
nb: percentages within each quintile were calculated incorporating the survey weights<br><br>* gunshot wounds; severe trauma due to violence, assault, beating; intentional poisoning; accidental poisoning; fire and burn; crime related injury – left out sports related, disability related and other<br>** motor vehicle -occupant, motor vehicle – pedestrian, bicycle related					

12. Distributing baseline deaths and cases and calculating probabilities

The deaths/cases (which come disaggregated by sex) at baseline is split between the five wealth quintiles using the GHS data to account for the socioeconomic gradient, as explained above. However, a preparatory step was necessary as the proportions of the population (using the SADHS proportions) in each quintile were not perfectly equal, for example for Q1, Q2, Q3, Q4, Q5 corresponded to 0.19, 0.19, 0.20, 0.21, 0.21 for females and 0.19, 0.20, 0.21, 0.20, 0.21 for males. The probability of death was calculated for each quintile first by assuming the population was split into quintiles of equal size. The total deaths/cases for each quintile using the SADHS proportions was then calculated by applying the relevant probability of death/cases for that part of the quintile which overlapped with the underlying equally sized quintile. This concept can be best illustrated on a graph.

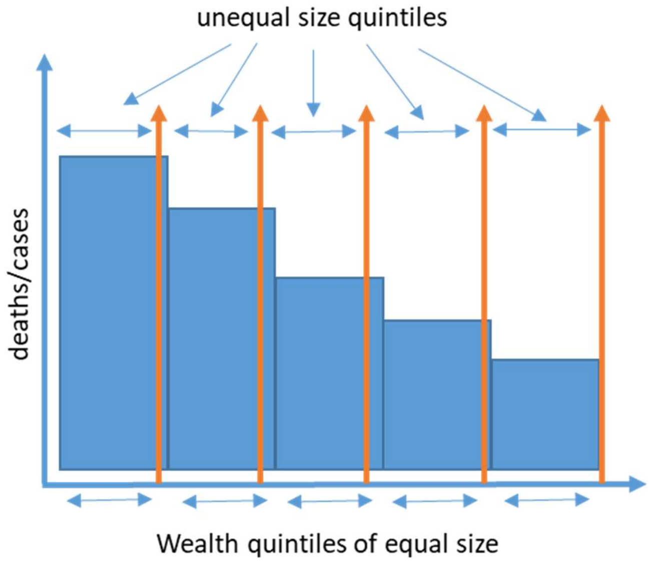

$$Number_{cases}(SADHS_{Q1}) = Pop_{SADHS} \quad \times Prob_{Equal}$$

$$Number_{cases}(SADHS_{Q2})$$
$$= (Pop_{EqualQ1} - \ Pop_{SADHSQ1}) \times \ Prob_{EqualQ1}$$
$$+ (Pop_{SADHSQ1} + \ Pop_{SADHS} \quad - Pop_{Equal} \quad) \times Prob_{Equal}$$

$$\ldots .\, and\ so\ on$$

The existence of relative risk equations implies that the baseline mortality/morbidity will also not be distributed equally between drinker groups, one would expect a higher proportion of the baseline cases to exist amongst heavy drinkers, followed by occasional binge, moderate then abstainers. In order for the baseline mortality/morbidity to vary by drinker group the total risk, for each disease, is calculated for each drinker group group, by sex and wealth quintile. The proportional share of risk between drinker groups is then calculated and used to distribute the mortality/morbidity, which has already been assigned to each quintile, between each drinker group within that quintile.

The model uses iHME data for deaths and cases of disease and population statistics (Statistics South Africa) from 2018. Life tables to get the probability of death by single year of age were only available for 2017 from iHME so these were used. The 2018 population is split proportionally into the sex/wealth/drinker groups using the SADHS proportions.

The probability of death for each disease is calculated for the baseline scenario and taken away from overall probability of death for each single year of age given in the life table to give a probability of death from non-modelled causes. This probability of death from non-modelled causes remains constant at every policy scenario. The probability of death from the five diseases of interest then vary according to the policy level and the corresponding potential impact fraction.

We model counterfactual population structure (i.e. in the absence of the policy) over 20 years, starting from 2018 using current population estimates from Statistics South Africa, plus birth projections for 2020 to 2023 and assume current age-, sex- and wealth-specific mortality rates remain constant [14]. Birth cohorts for years beyond 2023 are not modelled as they would not have reached the age at which we model alcohol consumption (15+) within the time horizon.

We create multistate life tables in which the population faces a probability of mortality for each of the five disease/injury conditions and for other cause mortality each year. This approach allows us to simulate prevalence of and mortality from multiple diseases simultaneously, assuming diseases are independent of one another. The model generates alternative population impact fractions (as above) for baseline and for each policy scenario. Using the relevant population impact fraction and rerunning the multistate life table enables a calculation of the difference between baseline and the policy.

13. Baseline health and lagged health impact

HIV, road injuries and intentional injuries realise the full impact of the reduction in drinking immediately whereas the health impact on liver cirrhosis and breast cancer are subject to lags in the effect, meaning the reduced drinking does not translate to a reduced health risk immediately [15]. Breast cancer starts to see an impact at year 11 and it is 20 years until full effect, liver cirrhosis sees some impact from year one but does not realise the full effect until year 20 (Appendix part 9).

The life tables for the 20 year time horizon are saved for each of the policy scenarios. They are then used in combination with the probability of having the disease and the potential impact fraction under each policy, to estimate the number of cases.

HIV, road injuries and intentional injuries realise the full impact of the reduction in drinking from the first year of the drinking reduction whereas liver cirrhosis and breast cancer are subject to lags in the effect. Breast cancer only starts to see an impact at year 11 and it is 20 years until full effect, liver cirrhosis sees some impact from year one but does not realise the full effect until year 20 (Table 12).

Table 13: Modelled time-lags by condition – proportion of overall change in risk experienced in each year following a change in consumption (Holmes et al., 2012)

Year	1	2	3	4	5	6	7	8	9	10	11	12	13	14	15	16	17	18	19	20
**Breast cancer**	0	0	0	0	0	0	0	0	0	0	10	20	30	40	50	60	70	80	90	100
**Liver Cirrhosis**	21	34	43	50	56	61	65	69	73	76	79	82	85	88	90	92	94	96	98	100

14. Hospital multipliers and costs

The prevalence of disease/injury at each policy scenario for each year of the 20 year time horizon was multiplied by the proportion who would then go on to receive hospital treatment (Table 13) and the relevant hospital cost applied (Table 14). The costs taken from the literature were increased by inflation where necessary to reach the baseline year of 2018. Future costs were discounted at 5% as recommended by the Department of Health in the guidelines for pharmacoeconomic submissions [16]. All sources were sense checked with a South African stakeholder with health economics expertise.

Table 14: Estimated multiplier from population prevalence to hospital admission

Condition	Multiplier (cases in population who go on to receive healthcare treatment)	Source
**HIV**	0.62	UNAIDS estimates that 62% of people living with HIV in 2018 in South Africa were on treatment [17]
**Intentional Injury**	0.41	Survey estimating trauma admissions [18] combined with iHME data from the same year to predict multipliers.
**Road injury**	0.19	Survey estimating trauma admissions [18] combined with iHME data from the same year to predict multipliers.
**Liver Cirrhosis**	0.5	Paper on liver cirrhosis in sub-Saharan Africa suggests 50% of patients are admitted to hospital with end-stage liver disease [19].
**Breast Cancer**	0.75	All studies found estimate what proportion present with late stage breast cancer (51%) but not what proportion never receive hospital treatment [20]. Therefore an estimate of 0.75 is used.

Table 15: Hospital costs and sources

Condition	Cost per patient	Source
**HIV**	R 3,318.62<br>(2017/18)	This is the annual cost. Taken from a systematic literature review of per patient costs of HIV services in South Africa [21]. There are many different levels of treatment, this cost is only for first-line treatment, so this is conservative.
**Intentional Injury**	R58,928<br>(2013)	This retrospective case note review included 143 violence related emergency hospital admissions from January to March 2013. Average inpatient stay was 9.8 days with treatments including emergency surgery, intensive care and resuscitation beds on admission [22].
**Road injury**	R56,592.17<br>(2012)	A prospective cohort study followed 100 patients admitted following a Road traffic injury between late 2011 and early 2012 at Edendale Hospital Pietermartizburg [23].
**Liver Cirrhosis**	R2,967<br>(2018)	50% multiplier used above comes from paper suggesting 50% of liver cirrhosis patients get admitted to hospital with end stage liver disease. Treatment for end stage liver disease includes<br><br>A specific study on liver cirrhosis was not found so general costs have been used from the district health baromenter. Expenditure per patient day equivalent (district hospitals) was R2967 (average taken from

		across the 9 provinces). This assumes just one patient day. Conservative. [24]
**Breast Cancer**	Early stage R14,915<br>Late stage R16,869<br>(2015)	This retrospective case review included 200 women at a government hospital in South Africa. The average cost is different depending on whether they were diagnosed at an early (56%) or late (44%) stage [25].

15. Sensitivity Analysis

Parameter	Central estimate	Alternative plausible values	Rationale	Results
**Price elasticities**	-0.53 moderate Q1, Q2<br>-0.31 moderate Q3, Q4, Q5<br><br>-0.29 occasional binge Q1, Q2<br>-0.17 occasional binge Q3, Q4, Q5<br><br>-0.24 heavy Q1, Q2<br>-0.14 heavy Q3, Q4, Q5	Scenario 1<br>-0.40 moderate<br>-0.22 occasional binge<br>-0.18 heavy<br><br>Scenario 2<br>-0.5 for high income drinkers (applied to quintiles 3, 4, 5)<br><br>-0.86 for low income drinkers (applied to quintiles 1,2 to be conservative)<br><br>Scenario 3<br>-0.8	Scenario 1<br>Applies estimates based only on drinker type, removing any wealth gradient.<br><br>Scenario 2<br>Estimates using NIDs data for two subsets of the population, the top 50% and bottom 50% of households by total household expenditure [10].<br><br>Scenario 3<br>Van Walbeek and Blecher [10] literature review of South African specific price elasticities found Selvanathan and Selvanathan [26] estimated -0.8 which corresponds closely to price elasticity estimates for beer (-0.8), wine (-0.9) and spirits (-0.9) produced by SALBA (2010).	Central estimates<br>Consumption - 4.40%<br>Spend 18.09 %<br>Lives saved 20,585<br>Cases saved 900,332<br><br>Scenario 1<br>Consumption - 4.50%<br>Spend 17.86%<br>Lives saved 18,717<br>Cases saved 825,935<br><br>Scenario 2<br>Consumption - 14.16%<br>Spend 5.4%<br>Lives saved 52,419<br>Cases saved 2,331,362<br><br>Scenario 3<br>Consumption - 17.96%<br>Spend 0.1 %<br>Lives saved 64,494<br>Cases saved 2,891,284

**Proportion of abstainers in the population**	82% female non-drinkers<br>45% male non-drinkers	67% female non-drinkers<br>36% male non-drinkers	Stakeholders have indicated scepticism about the prevalence of non-drinking reported in SADHS (and all alcohol studies). Currently the model only adjusts the consumption of those who report anything at all. We will increase the survey weightings of drinkers in the SADHS so that 67% of females do not drink and 36% of males. Based on a South African study which used both surveys and biomarkers [27].	Central estimates<br>Consumption - 4.40%<br>Spend 18.09 %<br>Lives saved 20,585<br>Cases saved 900,332<br><br>Alternative scenario<br>Consumption - 4.48%<br>Spend 17.77%<br>Lives saved 15,616<br>Cases saved 678,929
**HIV baseline estimates**	iHME 2018 estimates<br><br>female<br>77,499 deaths<br>4,772,473 cases<br><br>male<br>70,186 deaths<br>2,799,754 cases	Thembisa 2018 estimates<br><br>female<br>35,487 deaths<br>4,542,677 cases<br><br>male<br>36,345 deaths<br>2,578,747 cases	Stakeholders highlighted the difference between GBD estimates and local estimates for HIV deaths. The Thembisa model was built by local academics and is used by UNAIDs [28].	Central estimates<br>Lives saved 20,858<br>Cases saved 900,332<br>HIV lives saved 10,229<br>HIV cases averted 429,205<br><br>Alternative scenario<br>Lives saved 16,086<br>Cases saved 907,930<br>HIV lives saved 5,486<br>HIV cases averted 423,850
**Socioeconomic gradients of ill health**	HIV<br>Q1 (poorest) – 20%<br>Q2 – 36%<br>Q3 – 32%<br>Q4 – 9%<br>Q5 – 3%<br><br>Intentional Injury/Road Injury/Liver Cirrhosis<br>Q1 – 9%<br>Q2 – 29%	Scenario 1<br>Changing the liver cirrhosis gradient to match the one used for breast cancer	Scenario 1<br>Stakeholders indicated that for long-term conditions like cirrhosis wealthier groups could well be over-represented in SA. They suggested sensitivity analysis by applying values for a condition that is less concentrated amongst the poor.	Central estimates<br>Liver cirrhosis<br>lives saved/cases averted<br>Q1 133 / 3,528<br>Q2 432 / 11,298<br>Q3 295 / 7,801<br>Q4 288 / 7,639<br>Q5 82 / 2,158<br><br>Scenario 1

	Q3 – 26%<br>Q4 – 26%<br>Q5 – 10%<br><br>Breast cancer<br>Q1 – 7%<br>Q2 – 7%<br>Q3 – 22%<br>Q4 – 18%<br>Q5 – 47%	Scenario 2<br>HIV<br>Q1 (poorest) – 25%<br>Q2 – 22%<br>Q3 – 20%<br>Q4 – 18%<br>Q5 – 14%<br><br>Intentional injury/ Road injury/Liver cirrhosis<br>Q1 – 20%<br>Q2 – 20%<br>Q3 – 19%<br>Q4 – 20%<br>Q5 – 22%<br><br>Breast cancer<br>Q1 (poorest) – 21%<br>Q2 – 21%<br>Q3 – 20%<br>Q4 – 19%<br>Q5 – 18%	Scenario 2<br>Recent data from another South African survey is used to provide plausible alternative socioeconomic gradients across all the conditions used in the model [29].	Liver cirrhosis lives saved/cases averted<br>Q1 95 / 2509<br>Q2 104 / 2722<br>Q3 235 / 6203<br>Q4 200 / 5316<br>Q5 359 / 9563<br><br>Central estimates aggregate<br>lives saved / cases averted<br>Q1 4,088 / 176,663<br>Q2 7,375 / 313,360<br>Q3 4000 / 177,604<br>Q4 3,759 / 167,934<br>Q5 1,364 / 64,771<br><br>Scenario 2 aggregate<br>lives saved / cases averted<br>Q1 2,858 / 127,516<br>Q2 5,246 / 225,067<br>Q3 5,758 / 255,667<br>Q4 3,153 / 139,2253<br>Q5 3,969 / 197,191
**Discount rates for costs**	5% discount rate	Scenario 1<br>0% discount rate	Discount rate was changed to 0%	Central estimate<br>Health costs saved<br>R6.88 billion<br><br>Scenario 1<br>Health costs saved

				R11.10 billion
**Homebrew switching**	30%	Scenario 1<br>0%<br><br>Scenario 2<br>100%	The assumption that drinkers will make up 30% of the reduction in drinking recorded alcohol with homebrew comes from consultation with the stakeholders at workshop two. To test the importance of this assumption on the results a null impact and a 100% impact are introduced. 100% would mean that any homebrew drinkers will not receive any positive health impacts from the policy as all of their reduction in recorded alcohol will be replaced with homebrew alcohol.	Central estimate<br>Consumption - 4.40%<br>Lives saved 20,585<br>Cases saved 900,332<br><br>Scenario 1<br>Consumption - 4.56%<br>Lives saved 21,479<br>Cases saved 937,507<br><br>Scenario 2<br>Consumption - 4.03 %<br>Lives saved 19,156<br>Cases saved 844,471

16. Healthcare cost savings by quintile

Table 16: Health care costs for each of the three policy scenarios split by wealth quintile

	Q1	Q2	Q3	Q4	Q5
**R5 MUP**					
**HIV**	-R0.01	-R0.07	-R0.04	-R0.03	-R0.01
**Intentional injury**	R1.41	R5.22	R5.42	R12.8	R7.72
**Road injury**	R0.71	R2.73	R2.80	R6.39	R3.82
**Liver cirrhosis**	R0.02	R0.12	R0.11	R0.27	R0.14
**cancer**	R0.00	R0.00	R0.01	R0.05	R0.15
**R10 MUP**					
**HIV**	R162.00	R291.00	R71.10	R8.72	R33.3
**Intentional injury**	R495.57	R801.23	R1150.94	R1487.35	R369.03
**Road injury**	R232.98	R399.34	R520.70	R658.80	R163.64
**Liver cirrhosis**	R3.03	R9.64	R6.62	R6.45	R1.86
**cancer**	R0.30	R0.22	R0.80	R0.93	R1.75
**R15 MUP**					
**HIV**	R403.19	R618.29	R190.50	R79.85	R64.67
**Intentional injury**	R1136.23	R2029.50	R2558.09	R2350.20	R1014.96
**Road injury**	R536.83	R1013.46	R1173.35	R1080.17	R4618.76
**Liver cirrhosis**	R7.42	R23.50	R17.60	R15.20	R4.51
**cancer**	R0.76	R0.65	R2.24	R2.30	R4.65

References

1. Kehoe T, Gmel G, Shield KD, Gmel G, Rehm J. Determining the best population-level alcohol consumption model and its impact on estimates of alcohol-attributable harms. *Population health metrics* 2012; **10**(1): 6.
2. Rehm J, Kehoe T, Gmel G, Stinson F, Grant B, Gmel G. Statistical modeling of volume of alcohol exposure for epidemiological studies of population health: the US example. *Population Health Metrics* 2010; **8**(1): 3.
3. Meier PS, Meng Y, Holmes J, et al. Adjusting for unrecorded consumption in survey and per capita sales data: quantification of impact on gender-and age-specific alcohol-attributable fractions for oral and pharyngeal cancers in Great Britain. *Alcohol and Alcoholism* 2013; **48**(2): 241-9.
4. Probst C, Shuper PA, Rehm J. Coverage of alcohol consumption by national surveys in South Africa. *Addiction* 2017; **112**(4): 705-10.
5. Gmel G, Shield KD, Kehoe-Chan TA, Rehm J. The effects of capping the alcohol consumption distribution and relative risk functions on the estimated number of deaths attributable to alcohol consumption in the European Union in 2004. *BMC medical research methodology* 2013; **13**(1): 24.
6. Brennan A, Meier P, Purshouse R, et al. The Sheffield alcohol policy model–a mathematical description. *Health economics* 2015; **24**(10): 1368-88.
7. Venables WN, Ripley BD. Modern Applied Statistics with S. Fourth Edition. New York: Springer; 2002.
8. SA Wine Industry Information and Systems. SA WINE INDUSTRY 2019 STATISTICS NR 44. South Africa, 2019.
9. van Walbeek C, Chelwa G. Using price-based interventions to reduce abusive drinking in the Western Cape Province. 2019.
10. Van Walbeek C, Blecher E. The economics of alcohol use, misuse and policy in South Africa. 2014. http://www.tobaccoecon.uct.ac.za/sites/default/files/image_tool/images/405/People/the-economics-of-alcohol-policy-in-south-africa.pdf (accessed 10/03/2019.
11. Treasury N. Budget Review 2020. In: Treasury N, editor.; 2020.
12. Shield K, Manthey J, Rylett M, et al. National, regional, and global burdens of disease from 2000 to 2016 attributable to alcohol use: a comparative risk assessment study. *The Lancet Public Health* 2020; **5**(1): e51-e61.
13. Probst C, Parry CD, Rehm J. HIV/AIDS mortality attributable to alcohol use in South Africa: a comparative risk assessment by socioeconomic status. *BMJ open* 2018; **8**(2): e017955.
14. Rebublic of South Africa. Mid-year population estimates, 2019. In: Statistics South Africa, editor.; 2019.
15. Holmes J, Meier PS, Booth A, Guo Y, Brennan A. The temporal relationship between per capita alcohol consumption and harm: a systematic review of time lag specifications in aggregate time series analyses. *Drug and alcohol dependence* 2012; **123**(1-3): 7-14.
16. Republic of South Africa. Guidelines for Pharmacoeconomic Submissions. In: Department of Health, editor.: Government Gazette; 2012.
17. UNAIDS. UNAIDS South Africa: Overview. 2020. https://www.unaids.org/en/regionscountries/countries/southafrica (accessed 27/02/2020.
18. Matzopoulos RG, Prinsloo M, Butchart A, Peden MM, Lombard CJ. Estimating the South African trauma caseload. *International Journal of Injury Control and Safety Promotion* 2006; **13**(1): 49-51.
19. Vento S, Dzudzor B, Cainelli F, Tachi K. Liver cirrhosis in sub-Saharan Africa: neglected, yet important. *The Lancet Global Health* 2018; **6**(10): e1060-e1.
20. Joffe M, Ayeni O, Norris SA, et al. Barriers to early presentation of breast cancer among women in Soweto, South Africa. *PloS one* 2018; **13**(2): e0192071.
21. Meyer-Rath G, van Rensburg C, Chiu C, Leuner R, Jamieson L, Cohen S. The per-patient costs of HIV services in South Africa: Systematic review and application in the South African HIV Investment Case. *PloS one* 2019; **14**(2): e0210497.

22. Bola S, Dash I, Naidoo M, Aldous C. Interpersonal violence: quantifying the burden of injury in a South African trauma centre. *Emergency medicine journal* 2016; **33**(3): 208-12.
23. Parkinson F, Kent S, Aldous C, Oosthuizen G, Clarke D. The hospital cost of road traffic accidents at a South African regional trauma centre: A micro-costing study. *Injury* 2014; **45**(1): 342-5.
24. Health Systems Trust. District Health Barometer 2018/19, 2020.
25. Guzha N, Thebe T, Butler N, Valodia P. Development of a method to determine the cost of breast cancer treatment with chemotherapy at Groote Schuur Hospital, Cape Town, South Africa. *South African Medical Journal* 2020; **110**(4): 296-301.
26. Selvanathan S, Selvanathan EA. The Demand for Alcohol, Tobacco and Marijuana: International Evidence. Aldershot, England: Ashgate Publishing; 2005.
27. Pisa PT, Vorster HH, Kruger A, Margetts B, Loots DT. Association of alcohol consumption with specific biomarkers: A cross-sectional study in south africa. *Journal of health, population, and nutrition* 2015; **33**(1): 146.
28. Johnson LF, May MT, Dorrington RE, et al. Estimating the impact of antiretroviral treatment on adult mortality trends in South Africa: A mathematical modelling study. *PLoS medicine* 2017; **14**(12): e1002468.
29. Kabudula CW, Houle B, Collinson MA, et al. Socioeconomic differences in mortality in the antiretroviral therapy era in Agincourt, rural South Africa, 2001–13: a population surveillance analysis. *The Lancet Global Health* 2017; **5**(9): e924-e35.
