# Supplementary material for: Effects of minimum unit pricing for alcohol in South Africa across different drinker groups and wealth quintiles: a modelling study
Source: BMJ Open. 2021 Aug 9;11(8):e052879. doi: 10.1136/bmjopen-2021-052879 (PMC8354280; doi:10.1136/bmjopen-2021-052879)
Supplement: Author's manuscript [file bmjopen-2021-052879.draft_revisions.pdf]

BMJ Open

BMJ Open is committed to open peer review. As part of this commitment we make the peer review history of every article we publish publicly available.

When an article is published we post the peer reviewers' comments and the authors' responses online. We also post the versions of the paper that were used during peer review. These are the versions that the peer review comments apply to.

The versions of the paper that follow are the versions that were submitted during the peer review process. They are not the versions of record or the final published versions. They should not be cited or distributed as the published version of this manuscript.

BMJ Open is an open access journal and the full, final, typeset and author-corrected version of record of the manuscript is available on our site with no access controls, subscription charges or pay-per-view fees (http://bmjopen.bmj.com).

If you have any questions on BMJ Open's open peer review process please email info.bmjopen@bmj.com

Effects of minimum unit pricing for alcohol in South Africa across different drinker groups and wealth quintiles: a modelling study.

Journal:	*BMJ Open*
Manuscript ID	bmjopen-2021-052879
Article Type:	Original research
Date Submitted by the Author:	28-Apr-2021

Complete List of Authors:	Gibbs, Naomi; The University of Sheffield Faculty of Medicine Dentistry and Health, School of Health and Related Research<br>Angus, Colin; The University of Sheffield School of Health and Related Research, ScHARR<br>Dixon, Simon; University of Sheffield School of Health and Related Research<br>Parry, Charles; South African Medical Research Council, Alcohol & Drug Abuse Research Unit<br>Meier, Petra; University of Glasgow

Keywords:	Epidemiology < TROPICAL MEDICINE, HEALTH ECONOMICS, PUBLIC HEALTH, Health policy < HEALTH SERVICES ADMINISTRATION & MANAGEMENT, HIV & AIDS < INFECTIOUS DISEASES

BMJ

I, the Submitting Author has the right to grant and does grant on behalf of all authors of the Work (as defined in the below author licence), an exclusive licence and/or a non-exclusive licence for contributions from authors who are: i) UK Crown employees; ii) where BMJ has agreed a CC-BY licence shall apply, and/or iii) in accordance with the terms applicable for US Federal Government officers or employees acting as part of their official duties; on a worldwide, perpetual, irrevocable, royalty-free basis to BMJ Publishing Group Ltd ("BMJ") its licensees and where the relevant Journal is co-owned by BMJ to the co-owners of the Journal, to publish the Work in this journal and any other BMJ products and to exploit all rights, as set out in our licence.

The Submitting Author accepts and understands that any supply made under these terms is made by BMJ to the Submitting Author unless you are acting as an employee on behalf of your employer or a postgraduate student of an affiliated institution which is paying any applicable article publishing charge ("APC") for Open Access articles. Where the Submitting Author wishes to make the Work available on an Open Access basis (and intends to pay the relevant APC), the terms of reuse of such Open Access shall be governed by a Creative Commons licence – details of these licences and which Creative Commons licence will apply to this Work are set out in our licence referred to above.

Other than as permitted in any relevant BMJ Author's Self Archiving Policies, I confirm this Work has not been accepted for publication elsewhere, is not being considered for publication elsewhere and does not duplicate material already published. I confirm all authors consent to publication of this Work and authorise the granting of this licence.

For peer review only

TITLE: EFFECTS OF MINIMUM UNIT PRICING FOR ALCOHOL IN SOUTH AFRICA ACROSS DIFFERENT DRINKER GROUPS AND WEALTH QUINTILES: A MODELLING STUDY.

AUTHORS

Naomi K Gibbs, Colin Angus, Simon Dixon, Charles DH. Parry, Petra S. Meier

AUTHOR AFFILIATIONS

N K Gibbs, C Angus: School of Health and Related Research, University of Sheffield, UK

Prof S Dixon: School of Health and Related Research, University of Sheffield, UK. Honorary Professor, Priority Cost Effective Lessons for Systems Strengthening, South Africa (PRICELESS SA), School of Public Health, Faculty of Health Sciences, University of the Witwatersrand

Prof C D H Parry: Alcohol, Tobacco and Other Drug Research Unit, South African Medical Research Council, South Africa

Prof P S Meier: MRC/CSO Social and Public Health Sciences Unit, University of Glasgow

CORRESPONDANCE TO:

Naomi Gibbs, ScHARR, University of Sheffield, S1 4DA, UK

n.gibbs@sheffield.ac.uk

0114 222 5454

WORDCOUNT:

Abstract: 252

Main text: 3050

ABBREVIATIONS

MUP: Minimum unit pricing

HIV: Human immunodeficiency virus

VAT: Value added tax

SADHS: South African Demographic and Health Survey

IAC: International Alcohol Control study

PIF: Potential impact fraction

For peer review only

ABSTRACT

Objectives

To quantify the potential impact of Minimum Unit Pricing (MUP) for alcohol on drinking, spending and health in South Africa. We provide these estimates disaggregated by different drinker groups and wealth quintiles.

Design

We developed an epidemiological policy appraisal model to estimate the effects of MUP across sex, drinker groups (moderate, occasional binge drinker, heavy) and wealth quintiles. Stakeholder interviews and workshops informed model development and ensured policy relevance.

Setting

South African adult drinking population.

Participants

The adult (aged 15+) population of South Africa in 2018, stratified by drinking group and wealth quintiles, with a model time horizon of 20 years.

Main outcome measures

Change in standard drinks (SD) (12 grams of ethanol) consumed, weekly spend on alcohol, annual number of cases and deaths for five alcohol-related health conditions, reported by drinker groups and wealth quintile.

Results

We estimate that a MUP of R10 per SD would lead to an immediate reduction in consumption of 4.50%. The absolute reduction is greatest for heavy drinkers (-78 SD/year), followed by moderate drinkers (-22) and then occasional binge drinkers (-21). Over 20 years we estimate 23,011 fewer deaths and 1,010,354 cases averted across the five health modelled harms.

Poorer drinkers would see greater impacts from the policy (consumption: -5.72% in poorest, -4.16% in richest quintile). Amongst the heavy drinkers 78% of the cases averted and 79% of the lives saved accrue to the bottom three wealth quintiles.

Conclusions

We estimate that MUP would reduce alcohol consumption in South Africa, improving health outcomes whilst raising retail and tax revenue. Consumption and harm reductions would be greater in poorer groups.

For peer review only

ARTICLE SUMMARY

Strengths and limitations of this study

- This study presents the first epidemiological policy appraisal model of a minimum unit price applied to South Africa. Previous similar modelling work has been limited to high-income countries.
- Our model provides equity relevant information by presenting results disaggregated by wealth quintiles, allowing for a more nuanced consideration of the potential impact of the policy.
- Our model has also benefited from a thorough programme of stakeholder engagement ensuring contextual and policy relevance.
- A key limitation is the alcohol data which is drawn from a relatively small sample in one locality. Further research would benefit from improved pricing data, specifically the different prices paid for alcohol by different population groups.
- Secondly the model has not explored the financial impact on the poorest groups beyond increased alcohol expenditure. Further research should include exploration of broader financial benefits such as reduced private expenditure on healthcare or improved labour market outcomes.

For peer review only

BACKGROUND

In South Africa there are high levels of reported abstinence coupled with high levels of binge drinking amongst those who do drink, resulting in significant levels of alcohol-related harm [1]. This harm is not distributed evenly throughout society with the lower socioeconomic groups experiencing higher levels of harm, particularly for infectious diseases such as HIV [2]. The periodic prohibition of alcohol during the covid-19 lockdown demonstrates political leader's acceptance that alcohol causes harm to South Africa and signals a potential willingness to take strong action [3]. Provincial governments, such as the Western Cape, are considering a number of alcohol policy approaches, including the introduction of Minimum Unit Pricing (MUP) [4].

MUP is a policy whereby a legal floor price is introduced, below which a fixed volume of ethanol cannot be sold to the public. It has already been introduced in several areas which experience high levels of alcohol harm, including Scotland and Australia's Northern Territory. Evidence suggests that MUP has been effective at reducing alcohol consumption, particularly among the heaviest drinkers, as they commonly drink the very cheap alcohol targeted by this policy [5,6].

A limitation of transferring the current evidence for MUP is its focus on high income countries. South Africa has very different drinking patterns, it has a very different harm profile with infectious disease and injury contributing significantly to the burden of alcohol, it has an informal sector which is challenging to capture and it has very high levels of income inequality.

The current alcohol landscape is rooted in the country's recent political history. In 1926 apartheid legislation prohibited African and Indian access to licensed premises or employment by licence holders. As a result when the democratically elected government took power in 1994 they inherited a significant number of shebeens. Shebeens are (largely) unlicensed bars or pubs, found in townships, often open late and with a reputation for violence and risky sexual behaviour. Homebrew (mainly beer made from sorghum or other ingredients such as pineapple) can be purchased from shebeens along with other types of branded alcohol supplied by large alcohol manufacturers and mainly distributed through larger licensed outlets using bulk discounts. Although beer is the most popular drink, the consumption of large quantities of cheap wine is also prevalent and can be linked back to farm labourers being paid in cheap wine [7].

The South African government currently use alcohol excise tax to compensate for some of the social costs they attribute to alcohol consumption [8,9]. The system is based on targets for the proportion of the price that constitutes tax (excise tax plus Value Added Tax (VAT)), this varies by drink type with wine lowest followed by beer then spirits. The government have indicated a willingness to innovate and pursue public health improvements via fiscal policy with the introduction of a sugar tax in 2018. However, in a country with high levels of socioeconomic inequality there are concerns regarding possible financial impact of pricing policies on the poorest groups [10]. Evidence on public health pricing policies often fails to consider distributional impact by income-groups [11].

When designing public health economic models for unique policy contexts ongoing engagement with local stakeholders is essential. The purpose of engagement is twofold: to shape the direction of the research using expert local knowledge (including understanding the problem, guiding model development and ensuring policy relevance); and to provide channels for communication creating potential for the evidence to contribute to policy design [12-14].

We aimed to: 1) present estimates of the change to alcohol consumption, individual expenditure, retail and tax revenue following the introduction of a South African MUP, using a purpose built model, 2) estimate the impact on a limited number of alcohol-related health conditions and associated healthcare costs, 3) explore the potential equity implications via the demonstration of impact by both drinker group and wealth quintile, 4) highlight parameters that are particularly influential to the results and areas that require further research.

For peer review only

METHODS

We built an epidemiological policy appraisal model coded in R (code available here), using a comparative risk assessment approach with multistate life tables [15]. A stakeholder mapping exercise was carried out following scoping conversations with three academic experts from three South African institutions. Following this, a shortlist of policy professionals, civil society members and local academics was drawn up and checked via the scientific and ethical review process. They were engaged via scoping interviews and three workshops, at the beginning, middle and end of the modelling process. Stakeholders informed key decisions including the specific policy to simulate, levels of the MUP, health outcomes of interest, assumptions on homebrew switching behaviour and validation of our choice of data sources.

Two distinct sections of the model are defined (Figure 1):

I. Price to consumption: Baseline prices and consumption are estimated for drinker groups (heavy drinkers, occasional binge drinkers, moderate drinkers) and wealth groups. Following a change in price the new price and subsequent consumption levels are estimated. This accounts for both mean and peak weekly alcohol consumption. It also estimates the individual proportion of alcohol drunk that is homebrew.
II. Consumption to harm: The relationship between mean and peak consumption and alcohol-related harm and associated costs are estimated.

FIGURE 1

There is no single dataset that can provide all the required data for the model and thus a combination of survey datasets, market research data and evidence from published literature is used (Figure 2).

FIGURE 2

Price to consumption

Baseline consumption and prices

Our model starts by estimating mean and peak alcohol consumption at current alcohol prices at the individual level. We categorise drinkers into three exhaustive and mutually exclusive groups; moderate (less than 15 standard drinks per week); occasional binge (less than 15 drinks per week but more than 5 on one occasion); and heavy (15 or more drinks per week). A standard drink in South Africa is currently 15ml or 12 grams of pure ethanol. We generate price distributions for wealth and drinker groups using IAC data. Alcohol is treated as one commodity due to data constraints. A detailed description of the above is given in the appendix parts 1 to 6.

Applying a minimum unit price

A government policy of legislating for a MUP of R5, R10 and R15 per South African standard drink is introduced. Prices below the MUP threshold are increased to the threshold, while products above are unaffected. We do not include prices for homebrew. The mean price per standard drink for each wealth/drinker group before and after the policy is used to calculate the percentage change in price.

Elasticity of demand for alcohol

The change in price is translated into a change in individual consumption using an elasticity of demand for alcohol. We use previously published elasticities for South Africa, calculated separately by drinker group: -0.4, -0.22, -0.18 for moderate, occasional binge and heavy drinkers respectively [4].

For peer review only

Those that drink both recorded alcohol and homebrew switch some of their drinking to homebrew. Stakeholders indicated 30% of the reduction in recorded alcohol would be a suitable level of switching, varied between 0% and 100% in the sensitivity analysis.

Individual spend, tax and retail revenue

Total retail spend is computed by aggregating population weighted individual spend. This figure is increased by 1.25 (100/80) as consumption is calibrated to 80% of official sales volume data.

VAT is calculated as 15% of the total aggregate spend. Excise tax is calculated by starting with the total 2018 alcohol excise tax revenue from the Treasury Budget Report [9]. This is adjusted by percentage change in volume of alcohol sold (we use a fixed ratio between volume and excise tax). Retail revenue is calculated by taking VAT and excise taxation away from total spend.

Consumption to harm

Relative risks and potential impact fractions

We use published estimates of relative risks associated with different levels of alcohol consumption (Appendix 7). We then use these to calculate relative risks for each outcome for each individual.

We utilise Potential Impact Fractions (PIFs), a widely-used approach in epidemiological modelling to estimate the impact of a change in exposure to risk on a change in outcomes [16]. We incorporate population weights and compute the PIFs by sex, wealth group and drinker groups (Appendix 8).

Baseline health

Baseline deaths and cases (population prevalence) of the five disease and injury conditions (HIV, road injury, intentional injury, liver cirrhosis and breast cancer) are apportioned by drinker group, sex and wealth quintile. The probability of death for each disease is calculated for baseline and taken away from overall probability of death for each single year of age given in the life table to give a probability of death from non-modelled causes. This probability of death from non-modelled causes remains constant at every policy scenario. The probability of death from the five diseases of interest then varies according to the policy level and the corresponding potential impact fraction. A more detailed description is given in the appendix 9 to 10.

Projecting the population

We model counterfactual population structure (i.e. in the absence of the policy) over 20 years, starting from 2018 [17]. We create multistate life tables in which the population faces a probability of mortality for each of the five disease/injury conditions and for non-modelled causes each year. The model generates alternative population impact fractions (as above) for baseline and for each policy scenario. Using the relevant population impact fraction and rerunning the multistate life table enables a calculation of the difference between baseline and the policy. HIV, road injuries and intentional injuries realise the full impact of the reduction in drinking immediately whereas the health impact on liver cirrhosis and breast cancer are subject to lags in the effect [18] (Appendix 11).

The life tables for the 20 year time horizon are used in combination with the probability of having the disease and the potential impact fraction under each policy, to estimate the number of cases.

Hospital costs

Prevalence of disease/injury at each policy scenario for each year of the model is multiplied by the proportion who receive hospital treatment and the relevant hospital cost applied (Appendix 12). We convert all costs to 2018 prices using the Consumer Price Index [19]. Future costs are discounted at 5%.

Sensitivity analysis

We explore key uncertainties in the model using scenario analysis informed by previous published alcohol modelling work [20], our knowledge of the limitations of the data and stakeholder input. For each alternative scenario, relevant results are compared with central estimates. The key parameters

For peer review only

explored are elasticities, proportion of abstainers, HIV baseline estimates, socioeconomic gradients of health, proportion of switching to homebrew and discount rates for costs. (Appendix 13).

Patient and Public Involvement

Patients were not involved in this study.

For peer review only

RESULTS

Estimated consumption and spend impact

Our findings are presented primarily for R10 but with some comparisons across all three pricing levels. The policy appraisal results are reported by quintile further disaggregated by drinker group (Table 1). Drinking prevalence increases with wealth (27% up to 38%) as does the prevalence of heavy drinking, ranging from 14% amongst the poorest up to 20% in the richest quintile. Amongst all drinker groups mean consumption is either similar or demonstrates no clear pattern between wealth quintiles. On aggregate there is a gradient in average baseline weekly spend with the rich paying an average R254·70 per week compared to R146·13 in the lowest wealth group.

We estimate, for a MUP of R10, an immediate reduction in population alcohol consumption of 4.50% (−49.25 units per drinker per year) and an increase in spend of 17.83%. Moderate drinkers show the greatest percentage decrease in their drinking, followed by occasional binge then heavy drinkers (-8.89%, -4.29%, -4.48%). However this translates to a larger absolute reduction in consumption for heavier drinkers (-77.66 standard drinks per year) than either moderate or occasional binge drinkers (-21.75, -21.35).

For peer review only

Table 1: Consumption and spend R10 policy estimates

	Overall	Q1	Q2	Q3	Q4	Q5
**All drinkers**						
**n (%)***	3311 (33%)	551 (27%)	690 (30%)	823 (33%)	685 (35%)	562 (38%)
Baseline consumption (standard drinks per week)	21·05	20·56	21·36	20·91	21·73	20·67
Baseline spending (R per week)	R207.14	R146·13	R192·71	R186·45	R229·31	R254·70
Change in consumption (%)	-4·50%	-5·72%	-4·74%	-4·90%	-4·43%	-4·16%
Change in consumption (standard drinks per week)	-0·95	-1·11	-0·97	-0·98	-0·92	-0·82
Change in spending (R per week)	R36·93	R36·63	R36·45	R35·51	R39·28	R39·78
**Moderate**						
**n (%)***	1336 (12%)	206 (10%)	272 (13%)	354 (12%)	273 (13%)	231 (15%)
Baseline consumption (standard drinks per week)	5·12	5·07	5·56	5·05	4·87	5·09
Baseline spending (R per week)	R50.69	R42·94	R49·39	R44·87	R54·59	R57·80
Change in consumption (%)	-8·89%	-8·88%	-9·42%	-9·42%	-8·36%	-8·45%
Change in consumption (standard drinks per week)	-0·42	-0·41	-0·48	-0·43	-0·38	-0·40
Change in spending (R per week)	R5·67	R5·15	R5·74	R5·23	R5·79	R6·17
**Occasional Binge**						
**n (%)***	433 (4%)	76 (4%)	89 (4%)	109 (5%)	91 (4%)	68 (4%)
Baseline consumption (standard drinks per week)	9·57	9·62	9·86	9·45	9·30	9·72
Baseline spending (R per week)	R97.67	R67·27	R91·42	R93·45	R121·17	R107·84
Change in consumption (%)	-4·48%	-7·47%	-3.26·%	-5·32%	-2·47%	-4·33%
Change in consumption (standard drinks per week)	-0·41	-0·67	-0·31	-0·48	-0·22	-0·40
Change in spending (R per week)	R14·34	R19·14	R11·26	R16·26	R10·59	R15·04
**Heavy**						
**n (%)***	1542 (16%)	269 (14%)	329 (14%)	360 (16%)	321 (17%)	263 (20%)
Baseline consumption (standard drinks per week)	36·31	34·56	39·39	35·95	37·67	34·58
Baseline spending (R per week)	R356.11	R240·99	R355·42	R317·96	R389·25	R432·16
Change in consumption (%)	-4·29%	-5·27%	-4·25%	-4·42%	-4·19%	-3·70%
Change in consumption (standard drinks per week)	-1·49	-1·73	-1·60	-1·52	-1·51	-1·23
Change in spending (R per week)	R68·10	R62·93	R71·96	R63·28	R71·80	R69·60

Data for 10,336 survey respondents.
*Numbers refer to absolute sample size, percentages refer to sample size after survey weights have been applied.
Q1 = poorest

For peer review only

Our model estimates that there would be an increase in individual spend of R32.06 billion in the year following the introduction of the policy. The government would see an increase in VAT as a result of the increased prices although a reduction in excise taxation due to the reduced volume of alcohol sold. Retail revenue would also increase (Table 2).

Table 2: Aggregate spend, taxation and retail revenue

Change from baseline in billion Rand, per year			
	**R5 MUP**	**R10 MUP**	**R15 MUP**
**Individual spend**	R1·17	R32·06	R76·65
**Taxation**			
VAT	R0·15	R4·18	R10·00
Excise tax	-R0·04	-R1·26	-R3·44
**Retail revenue**	R1·06	R29·14	R70·09

Estimated health impact

Across the five health conditions included in the model a R10 minimum price estimates 23,011 lives saved and 1,010,354 cases averted of the disease/injury conditions over the 20 year time horizon. For R5 (R15) the estimates are 114, (46,948) lives saved and 4,810, (2,102,312) cases averted respectively. The impact differs by drinker group and by wealth quintile (Figure 3). The greatest health benefits accrue to the heaviest drinkers, with the dominant effect related to HIV infections, especially in the bottom three quintiles. Amongst the heavy drinkers 78% of the cases averted and 79% of the lives saved accrue to the bottom three quintiles. Occasional binge drinkers achieve most of their positive health impact via a reduction in interpersonal violence and road injury as both of these conditions are linked to binge drinking. There is a small increase of HIV incidence amongst occasional binge drinkers. The high prevalence of HIV is the source of an important competing risk and the avoidance of death related to acute conditions leads to longer exposure to the risk of HIV infection. As expected the cases saved of liver cirrhosis are amongst the heavy drinkers as this condition relates to heavy drinking in the long term.

FIGURE 3

Health care cost savings accrue over the 20 years and are greatest for intentional injury (Table 3).

Table 3: Healthcare cost savings over 20 years, millions

	R5 MUP	R10 MUP	R15 MUP
Antiretroviral therapy costs	+R0.15	-R643.03	-R1352.07
Intentional injury hospital costs	-R37.86	-R4811.91	-R9732.23
Road injury hospital costs	-R19.05	-R2202.85	-R4538.01
Liver cirrhosis hospital costs	-R0.76	-R28.08	-R69.84
Breast cancer hospital costs	-R0.29	-R4.89	-R12.86

Results across policy levels

Comparing across the three policy levels demonstrates the relative impact between wealth quintiles remains largely consistent as the MUP level increases for moderate and occasional binge drinkers (Figure 4) although for heavy drinkers the health benefit shifts even more towards the bottom three quintiles at R15.

For peer review only

FIGURE 4

The sensitivity analysis that produced the most variable results were the alternative elasticity estimates. Both alternative sceanrios produced much greater consumption impacts (-14%, – 18%) coupled with much smaller increases in individual spend. All other results are included in appendix 13.

CONCLUSION

Our analysis estimates that MUP may offer an effective approach to reducing alcohol consumption and related harm in South Africa. For a MUP of R10, we estimate an immediate reduction in consumption of 4.50%, increase in individual spend of 17.83% and an increase in retail revenue and taxation. In terms of health impact we estimate 23,011 lives saved and 1,010,354 cases averted in total across HIV, intentional injury, road injury, liver cirrhosis and breast cancer over 20 years. Regarding the equity impact our model estimates the distribution of health outcomes is generally pro poor, critically important given these groups also see the greatest relative increase in their alcohol expenditure. Our research corresponds to mechanisms, such as greater impact with a rising MUP threshold and greater impact on the poor, found in the international literature [21].

We add to the South African minimum pricing evidence currently available [4] by incorporating health outcomes, accommodating homebrew and exploring differential impact by wealth groups. van Walbeek and Chelwa [4] suggest both a higher reduction in consumption and relatively higher impact on the heaviest drinkers than we estimate here. This is in part due to different price estimates and crucially far more heterogeneous price distributions between drinker groups which outweigh the impact of the price elasticities. This suggests that our findings may present a conservative estimate.

Our study has a number of strengths relevant to providing policy relevant research in LMICs. In the absence of detailed market research purchasing data we demonstrate how survey, administrative data and the academic literature can be used, in partnership with local stakeholders, to build a contextually relevant epidemiological policy appraisal model.

A further strength is our focus on stakeholder engagement from project inception increasing the likelihood of findings being taken into consideration during policy decision making [22]. MUP was chosen as the policy to model as it was seen as both innovative and potentially well targeted for the South African heavy drinking culture. Stakeholders were pleased the estimates combined improved health with increased taxation and increased retail revenue, as supporting business was considered politically important. The financial cost of MUP is borne by drinkers and there were concerns about how this may impact poorer groups. However, in a country which suffers a very high degree of alcohol related harm coupled with very high abstinence levels, it may be considered an improvement by some that drinkers, rather than the whole of society, pay.

A limitation of our study is the lack of high quality pricing data for South Africa. Previous studies in HIC have found that moderate drinkers, even those on lower incomes, purchase relatively little cheap alcohol [21], while the price data used in our model suggests that all drinker groups purchase some cheap alcohol. It is unclear whether this is a true reflection of alcohol purchasing patterns in SA or a limitation of the data. Although we adjusted the off-trade wine prices to be consistent with industry sources we know that the proportion of wine in the survey is less than the market share. As wine constitutes some of the cheapest available alcohol a MUP may have a bigger impact than our estimates suggest.

Related to this limitation is the treatment of all drinks as one commodity. South African evidence has suggested cheap wine has a much higher price elasticity than other drink types [8]. If cheap wine is the

drink primarily effected then this elasticity would lead to less of an increase in individual spend, potentially even a saving, smaller gains to retailers and less of a loss to excise tax revenue as wine enjoys substantially lower rates.

We recommend two avenues for further research. Firstly, the collection of improved pricing data, specifically the different prices paid for alcohol by different population groups, to explore further the most appropriate level of MUP. Secondly, the exploration of the financial impact on the poorest groups including any financial benefits such as reduced expenditure on healthcare or improved labour market outcomes.

In conclusion, our model estimates minimum pricing would reduce alcohol consumption in South Africa, improving health outcomes whilst raising retail and tax revenue. Consumption and harm reductions would be greater in poorer compared to richer groups. We estimate that minimum pricing is a targeted policy that has the potential to bring health and financial benefits to a country which suffers a very high burden of alcohol-related harm.

Acknowledgments

We would like to thank all stakeholders who have shaped the research. This includes contributors from National Government, Western Cape Department of the Premier, Western Cape Liquor Authority, South African Alcohol Policy Alliance, Douglas Murray Trust, Khayelitsha Community Health Forum, Violence Protection through Urban Upgrading, Research Unit of the Economics of Excisable Products at University of Cape Town, South African Medical Research Council and the University of the Western Cape. We would also like to thank Charlotte Probst and Nick Stacey for their scientific review of the work.

Contributors

All authors conceptualised the study. NKG completed the modelling and stakeholder engagement under the supervision of CA, SD, PSM and CDHP. NKG wrote the first draft. All authors refined various drafts of the manuscript and approved the final version.

Competing interests

We declare no competing interests.

Data sharing statement

No additional data available.

Funding

This work was supported by the Wellcome Trust Doctoral Training Centre in Public Health Economics and Decision Science [108903/Z/19/Z] and the University of Sheffield [No award number]. Also the South African Medical Research Council [No award number]. The funders of the study had no role in the study. All authors had full access to all the data in the study and were responsible for the decision to submit the article for publication.

Ethics committee approval

Ethical approval for engaging with stakeholders was granted by the South African Medical Research Council (Protocol ID: EC005-4/2019) and the School of Health and Related Research at the University of Sheffield, UK (Reference Number: 023357). All data for the model came from secondary sources and was managed according to an approved information governance plan.

For peer review only

REFERENCES

1. Shield K, Manthey J, Rylett M, et al. National, regional, and global burdens of disease from 2000 to 2016 attributable to alcohol use: a comparative risk assessment study. *The Lancet Public Health* 2020; **5**(1): e51-e61.
2. Probst C, Parry CD, Wittchen H-U, Rehm J. The socioeconomic profile of alcohol-attributable mortality in South Africa: a modelling study. *BMC medicine* 2018; **16**(1): 97.
3. Matzopoulos R, Walls H, Cook S, London L. South Africa's COVID-19 Alcohol Sales Ban: The Potential for Better Policy-Making. *International Journal of Health Policy and Management* 2020.
4. van Walbeek C, Chelwa G. Using price-based interventions to reduce abusive drinking in the Western Cape Province. 2019.
5. O'Donnell A, Anderson P, Jané-Llopis E, Manthey J, Kaner E, Rehm J. Immediate impact of minimum unit pricing on alcohol purchases in Scotland: controlled interrupted time series analysis for 2015-18. *bmj* 2019; **366**: l5274.
6. Robinson M, Mackay D, Giles L, Lewsey J, Beeston C. Evaluating the Impact of Minimum Unit Pricing (MUP) on Sales-Based Consumption in Scotland: Controlled Interrupted Time Series Analysis. *Public Health Scotland: Edinburgh, Scotland* 2020.
7. McLoughlin J-A, Little F, Mazok C, Parry C, London L. Prevalence of and associations with papsak wine consumption among farm workers in the Western Cape Province, South Africa. *Journal of studies on alcohol and drugs* 2013; **74**(6): 879-88.
8. Republic of South Africa. A review of the taxation of alcoholic beverages in South Africa. In: National Treasury, editor.; 2014.
9. Treasury N. Budget Review 2020. In: Treasury N, editor.; 2020.
10. Ataguba JE-O. Alcohol policy and taxation in South Africa. *Applied health economics and health policy* 2012; **10**(1): 65-76.
11. Jain V, Crosby L, Baker P, Chalkidou K. Distributional equity as a consideration in economic and modelling evaluations of health taxes: A systematic review. *Health Policy* 2020; **124**(9): 919-31.
12. Roberts M, Russell LB, Paltiel AD, Chambers M, McEwan P, Krahn M. Conceptualizing a model: a report of the ISPOR-SMDM modeling good research practices task force–2. *Medical Decision Making* 2012; **32**(5): 678-89.
13. Squires H, Chilcott J, Akehurst R, Burr J, Kelly MP. A Framework for Developing the Structure of Public Health Economic Models. *Value in Health* 2016; **19**(5): 588-601.
14. Howick S, Eden C, Ackermann F, Williams T. Building confidence in models for multiple audiences: The modelling cascade. *European Journal of Operational Research* 2008; **186**(3): 1068-83.
15. Briggs AD, Wolstenholme J, Blakely T, Scarborough P. Choosing an epidemiological model structure for the economic evaluation of non-communicable disease public health interventions. *Population health metrics* 2016; **14**(1): 17.
16. Gunning-Schepers L. The health benefits of prevention: a simulation approach. *Health Policy* 1989; **12**(1-2): 1-255.
17. Rebublic of South Africa. Mid-year population estimates, 2019. In: Statistics South Africa, editor.; 2019.
18. Holmes J, Meier PS, Booth A, Guo Y, Brennan A. The temporal relationship between per capita alcohol consumption and harm: a systematic review of time lag specifications in aggregate time series analyses. *Drug and alcohol dependence* 2012; **123**(1-3): 7-14.
19. Republic of South Africa. CPI History. In: Statistics South Africa, editor.; 2020.
20. Brennan A, Meier P, Purshouse R, et al. The Sheffield alcohol policy model–a mathematical description. *Health economics* 2015; **24**(10): 1368-88.
21. Holmes J, Meng Y, Meier PS, et al. Effects of minimum unit pricing for alcohol on different income and socioeconomic groups: a modelling study. *The Lancet* 2014; **383**(9929): 1655-64.
22. Bradley BD, Jung T, Tandon-Verma A, Khoury B, Chan TC, Cheng Y-L. Operations research in global health: a scoping review with a focus on the themes of health equity and impact. *Health research policy and systems* 2017; **15**(1): 32.

For peer review only

FIGURES

Figure 1: Conceptual model framework

Figure 2: Data inputs for model

Figure 3: Cases averted by condition, split by drinker group and wealth quintile

Figure 4: Comparing the three policy levels: change in mean weekly drinks and cases averted by drinker and wealth group

For peer review only

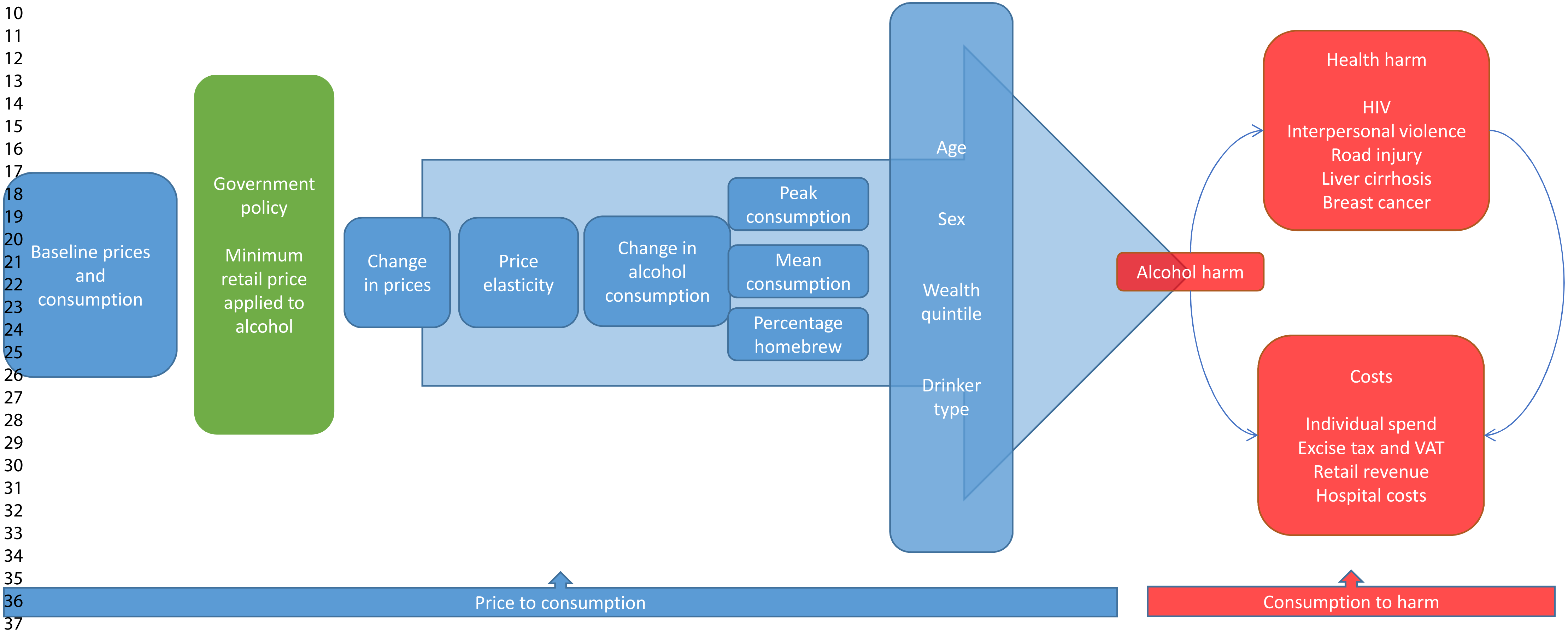
Baseline prices and consumption
Government policy
Minimum retail price applied to alcohol
Change in prices
Price elasticity
Change in alcohol consumption
Peak consumption
Mean consumption
Percentage homebrew
Age
Sex
Wealth quintile
Drinker type
Alcohol harm
Health harm
HIV
Interpersonal violence
Road injury
Liver cirrhosis
Breast cancer
Costs
Individual spend
Excise tax and VAT
Retail revenue
Hospital costs
Price to consumption
Consumption to harm

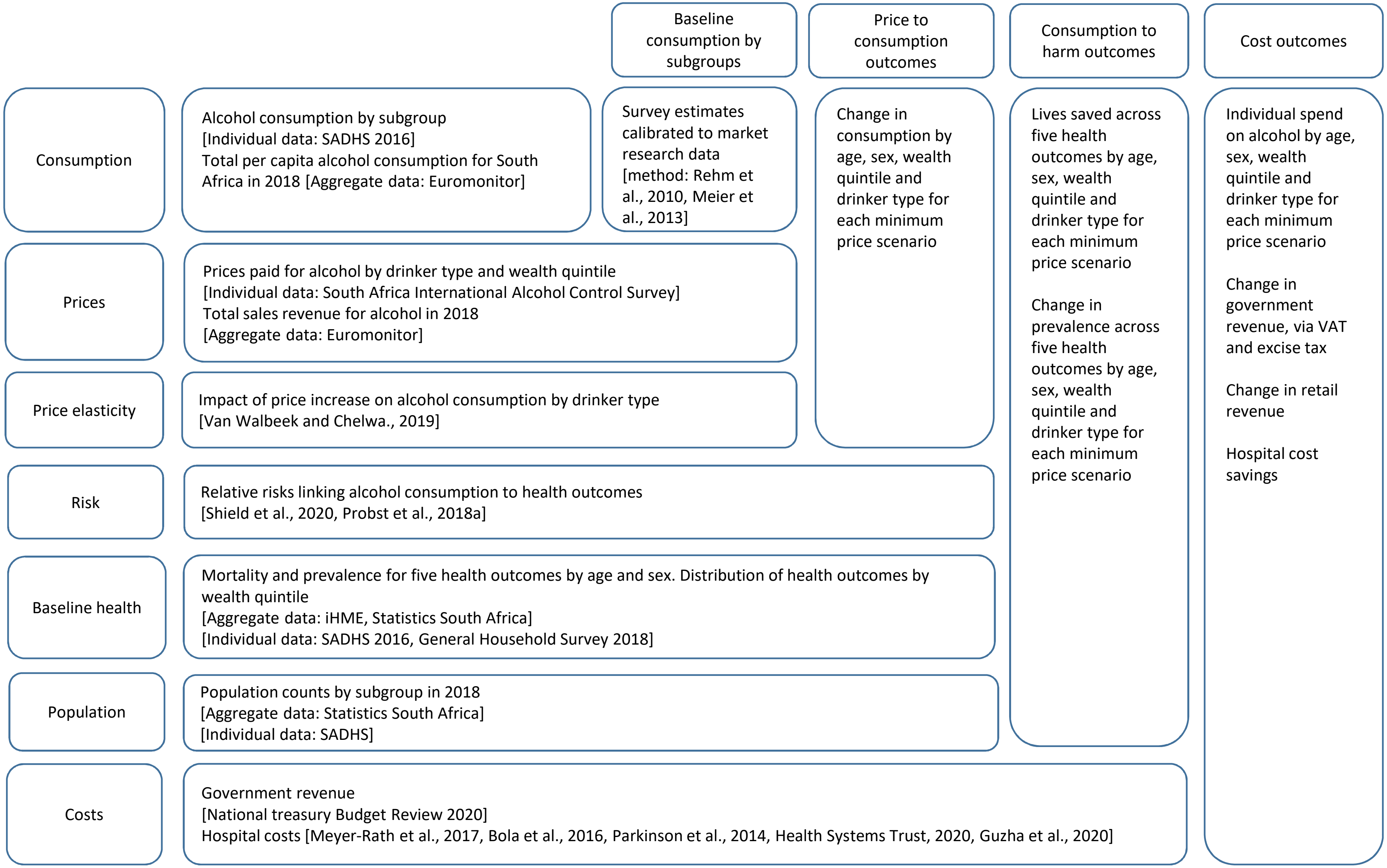
Baseline consumption by subgroups
Price to consumption outcomes
Consumption to harm outcomes
Cost outcomes
Consumption
Alcohol consumption by subgroup [Individual data: SADHS 2016] Total per capita alcohol consumption for South Africa in 2018 [Aggregate data: Euromonitor]
Survey estimates calibrated to market research data [method: Rehm et al., 2010, Meier et al., 2013]
Change in consumption by age, sex, wealth quintile and drinker type for each minimum price scenario
Lives saved across five health outcomes by age, sex, wealth quintile and drinker type for each minimum price scenario
Change in prevalence across five health outcomes by age, sex, wealth quintile and drinker type for each minimum price scenario
Individual spend on alcohol by age, sex, wealth quintile and drinker type for each minimum price scenario
Change in government revenue, via VAT and excise tax
Change in retail revenue
Hospital cost savings
Prices
Prices paid for alcohol by drinker type and wealth quintile [Individual data: South Africa International Alcohol Control Survey] Total sales revenue for alcohol in 2018 [Aggregate data: Euromonitor]
Price elasticity
Impact of price increase on alcohol consumption by drinker type [Van Walbeek and Chelwa., 2019]
Risk
Relative risks linking alcohol consumption to health outcomes [Shield et al., 2020, Probst et al., 2018a]
Baseline health
Mortality and prevalence for five health outcomes by age and sex. Distribution of health outcomes by wealth quintile [Aggregate data: iHME, Statistics South Africa] [Individual data: SADHS 2016, General Household Survey 2018]
Population
Population counts by subgroup in 2018 [Aggregate data: Statistics South Africa] [Individual data: SADHS]
Costs
Government revenue [National treasury Budget Review 2020] Hospital costs [Meyer-Rath et al., 2017, Bola et al., 2016, Parkinson et al., 2014, Health Systems Trust, 2020, Guzha et al., 2020]

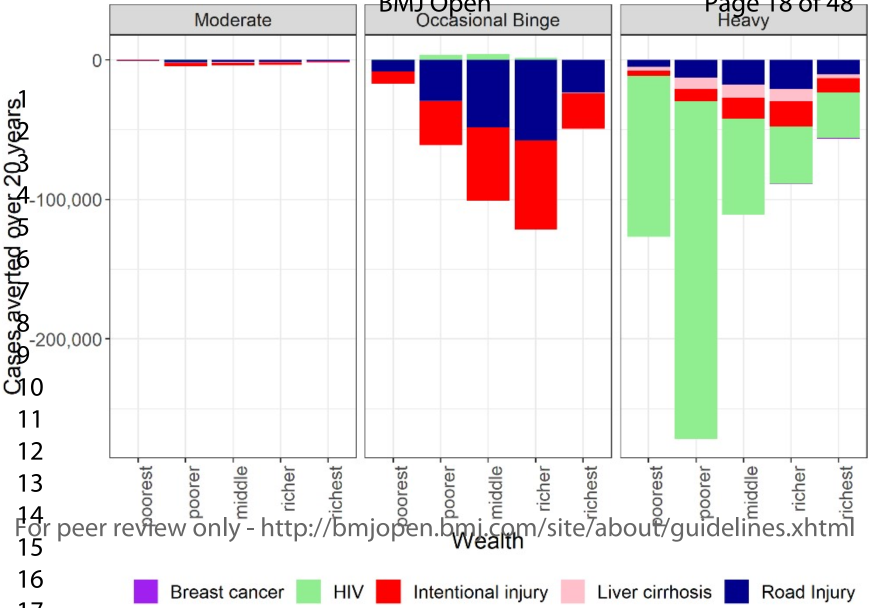

For peer review only - http://bmjopen.bmj.com/site/about/guidelines.xhtml

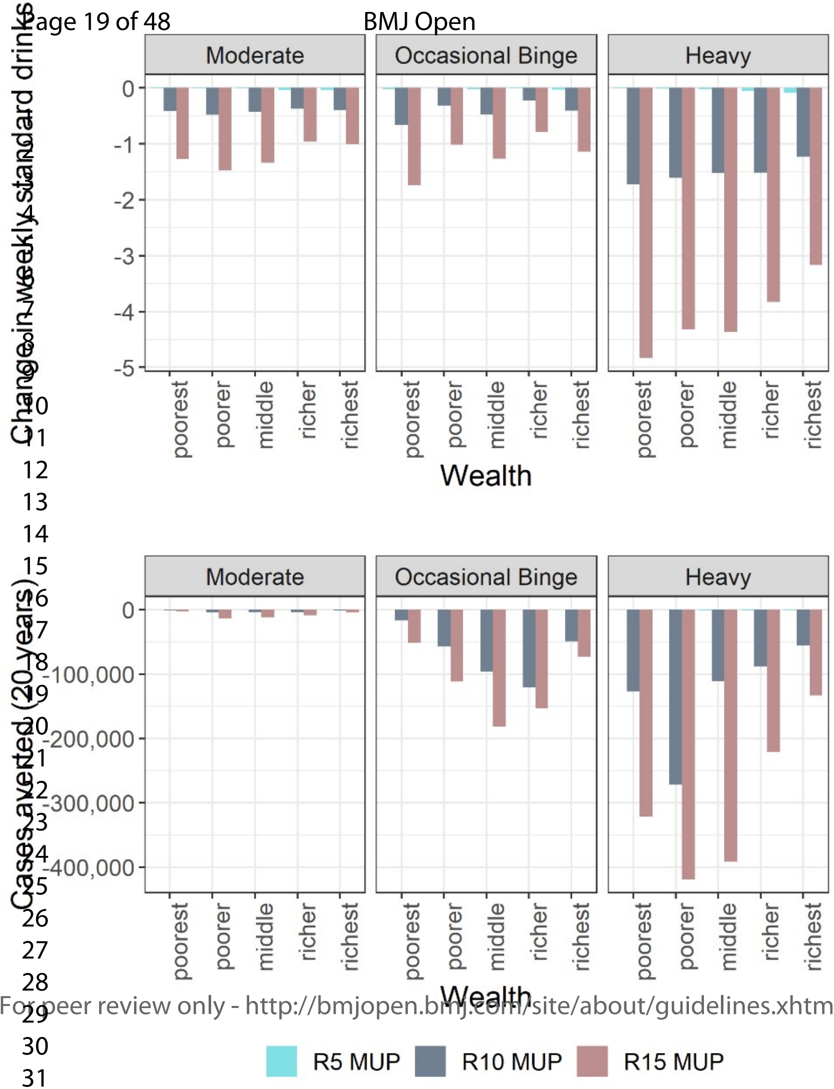
Moderate
Occasional Binge
Heavy
Change in weekly standard drinks
0
-1
-2
-3
-4
-5
poorest
poorer
middle
richer
richest
Wealth
Cases averted (20 years)
-100,000
-200,000
-300,000
-400,000
R5 MUP
R10 MUP
R15 MUP

Contents

Supplementary Material 2

Price to consumption 2

1. Estimating baseline consumption using South African Demographic and Health Survey (SADHS) 2

Process of adjusting the SADHS estimates 2

Process of adjusting peak drinks 3

2. Uplifting consumption 6

3. Uplifting peak consumption 9

4. Wealth quintiles 10

5. International Alcohol Control Study for prices 11

6. Base prices by subgroup 13

Consumption to harm 14

7. Relative risks 14

8. Potential impact fractions 16

9. Socioeconomic gradients of ill health 17

10. Distributing baseline deaths and cases and calculating probabilities 18

11. Baseline health and lagged health impact 20

12. Hospital multipliers and costs 21

13. Sensitivity Analysis 23

References 26

Supplementary Material

Price to consumption

Our model starts by estimating mean and peak alcohol consumption at current alcohol prices at the individual level. The proportion of alcohol consumption which is homebrew is also estimated. This process utilised both alcohol frequency questions and seven day recall questions asked in the same survey. As survey data significantly underreports consumption we calibrate these estimates to market research data using statistical methods established in the literature [1-3]. Following the shift of mean consumption, peak consumption is re-estimated using a simple regression model created at baseline. We categorise drinkers into three exhaustive and mutually exclusive groups; moderate (less than 15 standard drinks per week); occasional binge (less than 15 drinks per week but more than 5 on one occasion); and heavy (15 or more drinks per week). A standard drink in South Africa is currently 15ml or 12 grams of pure ethanol. We compute a regression model for wealth quintiles using the South African Demographic and Health Survey (SADHS) data and use it to predict wealth quintiles in the International Alcohol Control (IAC) dataset to generate price distributions for wealth and drinker groups. Alcohol is treated as one commodity due to data constraints.

1. Estimating baseline consumption using South African Demographic and Health Survey (SADHS)

The SADHS survey asked the following questions:

Survey	Alcohol questions [answers]
SADHS 2016	Have you ever consumed a drink that contains alcohol such as beer, wine, ciders, spirits, or sorghum beer?<br>Probe: Even one drink?<br>[yes, no]<br><br>Was this within the last 12 months?<br>[yes, no]<br><br>In the last 12 months, how frequently have you had at least one drink?<br>[5 or more days a week, 1-4 days per week, 1-3 days a month, less often than once a month]<br><br>During each of the last 7 days, how many standard drinks did you have?<br>[use showcard, record total number of drinks consumed each day starting with the day before the day of the interview and proceeding backwards]<br><br>During the last 7 days, how many standard home-made beers or other homemade alcohol did you have?<br>[use showcard, record number]<br><br>In the past 30 days, have you consumed five or more standard drinks on at least one occasion?<br>[yes, no]

Table 1: Survey questions

Process of adjusting the SADHS estimates

Drinkers were categorised by their drinking frequency and by whether or not they had reported any drinking in the last seven days

For peer review only

Drinking occasion frequency	count	Reported drinks in last 7 days	Reported zero drinks in last 7 days
5 or more days a week	293	266	27 (6 binge, 21 drinker)
1-4 days per week	668	565	103 (29 binge, 74 drinker)
1-3 days per month	1163	799	364 (all drinkers)
less often than once a month	1187	404	783 (all drinkers)
NA	7025		

Table 2: Frequency of alcohol consumption responses

Firstly readjusting those with a seven day drinking pattern (pink numbers)
The pink numbers are respondents who say they only drink 1 -3 days per month or less often than once a month but they have drank in the last 7 days. If this were multiplied by 52 it would be an overestimate.

Drink frequency 1 – 3 days per month
Assume for those that drink 1-3 days per month we have caught their one drinking week in the month and multiply by 12 to get their annual consumption. There are 799 people in this category.

Drink frequency less than once per month
Assume for those who drink less often than once a month but who did drink in the last week we have caught their one drinking week in the month. Multiply by 6, to get the annual figure. There are 404 people in this category.

The yellow numbers are ok as respondents report drinking every week and have a seven day drinking pattern.

Readjusting those without a seven day drinking pattern but who say they drink (blue and red numbers)
Drink frequency 5 or more days per week
Use the mean standard drinks for drinkers (and binge drinkers) who reported the same frequency but who do have a seven day pattern, there are 266 people (yellow). This is computed separately for sex and drinker group.
Drink frequency 1 - 4 days per week
Use the mean standard drinks for drinkers (and binge drinkers) who report the same frequency but who do have a seven day pattern, there are 565 people (yellow). This is computed separately for sex and drinker group.
Drink frequency 1 – 3 days per month
Use the mean adjusted annual drinks (adjusted above) of the equivalent frequency group who did report a drinking pattern, said no to drinking 5 or more, split by sex. This uses observations from 799 people (pink).
Drink frequency less than once per month
Use the mean adjusted annual drinks (adjusted above) of the equivalent frequency group who did report a drinking pattern, said no to drinking 5 or more, and split by sex, This uses observations from 404 people (pink).

Alternatively those who drink less than once a month, assume they drink 2.5 standard drinks (midpoint of 1 – 4) 6 times a year. This would mean they drink 2.5 drinks * 6 months = 15 standard drinks, again a similar ball park.

Process of adjusting peak drinks
Using the same process as above I also applied a peak drink to those observations which did not have one. However before this I checked all those who reported binge drinking had a peak drink at minimum of 5, which they did.

Comparing the adjusted SADHS data with the estimates using only 7 day recall we see that as expected prevalence of drinking increases and per capita estimates reduce (Table 3).

	Prevalence of drinking		Sample size: drinkers	Annual litres of alcohol - per capita			Annual litres of alcohol - Just drinkers		
	Female	Male		Total	Female	Male	Total	Female	Male
**SADHS (7 day recall only)**	9.3%	32.7%	n = 1949 (report drinks	2.2	0.65	4.5	10.6	6.54	12.2

For peer review only

**Population weights applied**			in the 7 day recall) females = 571 males = 1378						
**SADHS adjusted (7 day recall plus adjustments based on frequency questions) Population weights applied**	18%	54%	n = 3311 females = 1125 males = 2186	1.65	0.50	3.4	5.0	2.59	6.25

Table 3: Comparing adjusted with unadjusted statistics

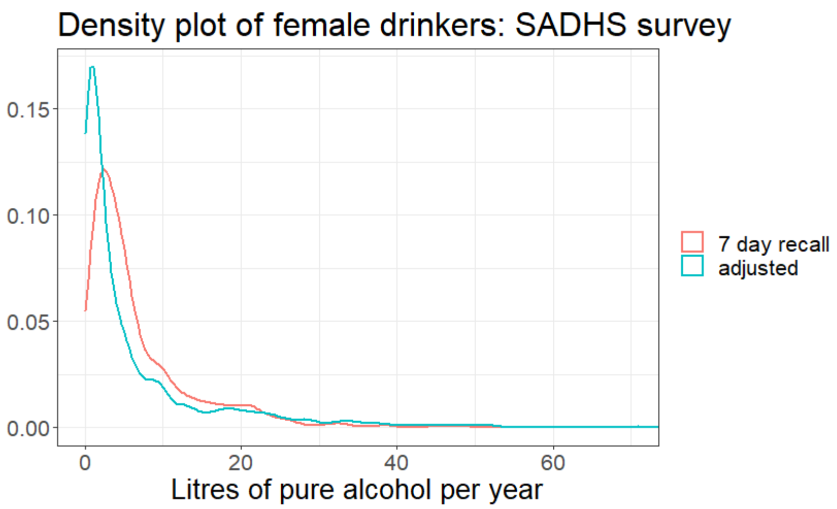

Figure 1: Density plot of female drinkers before and after the shift

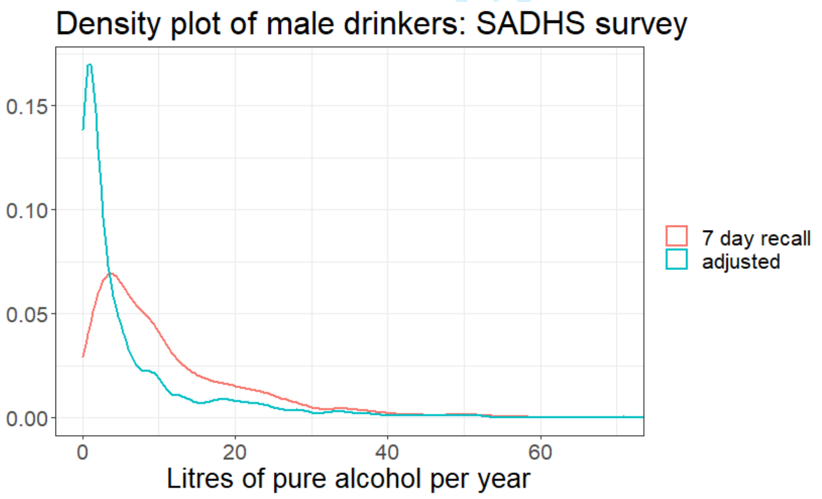

Figure 2: Density plot of male drinkers before and after the shift

Incorporating the frequency data into the seven day recall moves the distribution towards the left (Figures 1 and 2). This is logical as the sample will now include those drinkers who stated that they drink but did not record any for the last seven days, it also adjusted down those who claim to drink less than weekly but who did recall

drinks for the last seven days. This pattern gives some confidence in the dataset and utilises the strengths of capturing heavy drinking well and including occasional drinkers.

For peer review only

2. Uplifting consumption

Surveys provide important data about drinking patterns within the population but, as previously mentioned, they provide total consumption estimates that are far smaller than that indicated by government sources or market research data [4]. There are established ways of adjusting alcohol consumption survey data to account for this via statistical calibration techniques [2,3]. The following steps outline how I tailored the methods to the South Africa context:

- First a cap was applied to all drinkers of 68 litres of alcohol per year or 150 grams of alcohol per day. As the model includes long term effects (20 years) the cap is needed as a higher level of alcohol cannot be sustained in the long term [5]. This cap impacted one woman and ten men. Of this small group only two men drunk both homebrew and recorded alcohol and so their total consumption was reduced to 68 litres and then split into recorded and homebrew using their previous percentage split.

- Survey coverage level was calculated as the difference between total per capita consumption recorded in the SADHS survey and per capita consumption using Euromonitor recorded sales data for 2018. 80% of the sales data is used to account for spillage, stockpiling and tourist consumption. This sales figure was then increased to take account of the 4.15% of total alcohol consumed in the survey reported as homebrew (representing unrecorded alcohol in the model). The comparison of total consumption according to the survey and the adjusted official sales data was used to create the multiplication factor.

- For female and male subgroups the mean litres of alcohol was adjusted by the multiplication factor.

- This new mean and the relationship between mean and standard deviation, established in the literature [2], was used to compute a standard deviation and then fit a "shifted" gamma distribution (maintaining the cap of 68 litres) using the mean and standard deviation, calculated for male and females separately.

$$\hat{\sigma}_{shifted} = 1.174 \times \hat{\mu}_{shifted} + 1.003 \times sex$$

- It is possible to stop at this point; however, there are two limitations with this method. Firstly, there is no empirical evidence that under coverage is distributed as implied by the shifts needed to fit the adjusted consumption to the gamma. Secondly, that shifting consumption to a gamma can artificially reduce the long tail of heavy drinkers [3].
- Therefore, a gamma distribution was fitted to the original sample of drinkers by sex and percentiles were taken across both distributions. Percentage differences in consumption were calculated at each percentile. These increases were then applied to the percentiles of the original survey sample
- Each individuals total consumption was split into homebrew and recorded alcohol using the original percentage split (this assumes underreporting is equal across homebrew and recorded alcohol)
- Results were compared (Table 4 and Figures 3 and 4). There is a fairly small difference between the two methods, more visible for males than females. It appears adjusting by percentiles only makes a difference at the extremes, lowering the left hand peak slightly but also falling below the Gamma shifted distribution after 60litres of alcohol per year meaning there is a smaller number of the very high drinkers. The percentile adjusted distribution was used for the main model.

Females – litres of alcohol per year	Mean	Min	Max
**SADHS Survey data (weighted mean and capped)**	2.57	0.09	68
**Gamma fitted to survey (difference due to weights)**	2.50	0.09	68
**Gamma shift**	10.78	1	68
**Adjusting each percentile (weighted mean)**	10.74	0.5	68
Males – litres of alcohol per year			
**SADHS Survey data (weighted mean and capped)**	6.13	0.09	68
**Gamma fitted to survey (difference due to weights)**	5.6	0.09	68
**Gamma distribution shifted**	18.55	1	68
**Adjusting each percentile (weighted mean)**	19.2	0.5	68

Table 4: Comparing pre and post shift data

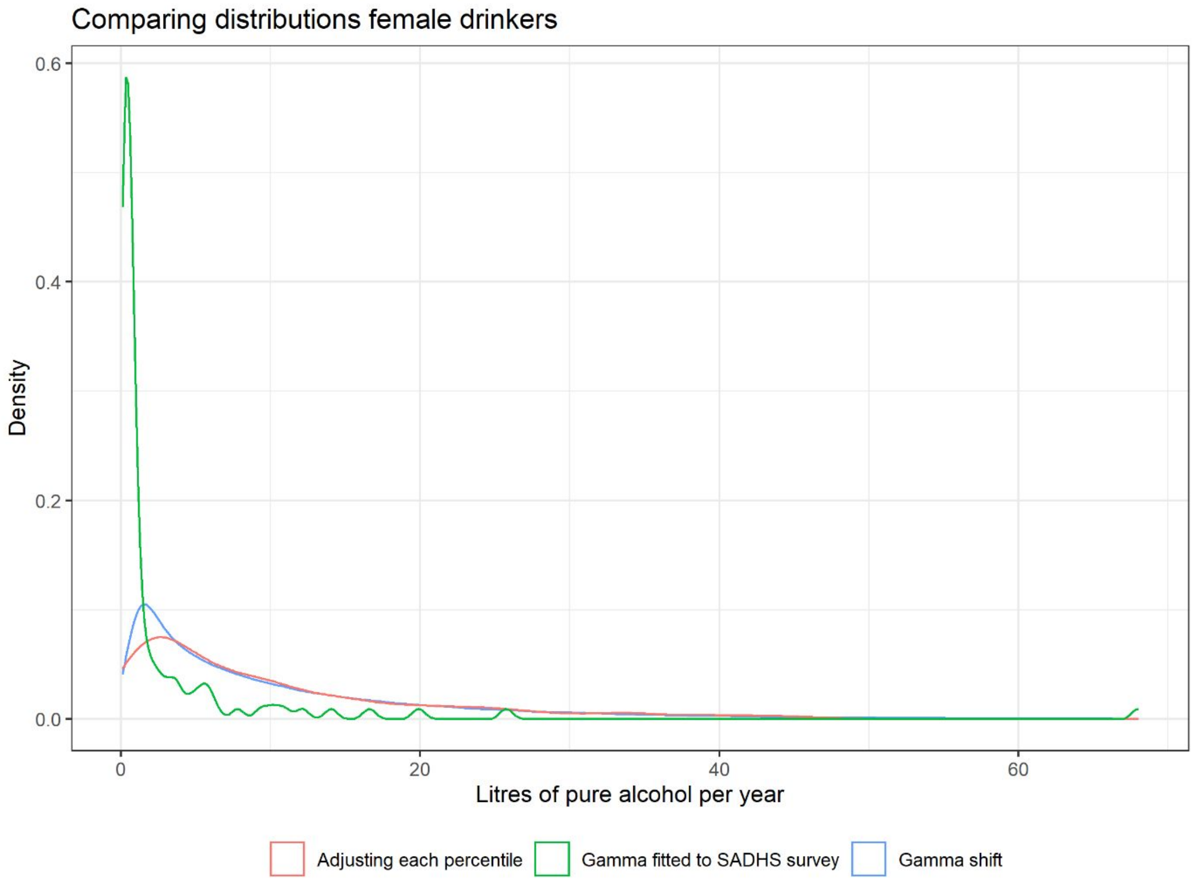

Figure 3: Comparing distributions pre and post shift females

review only

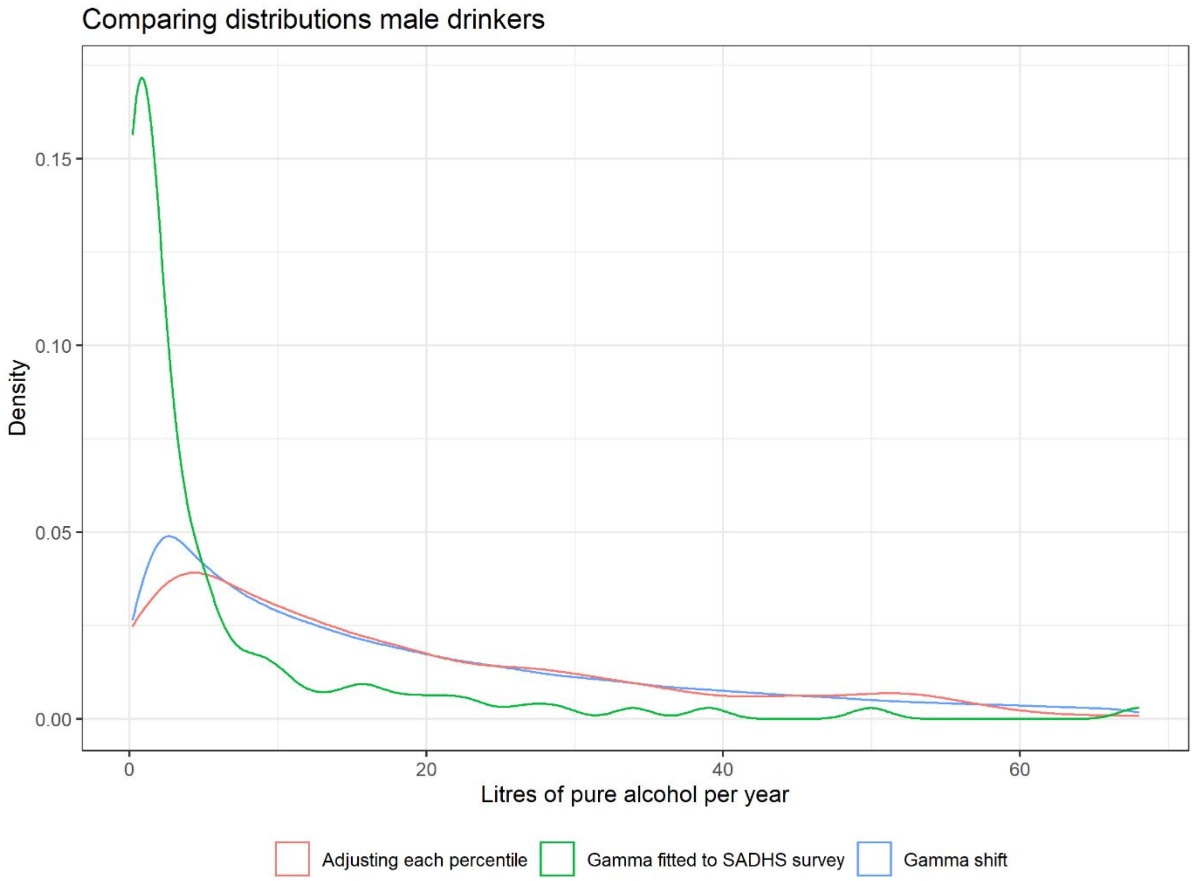

Figure 4: Comparing distributions pre and post shift males

3. Uplifting peak consumption

Peak drinking is important as it will influence health harms directly. Following the method used in the Sheffield Alcohol Policy Model [6], the following linear regression model was fitted, for all drinkers, to the non-shifted SADHS data, relating peak drinking to mean consumption, age and sex.

```
peak_model <- lm(dk_peak_SADHS ~ lit_SADHS + age_band_4 + sex)
```

The model was used to compute fitted values for the non-shifted data. The model assumes there is a linear relationship between peak and mean consumption, the magnitude of which is allowed to vary by age and sex.

After the mean consumption was shifted as above the corresponding new peak consumption was computed using the following formula:

$$peak_{ij}\text{ (shifted)} = peak_{ij}\text{ (SADHS)} \times (E(peak_{ij}\text{ (shifted)})/E(peak_{ij}\text{ (SADHS)}))$$

The linear relationship between mean and peak discovered in the SADHS survey is maintained for the shifted mean and peak consumption which assumes individuals under reported peak and mean consumption by the same magnitude. The prediction error for the model is of the same magnitude for all levels of consumption.

The predictions were checked to ensure that peak drinking was never predicted below mean daily drinking. There were 88 people (out of the 3311 drinkers) for whom this was true. These people had their peak drinking increased to match their mean daily drinking.

For peer review only

4. Wealth quintiles

In order to match wealth groups between the two datasets an ordered choice model was created using SADHS data with wealth quintile (1 – 5) as the dependent variable, using the MASS package in R [7]. All the variables that were common across the two datasets were included in the initial model, these were not just asset ownership but also age, sex , educational level and population group (race). Stepwise regression was performed using the step.AIC function. This chooses the best variables to include by running the regression with all variables in and then taking one out and computing a goodness of fit measure (the AIC). If the goodness of fit measure is improved then that model is preferred, it runs this for many models until it finds the model with the highest AIC. This method resulted in the selection of the following variables: age, sex, population group, education level, car, landline, electricity, fridge, computer, radio, tv. The only variable it removed was mobile phone which fitted anecdotally with conversations I had with stakeholders in South Africa regarding how much poorer people prioritise mobile phones.

polr(formula = wealth ~ car + electricity + tv + fridge + radio + telephone + computer + age + edu + pop_group, data = SADHS, Hess = TRUE)

The goodness of fit matrix evaluates the success of the model, comparing the closeness of the predicted and observed outcome (Table 5). The model never predicts the poorest as the richest or the richest as the poorest.

	**Prediction**					
		Poorest	Poorer	Middle	Richer	Richest
**Actual**	Poorest	1300	593	196	9	0
	Poorer	299	975	744	192	17
	Middle	62	612	1042	595	26
	Richer	5	236	763	818	244
	Richest	0	10	108	422	1068

Table 5: Goodness of fit matrix

5. International Alcohol Control Study for prices

The IAC dataset provides prices by drinking location by beverage and also asks whether the individual binge drinks, demographic data is also collected. The survey asked for the price in Rands by location, for example they ask for the price of a beer paid at a pub for each container size. There are 17 drinking locations (12 on trade and 5 off trade) and 12 drink types.

Prices were disaggregated by population subgroups rather than by drink type (wine/beer/spirits etc). This was consistent with the South Africa specific price elasticities which were calculated for drinker groups whilst treating alcohol as a single commodity. The IAC respondents were categorised into drinker groups using the definitions above. Each price was weighted by the number of units (e.g. bottles, glasses, cans) sold, the container size of those units and the number of drinking occasions in 6 months (Figure 5). Every price observation was validated using data from the South African Consumer Price Index. Prices were increased to 2018 to account for inflation.

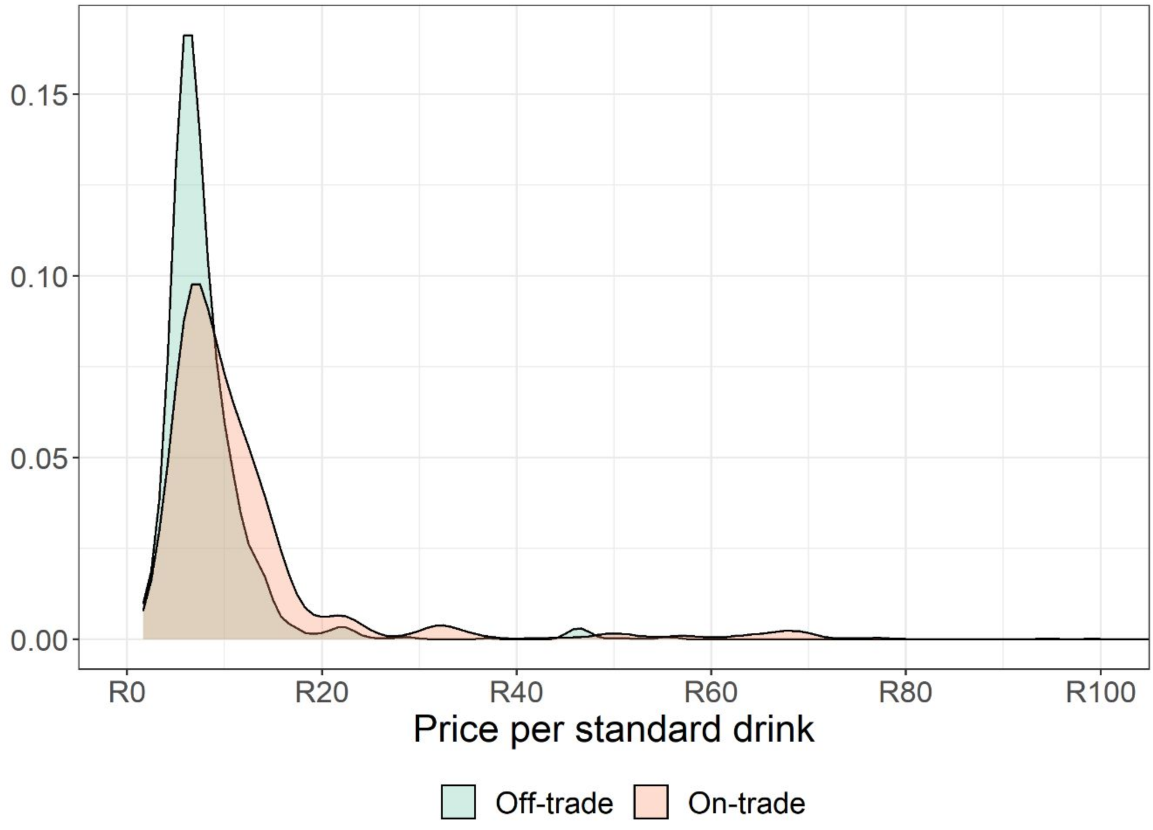

Figure 5: Distribution of off-trade and on-trade prices

The off-trade wine prices were adjusted using data from the South Africa Wine Industry Statistics [8] who report the proportions of still wine sold (which makes up 93% of total volume of wine sold) in the off-trade in 2018 that falls within different price bands, this data was used to adjust downwards the off-trade wine (Table 6). The price observations were sorted in ascending order and a cumulative volume variable created. The price closest to the 49th percentile was then adjusted down to R3.74 and all prices below adjusted using the same proportion. The prices at the very bottom were adjusted so they could not go below R2.50. The same adjustment process was applied to each of the four groups.

Retail price per litre of wine for 2018	Price per standard drink (15ml) assuming 12% abv	Cumulative percentage of total still wine sold	Cumulative percentage of IAC data for	Cumulative percentage of IAC data for

		at price SAWIS data	off-trade wine pre-adjustment	off-trade wine post-adjustment
**Less than R30**	Less than R3.75	49%	33%	51%
**> R30 – R48**	> R3.75 – R6	82%	60%	83%
**> R48 - R72**	> R6 – R9	89%	77%	89%
**> R72 - 108**	> R9 – R13.5	95%	89%	95%
**> R108**	> R13.5	100%	100%	100%

Table 6: Price distribution for off-trade wine

As the Tschwane prices were collected in one locality, they were validated against national data sources. Beer is by far the most popular drink, accounting for over 50% of the alcohol sold so beer prices are critical. I accessed data from the South Africa Consumer Price Index for January 2020 to compare the Gauteng province (where Tshwane is located) with other provinces. Beer, which accounts for over 50% of alcohol sold in South Africa, Guateng is at R13.76 for a 330ml can. The average across the eight prices listed above is R13.66 which is very close to Guateng's price, therefore we assume the same price distributions across the whole of South Africa.

Finally, prices were validated with all stakeholders including individuals resident in townships who could provide anecdotal evidence relating to cheap alcohol available at shebeens.

For peer review only

6. Base prices by subgroup

All IAC drinkers were now categorised by drinker type and by wealth quintile (Table 7). Wealth quintile was predicted using the ordered choice model created using the SADHS data. Drinkers in the lowest wealth quintile appear the least likely to drink in moderation leaving a very small sample size (this is not weighted by number of drinks). It is therefore not possible to create price distributions for all 15 categories.

	Moderate obs (individuals)	Occasional Binge obs (individuals)	Heavy obs (individuals)
**Poorest**	2 (2)	29 (23)	35 (24)
**Poorer**	8 (8)	23 (18)	28 (20)
**Middle**	11 (11)	132 (90)	88 (40)
**Richer**	23 (20)	95 (59)	60 (30)
**Richest**	93 (68)	135 (93)	101 (50)

Table 7: Count of IAC price observations and respondents within each category

The mean price for each of these drinker categories demonstrates there is wealth gradient (Table 8).

	Moderate	Occasional Binge	Heavy
**Poorest**	R6.79	R7.97	R7.78
**Poorer**	R9.43	R10.0	R9.65
**Middle**	R10.2	R10.1	R9.23
**Richer**	R11.3	R13.4	R10.6
**Richest**	R11.7	R11.1	R12.8

Table 8 Mean price of standard alcoholic drink (15ml of pure alcohol) for each subgroup

In order to ensure adequate sample size the poorest/poorer/middle and richer/richest categories were aggregated for moderate drinkers (Table 9).

	Moderate	Occasional Binge	Heavy
**Poorest**	R9.13	R7.97	R7.78
**Poorer**	R9.13	R10.0	R9.65
**Middle**	R9.13	R10.1	R9.23
**Richer**	R11.6	R13.4	R10.6
**Richest**	R11.6	R11.1	R12.8

Table 9 Mean price of standard alcoholic drink (15ml of pure alcohol) within each subgroup

For peer review only

Consumption to harm

7. Relative risks

Relative risks were calculated for each of the health outcomes of interest at baseline, and each policy scenario using published relative risk equations [9,10]. The same relative risk equations are used for morbidity (or prevalence) and mortality. HIV risk is derived from a stepped function for mean drinking, intentional injuries and road injury from a continuous function of mean drinking differing by whether the individual binge drinks, liver cirrhosis and breast cancer from a continuous function of mean drinking, for breast cancer this is only for females (Table 10).

**Health Condition**	**Relative risk Current drinkers**	**Relative risk former drinkers**	**ICD-10 codes**
**HIV**	Low SES<br>$RR = 2.99$<br>if x > 61/49 grams per day (males/females)<br>$RR = 1.94$ if x > 0<br>$RR = 1$ otherwise<br><br>Higher SES<br>$RR = 1.54$<br>if x > 61/49 grams per day (males/females)<br>$RR = 1$ otherwise	RR = 1	B20-24
**Intentional Injuries**<br><br>**(self-harm and interpersonal violence)**	Drinkers<br>$RR = \exp(00199800266267306 . x)$<br><br>Heavy episodic drinkers (HED)<br>$RR =$<br>$\exp(00199800266267306 . x + 0.647103242058538)$	RR = 1	ICD-10 codes: X60 – Y09<br>Y35 –36<br>Y870<br>Y871
**Road Injury**<br><br>**(pedestrian, cyclist, motorcyclist, motor vehicle, other road)**	Drinkers<br>$RR = \exp(0.00299550897979837 . x)$<br><br>Heavy episodic drinking<br>$RR =$<br>$\exp(0.00299550897979837 . x + 0.95935022133460$	RR = 1	V01–04,<br>V06,<br>V09–80,<br>V87,<br>V89,<br>V99
**Breast Cancer**	Females only<br>$RR = \exp(0.01018 . x)$	RR = 1	C50
**Liver**	if x <= 1<br>$1 + x.\exp((\beta_1 + \beta_2).\sqrt{\frac{1 + 0.1699981689453125}{100}})$<br><br>If x > 1<br>$\exp((\beta_1 + \beta_2).\sqrt{\frac{x + 0.1699981689453125}{100}})$<br><br>Female<br>b1 = 2.351821<br>b2 = 0.9002139	RR = 3.26 for both females and males	K70, K74

For peer review only

	Male<br>b1 = 1.687111<br>b2 = 1.106413		
x = grams of alcohol consumed per day among current drinkers<br>HED = drinking 60 grams or more on one drinking occasion			

Table 10: Relative risk equations used

For peer review only

8. Potential impact fractions

Potential impact fractions (PIFs) were calculated by dividing relative risk under each policy by relative risk at baseline. These incorporated population weights and were computed by sex *(i)*, wealth group *(j)* and drinker group *(k)*.

$$PIF_{ijk} = \frac{\text{relative risk}_{ijk}\ (\text{policy})}{\text{relative risk}_{ijk}\ (\text{baseline})}$$

For peer review only

9. Socioeconomic gradients of ill health

Health outcomes in South Africa are not evenly distributed throughout the population, with the poor often bearing a higher burden of disease, depending on the illness. Data analysis was carried out using General Household Survey (GHS) data for 2018. The ordered choice regression model computed previously, using SADHS data, was applied to the GHS data to split the survey population into wealth quintiles compatible with the foundational dataset (SADHS). Percentage within each wealth quintile with the disease was computed (Table 11). Liver cirrhosis was not one of the health conditions included in the survey and breast cancer was not specifically included although the broader category of cancer was. Sensitivity analysis was carried out using alternative gradients.

	**poorest**	**poorer**	**middle**	**richer**	**richest**
**15+ raw count** (648 NAs)	4966	11462	14396	9633	7630
**HIV** raw count percentage	395<br>0.08	684<br>0.06	614<br>0.04	155<br>0.02	41<br>0.005
**Intentional injuries*** raw count percentage	11<br>0.002	30<br>0.0027	24<br>0.0018	11<br>0.0012	3<br>0.0002
**Road injuries**** raw count percentage	7<br>0.0016	26<br>0.0022	22<br>0.0016	32<br>0.0033	13<br>0.00015
**Cancer** raw count percentage	2<br>0.00038	27<br>0.0012	41<br>0.0026	27<br>0.0029	68<br>0.008

nb: percentages within each quintile were calculated incorporating the survey weights

* gunshot wounds; severe trauma due to violence, assault, beating; intentional poisoning; accidental poisoning; fire and burn; crime related injury – left out sports related, disability related and other
** motor vehicle -occupant, motor vehicle – pedestrian, bicycle related

Table 11: Raw count of General Household Survey data 2018

For peer review only

10. Distributing baseline deaths and cases and calculating probabilities

The deaths/cases (which come disaggregated by sex) at baseline is split between the five wealth quintiles using the GHS data to account for the socioeconomic gradient, as explained above. However, a preparatory step was necessary as the proportions of the population (using the SADHS proportions) in each quintile were not perfectly equal, for example for Q1, Q2, Q3, Q4, Q5 corresponded to 0.19, 0.19, 0.20, 0.21, 0.21 for females and 0.19, 0.20, 0.21, 0.20, 0.21 for males. The probability of death was calculated for each quintile first by assuming the population was split into quintiles of equal size. The total deaths/cases for each quintile using the SADHS proportions was then calculated by applying the relevant probability of death/cases for that part of the quintile which overlapped with the underlying equally sized quintile. This concept can be best illustrated on a graph.

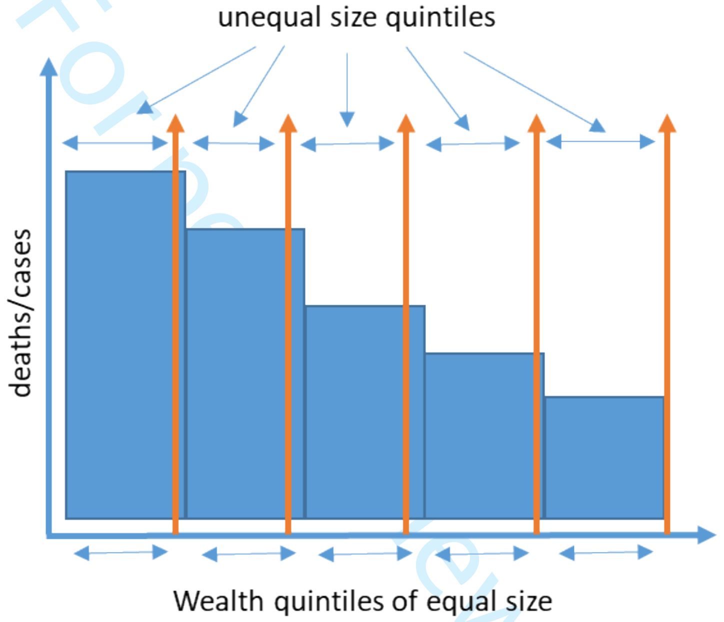

$$Number_{cases}(SADHS_{Q1}) = Pop_{SADHSQ1} \times Prob_{EqualQ1}$$

$$Number_{cases}(SADHS_{Q2}) = (Pop_{EqualQ1} - Pop_{SADHSQ1}) \times Prob_{EqualQ1} + (Pop_{SADHSQ1} + Pop_{SADHSQ2} - Pop_{EqualQ1}) \times Prob_{EqualQ2}$$

$$....and\ so\ on$$

The existence of relative risk equations implies that the baseline mortality/morbidity will also not be distributed equally between drinker groups, one would expect a higher proportion of the baseline cases to exist amongst heavy drinkers, followed by occasional binge, moderate then abstainers. In order for the baseline mortality/morbidity to vary by drinker group the total risk, for each disease, is calculated for each drinker group group, by sex and wealth quintile. The proportional share of risk between drinker groups is then calculated and used to distribute the mortality/morbidity, which has already been assigned to each quintile, between each drinker group within that quintile.

The model uses iHME data for deaths and cases of disease and population statistics (Statistics South Africa) from 2018. Life tables to get the probability of death by single year of age were only available for 2017 from iHME so these were used. The 2018 population is split proportionally into the sex/wealth/drinker groups using the SADHS proportions.

The probability of death for each disease is calculated for the baseline scenario and taken away from overall probability of death for each single year of age given in the life table to give a probability of

For peer review only

death from non-modelled causes. This probability of death from non-modelled causes remains constant at every policy scenario. The probability of death from the five diseases of interest then vary according to the policy level and the corresponding potential impact fraction.

We model counterfactual population structure (i.e. in the absence of the policy) over 20 years, starting from 2018 using current population estimates from Statistics South Africa, plus birth projections for 2020 to 2023 and assume current age-, sex- and wealth-specific mortality rates remain constant [11]. Birth cohorts for years beyond 2023 are not modelled as they would not have reached the age at which we model alcohol consumption (15+) within the time horizon.

We create multistate life tables in which the population faces a probability of mortality for each of the five disease/injury conditions and for other cause mortality each year. This approach allows us to simulate prevalence of and mortality from multiple diseases simultaneously, assuming diseases are independent of one another. The model generates alternative population impact fractions (as above) for baseline and for each policy scenario. Using the relevant population impact fraction and rerunning the multistate life table enables a calculation of the difference between baseline and the policy.

For peer review only

11. Baseline health and lagged health impact

HIV, road injuries and intentional injuries realise the full impact of the reduction in drinking immediately whereas the health impact on liver cirrhosis and breast cancer are subject to lags in the effect, meaning the reduced drinking does not translate to a reduced health risk immediately [12]. Breast cancer starts to see an impact at year 11 and it is 20 years until full effect, liver cirrhosis sees some impact from year one but does not realise the full effect until year 20 (Appendix part 9).

The life tables for the 20 year time horizon are saved for each of the policy scenarios. They are then used in combination with the probability of having the disease and the potential impact fraction under each policy, to estimate the number of cases.

HIV, road injuries and intentional injuries realise the full impact of the reduction in drinking from the first year of the drinking reduction whereas liver cirrhosis and breast cancer are subject to lags in the effect. Breast cancer only starts to see an impact at year 11 and it is 20 years until full effect, liver cirrhosis sees some impact from year one but does not realise the full effect until year 20 (Table 12).

**Year**	**1**	**2**	**3**	**4**	**5**	**6**	**7**	**8**	**9**	**10**	**11**	**12**	**13**	**14**	**15**	**16**	**17**	**18**	**19**	**20**
**Breast cancer**	0	0	0	0	0	0	0	0	0	0	10	20	30	40	50	60	70	80	90	100
**Liver Cirrhosis**	21	34	43	50	56	61	65	69	73	76	79	82	85	88	90	92	94	96	98	100

Table 12: Modelled time-lags by condition – proportion of overall change in risk experienced in each year following a change in consumption (Holmes et al., 2012)

For peer review only

12. Hospital multipliers and costs

The prevalence of disease/injury at each policy scenario for each year of the 20 year time horizon was multiplied by the proportion who would then go on to receive hospital treatment (Table 13) and the relevant hospital cost applied (Table 14). The costs taken from the literature were increased by inflation where necessary to reach the baseline year of 2018. Future costs were discounted at 5% as recommended by the Department of Health in the guidelines for pharmacoeconomic submissions [13].

Condition	Multiplier (cases in population who go on to receive healthcare treatment)	Source
**HIV**	0.62	UNAIDS estimates that 62% of people living with HIV in 2018 in South Africa were on treatment [14]
**Intentional Injury**	0.41	Survey estimating trauma admissions [15] combined with iHME data from the same year to predict multipliers.
**Road injury**	0.19	Survey estimating trauma admissions [15] combined with iHME data from the same year to predict multipliers.
**Liver Cirrhosis**	0.5	Paper on liver cirrhosis in sub-Saharan Africa suggests 50% of patients are admitted to hospital with end-stage liver disease [16].
**Breast Cancer**	0.75	All studies found estimate what proportion present with late stage breast cancer (51%) but not what proportion never receive hospital treatment [17]. Therefore an estimate of 0.75 is used.

Table 13: Estimated multiplier from population prevalence to hospital admission

Condition	Cost per patient	Source
**HIV**	R 3,318.62 (2017/18)	This is the annual cost. Taken from a systematic literature review of per patient costs of HIV services in South Africa [18]. There are many different levels of treatment, this cost is only for first-line treatment, so this is conservative.
**Intentional Injury**	R58,928 (2013)	This retrospective case note review included 143 violence related emergency hospital admissions from January to March 2013. Average inpatient stay was 9.8 days with treatments including emergency surgery, intensive care and resuscitation beds on admission [19].
**Road injury**	R56,592.17 (2012)	A prospective cohort study followed 100 patients admitted following a Road traffic injury between late 2011 and early 2012 at Edendale Hospital Pietermartizburg [20].
**Liver Cirrhosis**	R2,967 (2018)	50% multiplier used above comes from paper suggesting 50% of liver cirrhosis patients get admitted to hospital with end stage liver disease. Treatment for end stage liver disease includes<br><br>A specific study on liver cirrhosis was not found so general costs have been used from the district health baromenter. Expenditure per patient day equivalent (district hospitals) was R2967 (average taken from across the 9 provinces). This assumes just one patient day. Conservative. [21]

For peer review only

**Breast Cancer**	Early stage R14,915<br>Late stage R16,869<br>(2015)	This retrospective case review included 200 women at a government hospital in South Africa. The average cost is different depending on whether they were diagnosed at an early (56%) or late (44%) stage [22].

Table 14: Hospital costs and sources

For peer review only

13. Sensitivity Analysis

Parameter	Central estimate	Alternative plausible values	Rationale	Results
Price elasticities	-0.40 moderate<br>-0.22 occasional binge<br>-0.18 heavy	Scenario 1<br>-0.5 for high income drinkers (applied to quintiles 3, 4, 5)<br><br>-0.86 for low income drinkers (applied to quintiles 1,2 to be conservative)<br><br>Scenario 2<br>-0.8	Scenario 1<br>Estimates using NIDs data for two subsets of the population, the top 50% and bottom 50% of households by total household expenditure [23].<br><br>Scenario 2<br>Van Walbeek and Blecher [23] literature review of South African specific price elasticities found Selvanathan and Selvanathan [24] estimated -0.8 which corresponds closely to price elasticity estimates for beer (-0.8), wine (-0.9) and spirits (-0.9) produced by SALBA (2010).	Central estimates<br>Consumption - 4.50%<br>Spend + 17.83 %<br>Lives saved 23,011<br>Cases saved 1,010,354<br><br>Scenario 1<br>Consumption - 14.15%<br>Spend + 5.43%<br>Lives saved 55,638<br>Cases saved 2,468,299<br><br>Scenario 2<br>Consumption - 17.95<br>Spend + 0.10%<br>Lives saved 64,929<br>Cases saved 2,934,690
Proportion of abstainers in the population	82% female non-drinkers<br>45% male non-drinkers	67% female non-drinkers<br>36% male non-drinkers	Stakeholders have indicated scepticism about the prevalence of non-drinking reported in SADHS (and all alcohol studies). Currently the model only adjusts the consumption of those who report anything at all. We will increase the survey weightings of drinkers in the SADHS so that 67% of females do not drink and 36% of males. Based on a South African study which used both surveys and biomarkers [25].	Central estimates<br>Consumption - 4.50%<br>Spend + 17.83 %<br>Lives saved 23,011<br>Cases saved 1,010,354<br><br>Alternative scenario<br>Consumption – 4.58%<br>Spend + 17.61%<br>Lives saved 19,884<br>Cases saved 852,591

For peer review only

HIV baseline estimates	iHME 2018 estimates<br><br>female<br>63,360 deaths<br>4,142,101 cases<br><br>male<br>69,502 deaths<br>2,535,616 cases	Thembisa 2018 estimates<br><br>female<br>39,983 deaths<br>4,426,113 cases<br><br>male<br>40,941 deaths<br>2,529,253 cases	Stakeholders highlighted the difference between GBD estimates and local estimates for HIV deaths. The Thembisa model was built by local academics and is used by UNAIDs [26].	Central estimates<br>Lives saved 23,011<br>Cases saved 1,010,354<br>HIV lives saved 11,866<br>HIV cases averted 488,928<br><br>Alternative scenario<br>Lives saved 17,790<br>Cases saved 1,014,852<br>HIV lives saved 6370<br>HIV cases averted 478,988
Socioeconomic gradients of ill health	HIV<br>Q1 (poorest) – 20%<br>Q2 – 36%<br>Q3 – 32%<br>Q4 – 9%<br>Q5 – 3%<br><br>Intentional Injury/Road Injury/Liver Cirrhosis<br>Q1 – 9%<br>Q2 – 29%<br>Q3 – 26%<br>Q4 – 26%<br>Q5 – 10%<br><br>Breast cancer<br>Q1 – 7%<br>Q2 – 7%<br>Q3 – 22%<br>Q4 – 18%<br>Q5 – 47%	Scenario 1<br>Changing the liver cirrhosis gradient to match the one used for breast cancer<br><br>Scenario 2<br>HIV<br>Q1 (poorest) – 25%<br>Q2 – 22%<br>Q3 – 20%<br>Q4 – 18%<br>Q5 – 14%<br><br>Intentional injury/ Road injury/Liver cirrhosis<br>Q1 – 20%<br>Q2 – 20%<br>Q3 – 19%<br>Q4 – 20%<br>Q5 – 22%	Scenario 1<br>Stakeholders indicated that for long-term conditions like cirrhosis wealthier groups could well be over-represented in SA. They suggested sensitivity analysis by applying values for a condition that is less concentrated amongst the poor.<br><br>Scenario 2<br>Recent data from another South African survey is used to provide plausible alternative socioeconomic gradients across all the conditions used in the model [27].	Central estimates<br>Liver cirrhosis<br>lives saved/cases averted<br>Q1 99 / 2,624<br>Q2 323 / 8,424<br>Q3 373 / 9,846<br>Q4 353 / 9,374<br>Q5 101 / 2,719<br><br>Scenario 1<br>Liver cirrhosis lives saved/cases averted<br>Q1 70 / 1,866<br>Q2 78 / 2,029<br>Q3 297 / 7,829<br>Q4 246 / 6,541<br>Q5 451 / 12,044<br><br>Central estimates aggregate<br>lives saved / cases averted<br>Q1 3,271 / 144,766<br>Q2 8,133 / 333,550<br>Q3 4,661 / 211,725

For peer review only

		Breast cancer<br>Q1 (poorest) – 21%<br>Q2 – 21%<br>Q3 – 20%<br>Q4 – 19%<br>Q5 – 18%		Q4 4,869 / 212,904<br>Q5 2,077 / 107,411<br><br>Scenario 2<br>aggregate<br>lives saved / cases averted<br>Q1 3,329 / 151,931<br>Q2 5,757 / 238,614<br>Q3 4,423 / 196,833<br>Q4 5,030 / 213,585<br>Q5 5,960 / 302,835
Discount rates for costs	5%	Scenario 1<br>0% discount rate	Discount rate was changed to 0%	Central estimate<br>Health costs saved<br>R7.69 billion<br><br>Scenario 1<br>Health costs saved<br>R12.40 billion
Homebrew switching	30%	Scenario 1<br>0%<br><br>Scenario 2<br>100%	The assumption that drinkers will make up 30% of the reduction in drinking recorded alcohol with homebrew comes from consultation with the stakeholders at workshop two. To test the importance of this assumption on the results a null impact and a 100% impact are introduced. 100% would mean that any homebrew drinkers will not receive any positive health impacts from the policy.	Central estimate<br>Consumption - 4.50%<br>Lives saved 23,011<br>Cases saved 1,010,354<br><br>Scenario 1<br>Consumption - 4.64%<br>Lives saved 23,644<br>Cases saved 1,035,109<br><br>Scenario 2<br>Consumption - 4.17 %<br>Lives saved 22,073<br>Cases saved 973,019

Table 15: Sensitivity analysis results

For peer review only

References

1. Kehoe T, Gmel G, Shield KD, Gmel G, Rehm J. Determining the best population-level alcohol consumption model and its impact on estimates of alcohol-attributable harms. *Population health metrics* 2012; **10**(1): 6.
2. Rehm J, Kehoe T, Gmel G, Stinson F, Grant B, Gmel G. Statistical modeling of volume of alcohol exposure for epidemiological studies of population health: the US example. *Population Health Metrics* 2010; **8**(1): 3.
3. Meier PS, Meng Y, Holmes J, et al. Adjusting for unrecorded consumption in survey and per capita sales data: quantification of impact on gender-and age-specific alcohol-attributable fractions for oral and pharyngeal cancers in Great Britain. *Alcohol and Alcoholism* 2013; **48**(2): 241-9.
4. Probst C, Shuper PA, Rehm J. Coverage of alcohol consumption by national surveys in South Africa. *Addiction* 2017; **112**(4): 705-10.
5. Gmel G, Shield KD, Kehoe-Chan TA, Rehm J. The effects of capping the alcohol consumption distribution and relative risk functions on the estimated number of deaths attributable to alcohol consumption in the European Union in 2004. *BMC medical research methodology* 2013; **13**(1): 24.
6. Brennan A, Meier P, Purshouse R, et al. The Sheffield alcohol policy model–a mathematical description. *Health economics* 2015; **24**(10): 1368-88.
7. Venables WN, Ripley BD. Modern Applied Statistics with S. Fourth Edition. New York: Springer; 2002.
8. SA Wine Industry Information and Systems. SA WINE INDUSTRY 2019 STATISTICS NR 44. South Africa, 2019.
9. Shield K, Manthey J, Rylett M, et al. National, regional, and global burdens of disease from 2000 to 2016 attributable to alcohol use: a comparative risk assessment study. *The Lancet Public Health* 2020; **5**(1): e51-e61.
10. Probst C, Parry CD, Rehm J. HIV/AIDS mortality attributable to alcohol use in South Africa: a comparative risk assessment by socioeconomic status. *BMJ open* 2018; **8**(2): e017955.
11. Rebublic of South Africa. Mid-year population estimates, 2019. In: Statistics South Africa, editor.; 2019.
12. Holmes J, Meier PS, Booth A, Guo Y, Brennan A. The temporal relationship between per capita alcohol consumption and harm: a systematic review of time lag specifications in aggregate time series analyses. *Drug and alcohol dependence* 2012; **123**(1-3): 7-14.
13. Republic of South Africa. Guidelines for Pharmacoeconomic Submissions. In: Department of Health, editor.: Government Gazette; 2012.
14. UNAIDS. UNAIDS South Africa: Overview. 2020. https://www.unaids.org/en/regionscountries/countries/southafrica (accessed 27/02/2020.
15. Matzopoulos RG, Prinsloo M, Butchart A, Peden MM, Lombard CJ. Estimating the South African trauma caseload. *International Journal of Injury Control and Safety Promotion* 2006; **13**(1): 49-51.
16. Vento S, Dzudzor B, Cainelli F, Tachi K. Liver cirrhosis in sub-Saharan Africa: neglected, yet important. *The Lancet Global Health* 2018; **6**(10): e1060-e1.
17. Joffe M, Ayeni O, Norris SA, et al. Barriers to early presentation of breast cancer among women in Soweto, South Africa. *PloS one* 2018; **13**(2): e0192071.
18. Meyer-Rath G, van Rensburg C, Chiu C, Leuner R, Jamieson L, Cohen S. The per-patient costs of HIV services in South Africa: Systematic review and application in the South African HIV Investment Case. *PloS one* 2019; **14**(2): e0210497.
19. Bola S, Dash I, Naidoo M, Aldous C. Interpersonal violence: quantifying the burden of injury in a South African trauma centre. *Emergency medicine journal* 2016; **33**(3): 208-12.
20. Parkinson F, Kent S, Aldous C, Oosthuizen G, Clarke D. The hospital cost of road traffic accidents at a South African regional trauma centre: A micro-costing study. *Injury* 2014; **45**(1): 342-5.
21. Health Systems Trust. District Health Barometer 2018/19, 2020.

For peer review only

22. Guzha N, Thebe T, Butler N, Valodia P. Development of a method to determine the cost of breast cancer treatment with chemotherapy at Groote Schuur Hospital, Cape Town, South Africa. *South African Medical Journal* 2020; **110**(4): 296-301.
23. Van Walbeek C, Blecher E. The economics of alcohol use, misuse and policy in South Africa. 2014. http://www.tobaccoecon.uct.ac.za/sites/default/files/image_tool/images/405/People/the-economics-of-alcohol-policy-in-south-africa.pdf (accessed 10/03/2019.
24. Selvanathan S, Selvanathan EA. The Demand for Alcohol, Tobacco and Marijuana:

International Evidence. Aldershot, England: Ashgate Publishing; 2005.
25. Pisa PT, Vorster HH, Kruger A, Margetts B, Loots DT. Association of alcohol consumption with specific biomarkers: A cross-sectional study in south africa. *Journal of health, population, and nutrition* 2015; **33**(1): 146.
26. Johnson LF, May MT, Dorrington RE, et al. Estimating the impact of antiretroviral treatment on adult mortality trends in South Africa: A mathematical modelling study. *PLoS medicine* 2017; **14**(12): e1002468.
27. Kabudula CW, Houle B, Collinson MA, et al. Socioeconomic differences in mortality in the antiretroviral therapy era in Agincourt, rural South Africa, 2001–13: a population surveillance analysis. *The Lancet Global Health* 2017; **5**(9): e924-e35.

For peer review only

CHEERS Checklist

Section/topic	#	Recommendation	Reported on page #/ line #
**TITLE AND ABSTRACT**			
Title	1	Identify the study as an economic evaluation or use more specific terms such as "cost-effectiveness analysis", and describe the interventions compared.	This is a modelling study, of all the checklists offered this was the most relevant one
Abstract	2	Provide a structured summary of objectives, perspective, setting, methods (including study design and inputs), results (including base case and uncertainty analyses), and conclusions.	page 2 lines 12:17
Abstract			
**INTRODUCTION**			
Background and objectives	3	Provide an explicit statement of the broader context for the study.<br>Present the study question and its relevance for health policy or practice decisions.	Page 4
**METHODS**			
Target population and subgroups	4	Describe characteristics of the base case population and subgroups analysed, including why they were chosen.	Page 5
Setting and location	5	State relevant aspects of the system(s) in which the decision(s) need(s) to be made.	Page 5 Figure 1
Study perspective	6	Describe the perspective of the study and relate this to the costs being evaluated.	Costs were chosen by stakeholders page 5 figure 1
Comparators	7	Describe the interventions or strategies being compared and state why they were chosen.	Page 5 Lines 50 - 55

For peer review only

CHEERS Checklist

Time horizon	8	State the time horizon(s) over which costs and consequences are being evaluated and say why appropriate.	Page 6 lines 40:49
Discount rate	9	Report the choice of discount rate(s) used for costs and outcomes and say why appropriate.	Page 6 lines 57:58
Choice of health outcomes	10	Describe what outcomes were used as the measure(s) of benefit in the evaluation and their relevance for the type of analysis performed.	Page 6 lines 29:38
Measurement of effectiveness	11a	*Single study-based estimates*: Describe fully the design features of the single effectiveness study and why the single study was a sufficient source of clinical effectiveness data	NA
	11b	*Synthesis-based estimates*: Describe fully the methods used for identification of included studies and synthesis of clinical effectiveness data.	NA
Measurement and valuation of preference based outcomes	12	If applicable, describe the population and methods used to elicit preferences for outcomes	NA

Section/topic	#	Checklist item	Reported on page #
Estimating resources and costs	13a	*Single study-based economic evaluation*: Describe approaches used to estimate resource use associated with the alternative interventions. Describe primary or secondary research methods for valuing each resource item in terms of its unit cost. Describe any adjustments made to approximate to opportunity costs.	NA
	13b	*Model-based economic evaluation*: Describe approaches and data sources used to estimate resource use associated with model health states. Describe primary or secondary research methods for valuing each resource item in terms of its unit cost. Describe any adjustments made to approximate to opportunity costs.	Page 6 lines 54-59
Currency, price date, and conversion	14	Report the dates of the estimated resource quantities and unit costs. Describe methods for adjusting estimated unit costs to the year of reported costs if necessary. Describe methods for converting costs into a common currency base and the exchange rate.	Page 6 line 57
Choice of model	15	Describe and give reasons for the specific type of decision-analytical model used. Providing a figure to show model structure is strongly recommended.	Page 5 figure 1
Assumptions	16	Describe all structural or other assumptions underpinning the decision-analytical model.	Page 5 -6
Analytical models	17	Describe all analytical methods supporting the evaluation. This could include methods for dealing with skewed, missing, or censored data; extrapolation methods; methods for pooling data; approaches to validate or make adjustments (such as half cycle corrections) to a model; and methods for handling population heterogeneity and uncertainty.	Appendix parts 1 - 12
**RESULTS**			

For peer review only

CHEERS Checklist

Study parameters	18	Report the values, ranges, references, and, if used, probability distributions for all parameters/ Report reasons or sources for distributions used to represent uncertainty where appropriate. Providing a table to show the input values is strongly recommended.	Page 5 line 60 appendix arts 2,6,9,11
Incremental costs and outcomes	19	For each intervention, report mean values for the main categories of estimated costs and outcomes of interest, as well as mean differences between the comparator groups. If applicable, report incremental cost-effectiveness ratios.	Page 9 table 1,2 Page 10 table 3
Characterising uncertainty	20a	*Single study-based economic evaluation*: Describe the effects of sampling uncertainty for the estimated incremental cost and incremental effectiveness parameters, together with the impact of methodological assumptions (such as discount rate, study perspective).	NA
	20b	*Model-based economic evaluation*: Describe the effects on the results of uncertainty for all input parameters, and uncertainty related to the structure of the model and assumptions.	NA
Characterising heterogeneity	21	If applicable, report differences in costs, outcomes, or cost-effectiveness that can be explained by variations between subgroups of patients with different baseline characteristics or other observed variability in effects that are not reducible by more information.	Page 9/10 Tables 1 - 3 Figures 3&4
**Section/topic**	**#**	**Checklist item**	**Reported on page #**
**DISCUSSION**			
Study findings, limiations, generalisability, and current knowledge	22	Summarise key study findings and describe how they support the conclusions reached. Discuss limitations and the generalisability of the findings and how the findings fit with current knowledge.	Page 11-12
**Other**			
Source of funding	23	Describe how the study was funded and the role of the funder in the identification, design, conduct, and reporting of the analysis. Describe other non-monetary sources of support.	Page 12 lines 30:39
Conflicts of interest	24	Describe any potential for conflict of interest of study contributors in accordance with journal policy. Int eh absence of a journal policy, we recommend authors comply with International Committee of Medical Journal Editors recommendations.	Page 12

For peer review only

BMJ Open

Effects of minimum unit pricing for alcohol in South Africa across different drinker groups and wealth quintiles: a modelling study.

Journal:	*BMJ Open*
Manuscript ID	bmjopen-2021-052879.R1
Article Type:	Original research
Date Submitted by the Author:	06-Jul-2021
Complete List of Authors:	Gibbs, Naomi; The University of Sheffield Faculty of Medicine Dentistry and Health, School of Health and Related Research<br>Angus, Colin; The University of Sheffield School of Health and Related Research, ScHARR<br>Dixon, Simon; University of Sheffield School of Health and Related Research<br>Parry, Charles; South African Medical Research Council, Alcohol & Drug Abuse Research Unit<br>Meier, Petra; University of Glasgow
**Primary Subject Heading**:	Public health
Secondary Subject Heading:	Epidemiology, Health economics, Addiction, Global health, Health policy
Keywords:	Epidemiology < TROPICAL MEDICINE, HEALTH ECONOMICS, PUBLIC HEALTH, Health policy < HEALTH SERVICES ADMINISTRATION & MANAGEMENT, HIV & AIDS < INFECTIOUS DISEASES

SCHOLARONE™
Manuscripts

I, the Submitting Author has the right to grant and does grant on behalf of all authors of the Work (as defined in the below author licence), an exclusive licence and/or a non-exclusive licence for contributions from authors who are: i) UK Crown employees; ii) where BMJ has agreed a CC-BY licence shall apply, and/or iii) in accordance with the terms applicable for US Federal Government officers or employees acting as part of their official duties; on a worldwide, perpetual, irrevocable, royalty-free basis to BMJ Publishing Group Ltd ("BMJ") its licensees and where the relevant Journal is co-owned by BMJ to the co-owners of the Journal, to publish the Work in this journal and any other BMJ products and to exploit all rights, as set out in our licence.

The Submitting Author accepts and understands that any supply made under these terms is made by BMJ to the Submitting Author unless you are acting as an employee on behalf of your employer or a postgraduate student of an affiliated institution which is paying any applicable article publishing charge ("APC") for Open Access articles. Where the Submitting Author wishes to make the Work available on an Open Access basis (and intends to pay the relevant APC), the terms of reuse of such Open Access shall be governed by a Creative Commons licence – details of these licences and which Creative Commons licence will apply to this Work are set out in our licence referred to above.

Other than as permitted in any relevant BMJ Author's Self Archiving Policies, I confirm this Work has not been accepted for publication elsewhere, is not being considered for publication elsewhere and does not duplicate material already published. I confirm all authors consent to publication of this Work and authorise the granting of this licence.

For peer review only

TITLE: EFFECTS OF MINIMUM UNIT PRICING FOR ALCOHOL IN SOUTH AFRICA ACROSS DIFFERENT DRINKER GROUPS AND WEALTH QUINTILES: A MODELLING STUDY.

AUTHORS

Naomi K Gibbs, Colin Angus, Simon Dixon, Charles DH. Parry, Petra S. Meier

AUTHOR AFFILIATIONS

N K Gibbs, C Angus: School of Health and Related Research, University of Sheffield, UK

Prof S Dixon: School of Health and Related Research, University of Sheffield, UK. Honorary Professor, Priority Cost Effective Lessons for Systems Strengethening, South Africa (PRICELESS SA), School of Public Health, Faculty of Health Sciences, University of the Witwatersrand

Prof C D H Parry: Alcohol, Tobacco and Other Drug Research Unit, South African Medical Research Council, South Africa

Prof P S Meier: MRC/CSO Social and Public Health Sciences Unit, University of Glasgow

CORRESPONDANCE TO:

Naomi Gibbs, ScHARR, University of Sheffield, S1 4DA, UK

n.gibbs@sheffield.ac.uk

0114 222 5454

WORDCOUNT:

Abstract: 258

Main text: 3498

ABBREVIATIONS

MUP: Minimum unit pricing

HIV: Human immunodeficiency virus

VAT: Value added tax

SADHS: South African Demographic and Health Survey

IAC: International Alcohol Control study

PIF: Potential impact fraction

For peer review only

ABSTRACT

Objectives

To quantify the potential impact of Minimum Unit Pricing (MUP) for alcohol on alcohol consumption, spending and health in South Africa. We provide these estimates disaggregated by different drinker groups and wealth quintiles.

Design

We developed an epidemiological policy appraisal model to estimate the effects of MUP across sex, drinker groups (moderate, occasional binge drinker, heavy) and wealth quintiles. Stakeholder interviews and workshops informed model development and ensured policy relevance.

Setting

South African drinking population aged 15+.

Participants

The population (aged 15+) of South Africa in 2018, stratified by drinking group and wealth quintiles, with a model time horizon of 20 years.

Main outcome measures

Change in standard drinks (SD) (12 grams of ethanol) consumed, weekly spend on alcohol, annual number of cases and deaths for five alcohol-related health conditions, reported by drinker groups and wealth quintile.

Results

We estimate a MUP of R10 per SD would lead to an immediate reduction in consumption of 4.40% (-0.95 SD/week) and an increase in spend of 18.09%. The absolute reduction is greatest for heavy drinkers (-1.48 SD/week), followed by occasional binge drinkers (-0.41 SD/week) and moderate drinkers (-0.40 SD/week). Over 20 years we estimate 20,585 fewer deaths and 900,332 cases averted across the five health modelled harms.

Poorer drinkers would see greater impacts from the policy (consumption: -7.75% in poorest quintile, -3.19% in richest quintile). Amongst the heavy drinkers 85% of the cases averted and 86% of the lives saved accrue to the bottom three wealth quintiles.

Conclusions

We estimate that MUP would reduce alcohol consumption in South Africa, improving health outcomes whilst raising retail and tax revenue. Consumption and harm reductions would be greater in poorer groups.

For peer review only

3

ARTICLE SUMMARY

Strengths and limitations of this study

- This study presents the first epidemiological policy appraisal model of a minimum unit price applied to South Africa. Previous similar modelling work has been limited to high-income countries.
- Our model provides equity relevant information by presenting results disaggregated by wealth quintiles, allowing for a more nuanced consideration of the potential impact of the policy.
- Our model has also benefited from a thorough programme of stakeholder engagement ensuring contextual and policy relevance.
- A key limitation is the alcohol pricing data which is drawn from a relatively small sample in one locality. Further research would benefit from improved pricing data, specifically the different prices paid for alcohol by different population groups.
- Secondly the model has not explored the financial impact on the poorest groups beyond increased alcohol expenditure. Further research should include exploration of broader financial benefits such as reduced private expenditure on healthcare or improved labour market outcomes.

For peer review only

BACKGROUND

In South Africa there are high levels of reported abstinence coupled with high levels of binge drinking amongst those who do drink, resulting in significant levels of alcohol-related harm [1]. This harm is not distributed evenly throughout society with the lower socioeconomic groups experiencing higher levels of harm, particularly for infectious diseases such as HIV [2]. The periodic prohibition of alcohol during the covid-19 lockdown demonstrates political leaders' acceptance that alcohol causes harm to South Africa and signals a potential willingness to take strong action [3]. Provincial governments, such as the Western Cape, are considering a number of alcohol policy approaches, including the introduction of Minimum Unit Pricing (MUP) [4].

MUP is a policy whereby a legal floor price is introduced, below which a fixed volume of ethanol cannot be sold to the public. It has already been introduced in several areas which experience high levels of alcohol harm, including Scotland and Australia's Northern Territory. Evidence suggests that MUP has been effective at reducing alcohol consumption, particularly among the heaviest drinkers, as they commonly drink the very cheap alcohol targeted by this policy [5,6].

A limitation of transferring the current evidence for MUP is its focus on high income countries. Transferring this evidence to South Africa would be problematic as it has very different drinking patterns, a very different harm profile with infectious disease and injury contributing significantly to the burden of alcohol, it has an informal sector which is challenging to capture and it has very high levels of income inequality likely to result in differential baseline prices and price responsiveness.

The current alcohol landscape is rooted in the country's recent political history. In 1926 apartheid legislation prohibited African and Indian access to licensed premises or employment by licence holders. As a result when the democratically elected government took power in 1994 they inherited a significant number of shebeens. Shebeens are (largely) unlicensed bars or pubs, found in townships, often open late and with a reputation for violence and risky sexual behaviour. Homebrew (mainly beer made from sorghum or other ingredients such as pineapple) can be purchased from shebeens along with other types of branded alcohol supplied by large alcohol manufacturers and mainly distributed through larger licensed outlets using bulk discounts. Although beer is the most popular drink, the consumption of large quantities of cheap wine is also prevalent and can be linked back to farm labourers being paid in cheap wine [7].

The South African government currently use alcohol excise tax to compensate for some of the social costs they attribute to alcohol consumption [8,9]. The system is based on targets for the proportion of the price that constitutes tax (excise tax plus Value Added Tax (VAT)). This varies by drink type with wine lowest followed by beer then spirits. The government have indicated a willingness to innovate and pursue public health improvements via fiscal policy with the introduction of a sugar tax in 2018. However, in a country with high levels of socioeconomic inequality there are concerns regarding possible financial impact of pricing policies on the poorest groups [10]. Evidence on public health pricing policies often fails to consider distributional impact by income-groups [11].

When designing public health economic models for unique policy contexts ongoing engagement with local stakeholders is essential. The purpose of engagement is twofold: to shape the direction of the research using expert local knowledge (including understanding the problem, guiding model development and ensuring policy relevance); and to provide channels for communication creating potential for the evidence to contribute to policy design [12-14].

We aimed to: 1) present estimates of the change to alcohol consumption, individual expenditure, retail and tax revenue following the introduction of a South African MUP, using a purpose built model, 2) estimate the impact on a limited number of alcohol-related health conditions and associated healthcare costs, 3) explore the potential equity implications via the demonstration of impact by both drinker

For peer review only

5

group and wealth quintile, 4) highlight parameters that are particularly influential to the results and areas that require further research.

METHODS

We built an epidemiological policy appraisal model coded in R (code available here), using a comparative risk assessment approach with multistate life tables [15]. A stakeholder mapping exercise was carried out following scoping conversations with three academic experts from three South African institutions. Following this, a shortlist of policy professionals, civil society members and local academics was drawn up and checked via the scientific and ethical review process. They were engaged via scoping interviews and three workshops, at the beginning, middle and end of the modelling process. Stakeholders informed key decisions including the specific policy to simulate, levels of the MUP, health outcomes of interest, assumptions on homebrew switching behaviour and validation of our choice of data sources.

Two distinct sections of the model were defined (Figure 1):

I. Price to consumption: Baseline prices were estimated for drinker groups (heavy drinkers, occasional binge drinkers, moderate drinkers) and wealth groups. Consumption was estimated at the individual level, this includes the proportion of alcohol drunk that is homebrew. Following a change in price the new price and subsequent consumption levels were estimated. This accounts for both mean and peak weekly alcohol consumption.
II. Consumption to harm: The relationship between mean and peak consumption and alcohol-related harm and associated costs were estimated.

FIGURE 1

There is no single dataset that can provide all the required data for the model and thus a combination of survey datasets, market research data and evidence from published literature was used (Figure 2).

FIGURE 2

Price to consumption

Baseline consumption and prices

Our model started by estimating mean and peak alcohol consumption at current alcohol prices at the individual level. We categorised drinkers into three exhaustive and mutually exclusive groups; moderate (less than 15 standard drinks per week); occasional binge (less than 15 drinks per week but more than 5 on one occasion); and heavy (15 or more drinks per week). A standard drink in South Africa is currently 15ml or 12 grams of pure ethanol. We generated price distributions for wealth and drinker groups using real price data linked to individual drinking from the International Alcohol Control Study (IAC)[16] survey 2014/2015 completed in the metropolitan district of Tshwane. The IAC asked for highly detailed data about prices in both on- and off-trade locations and took into account container size, drink type and number of drinks purchased. Alcohol was treated as one commodity as the 863 price observations were distributed between drinker and wealth groups instead of by alcohol type. Wealth quintiles were chosen as our measure of socioeconomic status as income was not available in the pricing dataset whereas asset ownership and common demographic data such as age, sex and education was. A detailed description of the above is given in the appendix parts 1 to 6.

For peer review only

Applying a minimum unit price

A government policy of legislating for a MUP of R5, R10 and R15 per South African standard drink was introduced. Prices below the MUP threshold were increased to the threshold, while products above were unaffected. We did not include prices for homebrew. The distribution of prices faced by each wealth/drinker group was used to calculate the mean price per standard drink before and after the policy. This then provided a percentage change in mean price (Table 1).

Elasticity of demand for alcohol

The change in price was translated into a change in individual consumption using an elasticity of demand for alcohol. We used previously published elasticities for South Africa, calculated separately by drinker group: -0.4, -0.22, -0.18 for moderate, occasional binge and heavy drinkers respectively [4] and adjusted for wealth quintile using additional evidence from South Africa[17] (Table 1) (Appendix 7).

Table 1: Price per standard drink (15ml of pure ethanol) before the policy by wealth quintile and drinker group

	Q1	Q2	Q3	Q4	Q5
**Baseline prices**					
**Moderate**	R9.13	R9.13	R9.13	R11.6	R11.6
**Occasional Binge**	R7.97	R10.0	R10.1	R13.4	R11.1
**Heavy**	R7.78	R9.65	R9.23	R10.6	R12.8
**Percentage change in mean price following R10 MUP**					
**Moderate**	22%	22%	22%	20%	20%
**Occasional Binge**	37%	16%	24%	11%	19%
**Heavy**	33%	26%	25%	24%	21%
**Price elasticities used in the model**					
**Moderate**	-0.53	-0.53	-0.31	-0.31	-0.31
**Occasional Binge**	-0.29	-0.29	-0.17	-0.17	-0.17
**Heavy**	-0.24	-0.24	-0.14	-0.14	-0.14
**Q1 = poorest**					

Those that drink both recorded alcohol and homebrew dampened the policy impact by switching some of their drinking to homebrew. Stakeholders indicated 30% of the reduction in recorded alcohol could be assumed as being compensated for via an equivalent increase in homebrew. This was varied between 0% (no switching) and 100% (full switching) in the sensitivity analysis.

Individual spend, tax and retail revenue

Total retail spend was computed by aggregating population weighted individual spend. This figure was increased by 1.25 (100/80) as consumption was calibrated to 80% of official sales volume data[18].

As a MUP is applied before VAT is calculated we estimated VAT as 15% of the total aggregate spend. Excise tax was calculated by starting with the total 2018 alcohol excise tax revenue from the Treasury Budget Report [9]. This was adjusted by percentage change in volume of alcohol sold (we used a fixed ratio between volume and excise tax). Retail revenue was calculated by taking VAT and excise taxation away from total spend (Appendix 8).

Consumption to harm

Relative risks and potential impact fractions

We used published estimates of relative risks associated with different levels of alcohol consumption (Appendix 9). We then used these to calculate relative risks for each outcome for each individual.

For peer review only

7

We utilised Potential Impact Fractions (PIFs), a widely-used approach in epidemiological modelling to estimate the impact of a change in exposure to risk on a change in outcomes [19]. We incorporated population weights and computed the PIFs by sex, wealth group and drinker groups (Appendix 10).

Baseline health

Baseline deaths and cases (population prevalence) of the five disease and injury conditions (HIV, road injury, intentional injury, liver cirrhosis and breast cancer) were apportioned by drinker group, sex and wealth quintile. The probability of death for each disease was calculated for baseline and taken away from overall probability of death for each single year of age given in the life table to give a probability of death from non-modelled causes. This probability of death from non-modelled causes remained constant at every policy scenario. The probability of death from the five diseases of interest then varied according to the policy level and the corresponding potential impact fraction. A more detailed description is given in the appendix 11 to 12.

Projecting the population

We modelled counterfactual population structure (i.e. in the absence of the policy) over 20 years, starting from 2018 [20]. We created multistate life tables in which the population faces a probability of mortality for each of the five disease/injury conditions and for non-modelled causes each year. The model generated alternative population impact fractions (as above) for baseline and for each policy scenario. Using the relevant population impact fraction and rerunning the multistate life table enabled a calculation of the difference between baseline and the policy. HIV, road injuries and intentional injuries realise the full impact of the reduction in drinking immediately whereas the health impact on liver cirrhosis and breast cancer are subject to lags in the effect [21] (Appendix 13).

The life tables for the 20 year time horizon were used in combination with the probability of having the disease and the potential impact fraction under each policy, to estimate the number of cases.

Hospital costs

Prevalence of disease/injury at each policy scenario for each year of the model was multiplied by the proportion who receive hospital treatment and the relevant hospital cost applied (Appendix 14). We converted all costs to 2018 prices using the Consumer Price Index [22]. Future costs were discounted at 5%.

Sensitivity analysis

We explored key uncertainties in the model using scenario analysis informed by previous published alcohol modelling work [23], our knowledge of the limitations of the data and stakeholder input. For each alternative scenario, relevant results were compared with central estimates. The key parameters explored were elasticities, proportion of abstainers, HIV baseline estimates, socioeconomic gradients of health, proportion of switching to homebrew and discount rates for costs. (Appendix 15).

Patient and Public Involvement

Patients were not involved in this study.

For peer review only

RESULTS

Estimated consumption and spend impact

Our findings are presented primarily for a R10 MUP but with some comparisons across all three pricing levels. The policy appraisal results are reported by quintile further disaggregated by drinker group (Table 2). In the model drinking prevalence increases with wealth (27% up to 38%) as does the prevalence of heavy drinking, ranging from 14% amongst Q1 up to 20% for Q5. Amongst all drinker groups mean consumption is either similar or demonstrates no clear pattern between wealth quintiles. On aggregate there was a gradient in average baseline weekly spend with the rich paying an average R257.36 per week compared to R148.03 in the lowest wealth group.

Our model estimated, for a MUP of R10, an immediate reduction in population alcohol consumption of 4.40% (-0.93 SD/week) and an increase in spend of 18.09%. Moderate drinkers showed the greatest percentage decrease in their drinking, followed by occasional binge then heavy drinkers (-8.71%, -4.51%, -4.19%). However this translated to a larger absolute reduction in consumption for heavier drinkers (-1.48 SD/week) than either moderate or occasional binge drinkers (-0.40, -0.41).

For peer review only

Table 2: Consumption and spend R10 policy estimates

	Overall	Q1	Q2	Q3	Q4	Q5
**Survey respondents**	10,336 (100%)	2,098 (19%)	2,227 (19%)	2,337 (21%)	2,066 (20%)	1,608 (21%)
**All drinkers**						
**n (%)***	3311 (33%)	551 (27%)	690 (30%)	823 (33%)	685 (35%)	562 (38%)
Baseline consumption (standard drinks per week)	21.22	20·83	21·40	20.97	21.98	20·89
Baseline spending (R per week)	R208.74	R148.03	R192.82	R186.95	R231.78	R257.36
Change in consumption (%)	-4·40%	-7.75%	-6.42%	-3.76%	-3.41%	-3.19%
Change in consumption (standard drinks per week)	-0·93	-1.50	-1.29	-0.76	-0·72	-0.65
Change in spending (R per week)	R37·95	R32.81	R32.52	R38.27	R42.64	R43.07
**Moderate**						
**n (%)***	1336 (12%)	206 (10%)	272 (13%)	354 (12%)	273 (13%)	231 (15%)
Baseline consumption (standard drinks per week)	5·05	5.01	5.49	4.90	4.86	4.98
Baseline spending (R per week)	R49·97	R42.75	R48.84	R43.54	R54.38	R56.59
Change in consumption (%)	-8.71%	-12.20%	-12.89%	-7.14%	-6.35%	-6.43%
Change in consumption (standard drinks per week)	-0.40	-0.55	-0.63	-0.33	-0.29	-0.30
Change in spending (R per week)	R5.79	R3.52	R3.91	R6.16	R6.97	R7.30
**Occasional Binge**						
**n (%)***	433 (4%)	76 (4%)	89 (4%)	109 (5%)	91 (4%)	68 (4%)
Baseline consumption (standard drinks per week)	9.53	9.69	9.27	9.59	9.27	9.82
Baseline spending (R per week)	R96.87	R68.13	R84.04	R94.63	R120.59	R109.00
Change in consumption (%)	-4.51%	-10.16%	-4.21%	-4.05%	-1.89%	-3.32%
Change in consumption (standard drinks per week)	-0.41	-0.89	-0.37	-0.37	-0.17	-0.32
Change in spending (R per week)	R14.58	R16.69	R9.28	R17.86	R11.31	R16.42
**Heavy**						
**n (%)***	1542 (16%)	269 (14%)	329 (14%)	360 (16%)	321 (17%)	263 (20%)
Baseline consumption (standard drinks per week)	36.72	35.02	39.53	36.20	38.22	35.16
Baseline spending (R per week)	R360.19	R244.13	R356.68	R320.18	R394.90	R439.14
Change in consumption (%)	-4·19%	-7.15%	-5.75%	-3.41%	-3.23%	-2.85%
Change in consumption (standard drinks per week)	-1.48	-2.34	-2.15	-1.19	-1.19	-0.97
Change in spending (R per week)	R69.68	R57.17	R65.17	R67.85	R77.47	R75.08

Data for 10,336 survey respondents.
*Numbers refer to absolute sample size, percentages incorporate survey weights, the relevant base is indicated in the top row of their column.
Q1 = poorest

For peer review only

Our model estimated that there would be an increase in individual spend on alcohol consumption of R32.77 billion in the year following the introduction of the policy. The government would see an increase in VAT as a result of the increased prices although a reduction in excise taxation due to the reduced volume of alcohol sold. Retail revenue would also increase (Table 3).

Table 3: Aggregate spend, taxation and retail revenue

Change from baseline in billion Rand, per year			
	**R5 MUP**	**R10 MUP**	**R15 MUP**
**Individual spend**	R1·24	R32·77	R78·29
**Taxation**			
VAT	R0·16	R4·27	R10·21
Excise tax	-R0·03	-R1·24	-R3·40
**Retail revenue**	R1·11	R29·74	R71·48

Estimated health impact

Across the five health conditions included in the model a R10 minimum price estimated 20,585 lives saved and 900,332 cases averted of the disease/injury conditions over the 20 year time horizon. For R5 (R15) we estimated 95, (45,326) lives saved and 4,126, (2,038,319) cases averted respectively. The impact differed by drinker group and by wealth quintile (Figure 3). The greatest health benefits accrued to the heaviest drinkers, with the dominant effect related to HIV infections, especially in the bottom three quintiles. Amongst the heavy drinkers 85% of the cases averted and 86% of the lives saved accrued to the bottom three quintiles. Occasional binge drinkers achieved most of their positive health impact via a reduction in interpersonal violence and road injury as both of these conditions are linked to binge drinking. There was a small increase of HIV incidence amongst occasional binge drinkers. The high prevalence of HIV is the source of an important competing risk and the avoidance of death related to acute conditions led to longer exposure to the risk of HIV infection. As expected the cases saved of liver cirrhosis accrued to the heavy drinkers as this condition relates to heavy drinking in the long term. Q2 realised the highest number of HIV cases averted due to having the highest proportion of cases at baseline.

FIGURE 3

Health care cost savings accrued over the 20 years and were greatest for intentional injury (Table 4). The health cost savings are provided by quintile in appendix 16.

Table 4: Healthcare cost savings over 20 years, millions

	**R5 MUP**	**R10 MUP**	**R15 MUP**
Antiretroviral therapy costs	-R0.15	R565.82	R1356.51
Intentional injury hospital costs	R32.55	R4304.13	R9088.97
Road injury hospital costs	R16.46	R1975.45	R4265.68
Liver cirrhosis hospital costs	R0.66	R27.60	R68.19
Breast cancer hospital costs	R0.22	R4.00	R10.59

Results across policy levels

Comparing across the three policy levels demonstrates the relative impact between wealth quintiles remained largely consistent as the MUP level increased for moderate and occasional binge drinkers (Figure 4) although for heavy drinkers the wealth gradient becomes more pronounced at R15 particularly for change in consumption.

For peer review only

11

FIGURE 4

The sensitivity analysis that produced the most variable results were the alternative elasticity estimates. Two of the alternative scenarios (-0.8 applied to all drinkers, and -0.86/-0.5 applied to Q1 and Q2 with -0.5 applied to Q3 – Q5) produced much greater consumption impacts (-14%, – 18%) coupled with much smaller increases in individual spend (5.4%, 0.1%). All other results are included in appendix 15.

DISCUSSION

Our analysis estimates that MUP may offer an effective approach to reducing alcohol consumption and related harm in South Africa. For a MUP of R10, we estimate an immediate reduction in consumption of 4.40%, increase in individual spend of 18.09% and an increase in retail revenue and taxation. In terms of health impact we estimate 20,585 lives saved and 900,332 cases averted in total across HIV, intentional injury, road injury, liver cirrhosis and breast cancer over 20 years. Regarding the equity impact our model estimates the distribution of health outcomes is generally pro poor, critically important given these groups also see the greatest relative increase in their alcohol expenditure.

Our research aligns with studies from other countries which suggest minimum pricing will reduce alcohol sales and also corresponds to mechanisms, such as greater impact with a rising MUP threshold and greater impact on the poor, found in the international literature [24,25]. We add to the South African minimum pricing evidence currently available [26] by incorporating health outcomes, accommodating homebrew and exploring differential impact by wealth groups. Van Walbeek and Chelwa [26] who produced an economic model to simulate the impact of a MUP on consumption (with no epidemiological modelling) suggest both a higher reduction in consumption and greater difference in consumption impact between heavy and moderate drinkers. The difference in our estimates is largely due to different price estimates. Their prices are crucially far more heterogeneous between drinker groups, outweighing the impact of the price elasticities. Our prices are drawn from a detailed survey asking for real prices paid by beverage, container and location which allows us to calculate real prices per standard drink. Van Walbeek and Chelwa used an average unit value derived from reported monthly alcohol consumption (calculated using quantity/frequency questions) and one variable asking for monthly spend on alcohol [26] which gave very low prices for heavy drinkers. Their prices may be too low and ours too high for the heaviest drinkers. If this is the case our findings may present a conservative estimate of the potential impact of the policy.

Our study has a number of strengths relevant to providing policy relevant research in LMICs. In the absence of detailed market research purchasing data we demonstrate how survey, administrative data and the academic literature can be used, in partnership with local stakeholders, to build a contextually relevant epidemiological policy appraisal model. A further strength is our focus on stakeholder engagement from project inception increasing the likelihood of findings being taken into consideration during policy decision making [27]. MUP was chosen as the policy to model as it was seen as both innovative and potentially well targeted for the South African heavy drinking culture. Stakeholders were pleased the estimates combined improved health with increased taxation and increased retail revenue, as supporting business was considered politically important. The financial cost of MUP is borne by drinkers and there were concerns about how this may impact poorer groups and we recommend this as an area for further research. However, in a country which suffers a very high degree of alcohol related harm coupled with very high abstinence levels, it may be considered an improvement by some that drinkers, rather than the whole of society, pay.

A limitation of our study is the lack of high quality pricing data for South Africa. Previous studies in HIC have found that moderate drinkers, even those on lower incomes, purchase relatively little cheap

For peer review only

alcohol [24], while the price data used in our model suggests that all drinker groups purchase some cheap alcohol. It is unclear whether this is a true reflection of alcohol purchasing patterns in SA or a limitation of the data. In addition, although we adjusted the off-trade wine prices to be consistent with industry sources we know that the proportion of wine in the survey is less than the market share. As wine constitutes some of the cheapest available alcohol a MUP may have a bigger impact than our estimates suggest. If the price of wine increased we may expect drinkers to switch to other cheaper alcohol, however, a key strength of MUP is that the policy applies across all alcohol types and so drinkers are not able to do this.

Related to this limitation is the treatment of all drinks as one commodity. South African evidence has suggested cheap wine has a much higher price elasticity than other drink types [8]. If cheap wine is the drink primarily effected then this elasticity would lead to less of an increase in individual spend, potentially even a saving, smaller gains to retailers and less of a loss to excise tax revenue as wine enjoys substantially lower rates.

We recommend the following avenues for further research. Firstly, the collection of improved pricing data, specifically the different prices paid for alcohol by different population groups, to explore further the most appropriate level of MUP. Secondly, the exploration of the financial impact on the poorest groups including any financial benefits such as reduced expenditure on healthcare or improved labour market outcomes. Thirdly, in an alcohol market that includes retailers operating outside of the regulated space (despite largely selling recorded alcohol purchased from licensed outlets) it would be important to understand enforcement mechanisms and the supply chain in order for the policy to maximise effectiveness. However, it should be noted that the IAC pricing data suggests most of the lowest prices are to be found at large supermarkets and bottle stores which offer bulk discounts rather than small local shebeens that sell alcohol often to be drunk on the premises.

CONCLUSION

Our model estimates minimum pricing would reduce alcohol consumption in South Africa, improving health outcomes whilst raising retail and tax revenue. Consumption and harm reductions would be greater in poorer compared to richer groups. We estimate that minimum pricing is a targeted policy that has the potential to bring health and financial benefits to a country which suffers a very high burden of alcohol-related harm.

Acknowledgments

We would like to thank all stakeholders who have shaped the research. This includes contributors from National Government, Western Cape Department of the Premier, Western Cape Liquor Authority, South African Alcohol Policy Alliance, Douglas Murray Trust, Khayelitsha Community Health Forum, Violence Protection through Urban Upgrading, Research Unit of the Economics of Excisable Products at University of Cape Town, South African Medical Research Council and the University of the Western Cape. We would also like to thank Charlotte Probst and Nick Stacey for their scientific review of the work.

Contributors

All authors conceptualised the study. NKG completed the modelling and stakeholder engagement under the supervision of CA, SD, PSM and CDHP. NKG wrote the first draft. All authors refined various drafts of the manuscript and approved the final version.

Competing interests

We declare no competing interests.

Data sharing statement

No additional data available.

For peer review only

13

Funding

This work was supported by the Wellcome Trust Doctoral Training Centre in Public Health Economics and Decision Science [108903/Z/19/Z] and the University of Sheffield [No award number]. Also the South African Medical Research Council [No award number]. The funders of the study had no role in the study. All authors had full access to all the data in the study and were responsible for the decision to submit the article for publication.

Ethics committee approval

Ethical approval for engaging with stakeholders was granted by the South African Medical Research Council (Protocol ID: EC005-4/2019) and the School of Health and Related Research at the University of Sheffield, UK (Reference Number: 023357). All data for the model came from secondary sources and was managed according to an approved information governance plan.

For peer review only

REFERENCES

1. Shield K, Manthey J, Rylett M, et al. National, regional, and global burdens of disease from 2000 to 2016 attributable to alcohol use: a comparative risk assessment study. *The Lancet Public Health* 2020; **5**(1): e51-e61.

2. Probst C, Parry CD, Wittchen H-U, Rehm J. The socioeconomic profile of alcohol-attributable mortality in South Africa: a modelling study. *BMC medicine* 2018; **16**(1): 97.

3. Matzopoulos R, Walls H, Cook S, London L. South Africa's COVID-19 Alcohol Sales Ban: The Potential for Better Policy-Making. *International Journal of Health Policy and Management* 2020.

4. Van Walbeek C, Chelwa G. Using price-based interventions to reduce abusive drinking in the Western Cape Province. 2019.

5. O'Donnell A, Anderson P, Jané-Llopis E, Manthey J, Kaner E, Rehm J. Immediate impact of minimum unit pricing on alcohol purchases in Scotland: controlled interrupted time series analysis for 2015-18. *bmj* 2019; **366**: l5274.

6. Robinson M, Mackay D, Giles L, Lewsey J, Beeston C. Evaluating the Impact of Minimum Unit Pricing (MUP) on Sales-Based Consumption in Scotland: Controlled Interrupted Time Series Analysis. *Public Health Scotland: Edinburgh, Scotland* 2020.

7. McLoughlin J-A, Little F, Mazok C, Parry C, London L. Prevalence of and associations with papsak wine consumption among farm workers in the Western Cape Province, South Africa. *Journal of studies on alcohol and drugs* 2013; **74**(6): 879-88.

8. Republic of South Africa. A review of the taxation of alcoholic beverages in South Africa. In: National Treasury, editor.; 2014.

9. Treasury N. Budget Review 2020. In: Treasury N, editor.; 2020.

10. Ataguba JE-O. Alcohol policy and taxation in South Africa. *Applied health economics and health policy* 2012; **10**(1): 65-76.

11. Jain V, Crosby L, Baker P, Chalkidou K. Distributional equity as a consideration in economic and modelling evaluations of health taxes: A systematic review. *Health Policy* 2020; **124**(9): 919-31.

12. Roberts M, Russell LB, Paltiel AD, Chambers M, McEwan P, Krahn M. Conceptualizing a model: a report of the ISPOR-SMDM modeling good research practices task force–2. *Medical Decision Making* 2012; **32**(5): 678-89.

13. Squires H, Chilcott J, Akehurst R, Burr J, Kelly MP. A Framework for Developing the Structure of Public Health Economic Models. *Value in Health* 2016; **19**(5): 588-601.

14. Howick S, Eden C, Ackermann F, Williams T. Building confidence in models for multiple audiences: The modelling cascade. *European Journal of Operational Research* 2008; **186**(3): 1068-83.

15. Briggs AD, Wolstenholme J, Blakely T, Scarborough P. Choosing an epidemiological model structure for the economic evaluation of non-communicable disease public health interventions. *Population health metrics* 2016; **14**(1): 17.

16. IAC. International Alcohol Control Study. 2021. https://www.iacstudy.org/ (accessed 01/07/2021 2021).

17. Van Walbeek C, Blecher E. The economics of alcohol use, misuse and policy in South Africa. 2014. http://www.tobaccoecon.uct.ac.za/sites/default/files/image_tool/images/405/People/the-economics-of-alcohol-policy-in-south-africa.pdf (accessed 10/03/2019).

18. Euromonitor International. Country Report: Alcoholic Drinks in South Africa. 2021. https://www.portal.euromonitor.com/portal/analysis/tab (accessed 01/07/2021 2021).

19. Gunning-Schepers L. The health benefits of prevention: a simulation approach. *Health Policy* 1989; **12**(1-2): 1-255.

20. Rebublic of South Africa. Mid-year population estimates, 2019. In: Statistics South Africa, editor.; 2019.

21. Holmes J, Meier PS, Booth A, Guo Y, Brennan A. The temporal relationship between per capita alcohol consumption and harm: a systematic review of time lag specifications in aggregate time series analyses. *Drug and alcohol dependence* 2012; **123**(1-3): 7-14.

22. Republic of South Africa. CPI History. In: Statistics South Africa, editor.; 2020.

For peer review only

23. Brennan A, Meier P, Purshouse R, et al. The Sheffield alcohol policy model–a mathematical description. *Health economics* 2015; **24**(10): 1368-88.
24. Holmes J, Meng Y, Meier PS, et al. Effects of minimum unit pricing for alcohol on different income and socioeconomic groups: a modelling study. *The Lancet* 2014; **383**(9929): 1655-64.
25. Robinson M, Mackay D, Giles L, Lewsey J, Richardson E, Beeston C. Evaluating the impact of minimum unit pricing (MUP) on off-trade alcohol sales in Scotland: an interrupted time–series study. *Addiction* 2021.
26. Van Walbeek C, Chelwa G. The case for minimum unit prices on alcohol in South Africa. *South African Medical Journal* 2021; **111**(7): 680-4.
27. Bradley BD, Jung T, Tandon-Verma A, Khoury B, Chan TC, Cheng Y-L. Operations research in global health: a scoping review with a focus on the themes of health equity and impact. *Health research policy and systems* 2017; **15**(1): 32.

FIGURES

Figure 1: Conceptual model framework

Figure 2: Data inputs for model

Figure 3: Cases averted by condition, split by drinker group and wealth quintile

Figure 4: Comparing the three policy levels: change in mean weekly drinks and cases averted by drinker and wealth group

For peer review only

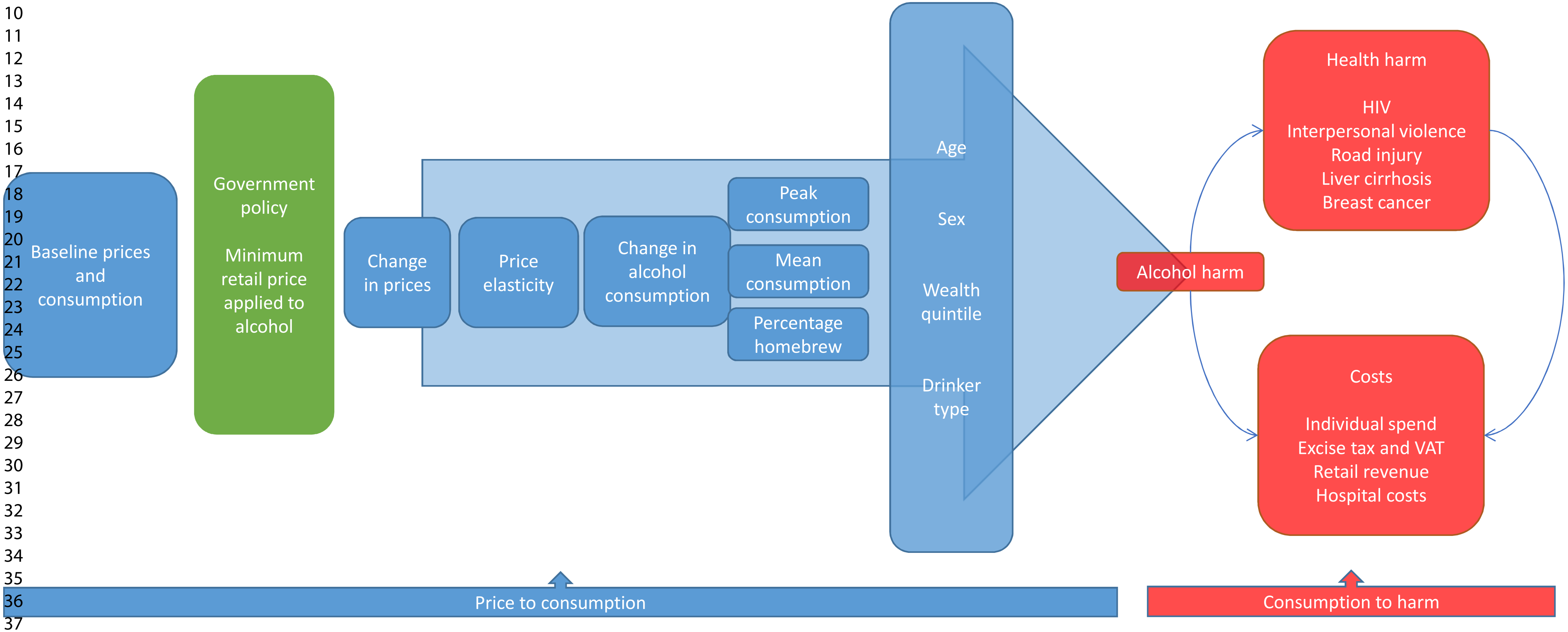

Baseline prices and consumption
Government policy
Minimum retail price applied to alcohol
Change in prices
Price elasticity
Change in alcohol consumption
Peak consumption
Mean consumption
Percentage homebrew
Age
Sex
Wealth quintile
Drinker type
Alcohol harm
Health harm
HIV
Interpersonal violence
Road injury
Liver cirrhosis
Breast cancer
Costs
Individual spend
Excise tax and VAT
Retail revenue
Hospital costs
Price to consumption
Consumption to harm

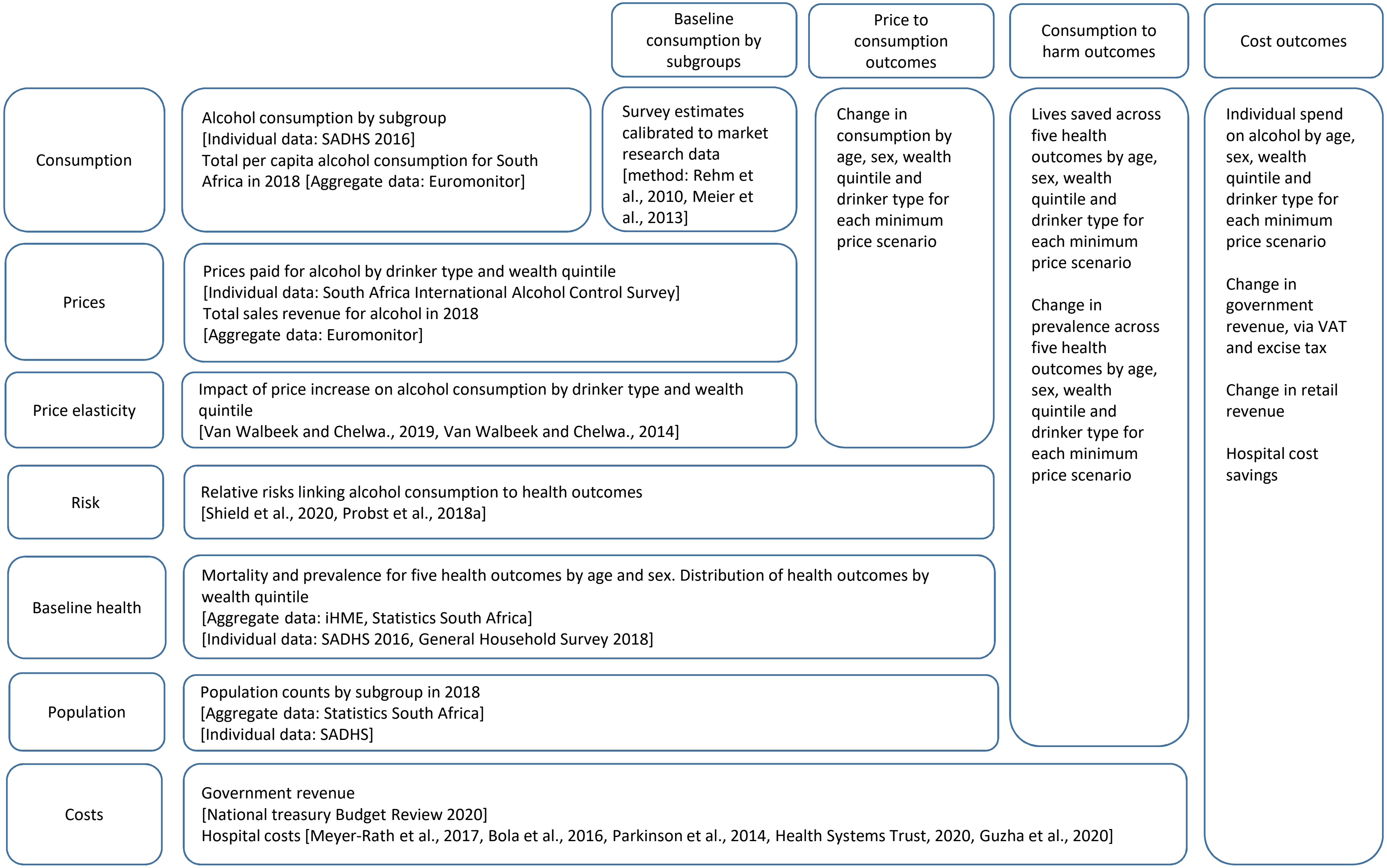
Baseline consumption by subgroups
Price to consumption outcomes
Consumption to harm outcomes
Cost outcomes
Consumption
Alcohol consumption by subgroup [Individual data: SADHS 2016] Total per capita alcohol consumption for South Africa in 2018 [Aggregate data: Euromonitor]
Survey estimates calibrated to market research data [method: Rehm et al., 2010, Meier et al., 2013]
Change in consumption by age, sex, wealth quintile and drinker type for each minimum price scenario
Lives saved across five health outcomes by age, sex, wealth quintile and drinker type for each minimum price scenario
Change in prevalence across five health outcomes by age, sex, wealth quintile and drinker type for each minimum price scenario
Individual spend on alcohol by age, sex, wealth quintile and drinker type for each minimum price scenario
Change in government revenue, via VAT and excise tax
Change in retail revenue
Hospital cost savings
Prices
Prices paid for alcohol by drinker type and wealth quintile [Individual data: South Africa International Alcohol Control Survey] Total sales revenue for alcohol in 2018 [Aggregate data: Euromonitor]
Price elasticity
Impact of price increase on alcohol consumption by drinker type and wealth quintile [Van Walbeek and Chelwa., 2019, Van Walbeek and Chelwa., 2014]
Risk
Relative risks linking alcohol consumption to health outcomes [Shield et al., 2020, Probst et al., 2018a]
Baseline health
Mortality and prevalence for five health outcomes by age and sex. Distribution of health outcomes by wealth quintile [Aggregate data: iHME, Statistics South Africa] [Individual data: SADHS 2016, General Household Survey 2018]
Population
Population counts by subgroup in 2018 [Aggregate data: Statistics South Africa] [Individual data: SADHS]
Costs
Government revenue [National treasury Budget Review 2020] Hospital costs [Meyer-Rath et al., 2017, Bola et al., 2016, Parkinson et al., 2014, Health Systems Trust, 2020, Guzha et al., 2020]

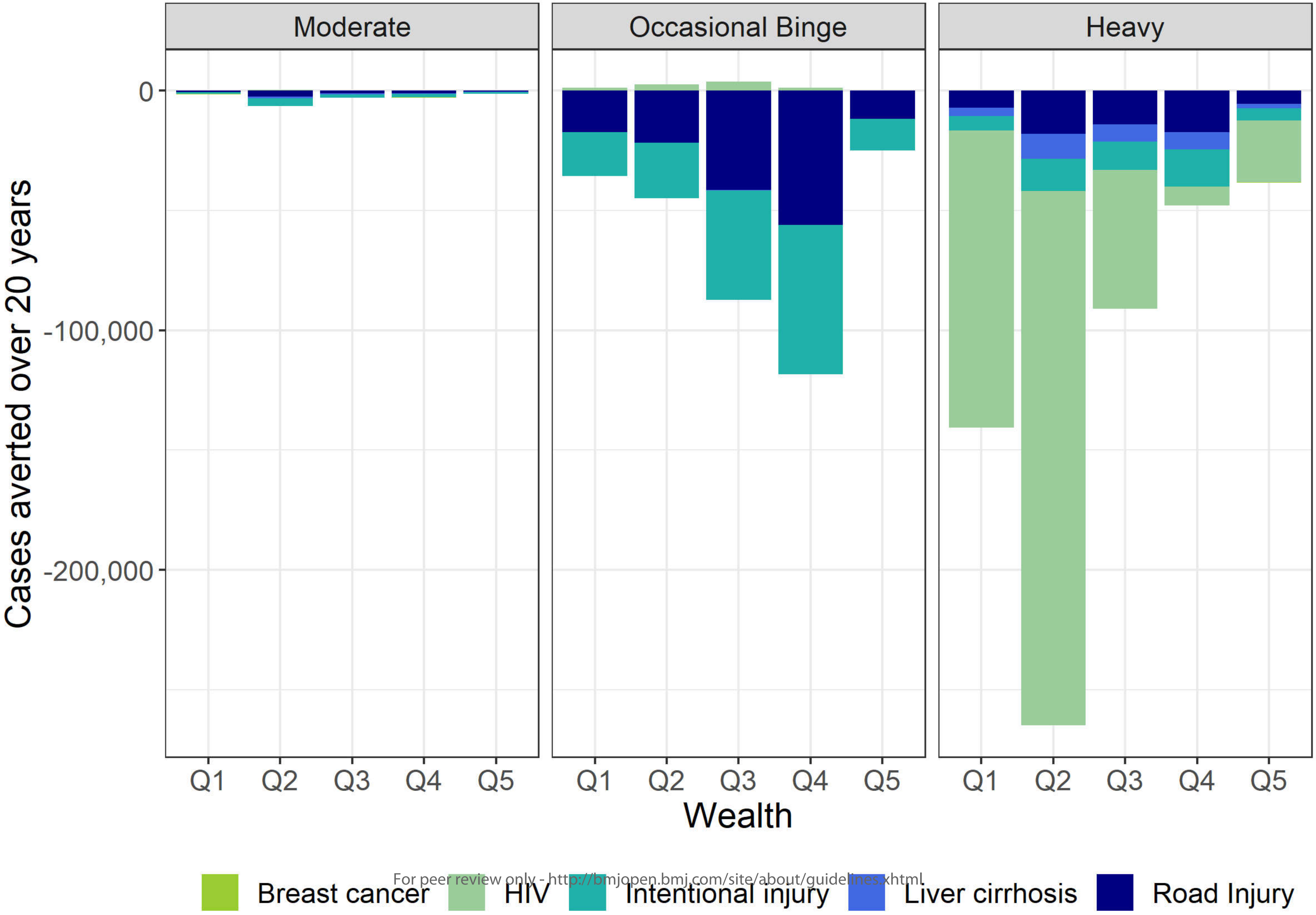
Moderate
Occasional Binge
Heavy
0
-100,000
-200,000
Cases averted over 20 years
Q1
Q2
Q3
Q4
Q5
Wealth
Breast cancer
HIV
Intentional injury
Liver cirrhosis
Road Injury

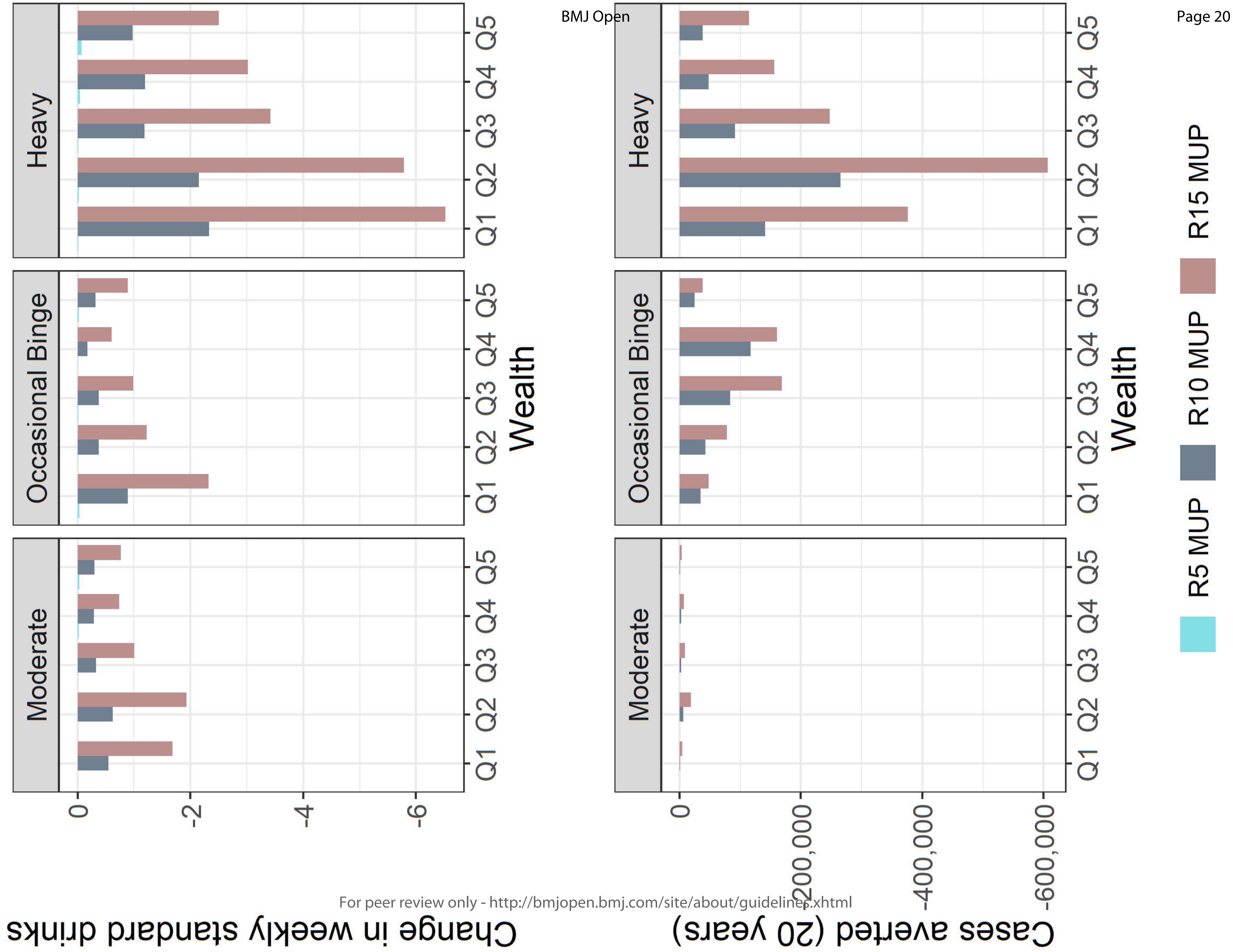
Heavy
Occasional Binge
Moderate
Q1
Q2
Q3
Q4
Q5
Wealth
0
-2
-4
-6
Change in weekly standard drinks
0
-200,000
-400,000
-600,000
Cases averted (20 years)
R5 MUP
R10 MUP
R15 MUP

Contents

Supplementary Material 2
Price to consumption 2
1. Estimating baseline consumption using South African Demographic and Health Survey (SADHS) 2
Process of adjusting the SADHS estimates 2
Process of adjusting peak drinks 3
2. Uplifting consumption 6
3. Uplifting peak consumption 8
4. Wealth quintiles 9
5. International Alcohol Control Study 2014 for prices 10
6. Base prices by subgroup 12
7. Adjusting the elasticities 12
8. Individual spend, tax and retail revenue 13
Consumption to harm 14
9. Relative risks 14
10. Potential impact fractions 16
11. Socioeconomic gradients of ill health 17
12. Distributing baseline deaths and cases and calculating probabilities 18
13. Baseline health and lagged health impact 20
14. Hospital multipliers and costs 21
15. Sensitivity Analysis 24
16. Healthcare cost savings by quintile 28
References 29

For peer review only

Supplementary Material

Price to consumption

Our model starts by estimating mean and peak alcohol consumption at current alcohol prices at the individual level. The proportion of alcohol consumption which is homebrew is also estimated. This process utilised both alcohol frequency questions and seven day recall questions asked in the same survey. As survey data significantly underreports consumption we calibrate these estimates to market research data using statistical methods established in the literature [1-3]. Following the shift of mean consumption, peak consumption is re-estimated using a simple regression model created at baseline. We categorise drinkers into three exhaustive and mutually exclusive groups; moderate (less than 15 standard drinks per week); occasional binge (less than 15 drinks per week but more than 5 on one occasion); and heavy (15 or more drinks per week). A standard drink in South Africa is currently 15ml or 12 grams of pure ethanol. We compute a regression model for wealth quintiles using the South African Demographic and Health Survey (SADHS) data and use it to predict wealth quintiles in the International Alcohol Control (IAC) dataset to generate price distributions for wealth and drinker groups. Alcohol is treated as one commodity due to data constraints.

1. Estimating baseline consumption using South African Demographic and Health Survey (SADHS)

The SADHS survey asked the following questions:

Table 1: Survey questions

Survey	Alcohol questions<br>[answers]
**SADHS 2016**	Have you ever consumed a drink that contains alcohol such as beer, wine, ciders, spirits, or sorghum beer?<br>Probe: Even one drink?<br>[yes, no]<br><br>Was this within the last 12 months?<br>[yes, no]<br><br>In the last 12 months, how frequently have you had at least one drink?<br>[5 or more days a week, 1-4 days per week, 1-3 days a month, less often than once a month]<br><br>During each of the last 7 days, how many standard drinks did you have?<br>[use showcard, record total number of drinks consumed each day starting with the day before the day of the interview and proceeding backwards]<br><br>During the last 7 days, how many standard home-made beers or other homemade alcohol did you have?<br>[use showcard, record number]<br><br>In the past 30 days, have you consumed five or more standard drinks on at least one occasion?<br>[yes, no]

Process of adjusting the SADHS estimates

Drinkers were categorised by their drinking frequency and by whether or not they had reported any drinking in the last seven days.

For peer review only - http://bmjopen.bmj.com/site/about/guidelines.xhtml

Table 2: Frequency of alcohol consumption responses

Drinking occasion frequency	count	Reported drinks in last 7 days	Reported zero drinks in last 7 days
5 or more days a week	293	266	27 (6 binge, 21 drinker)
1-4 days per week	668	565	103 (29 binge, 74 drinker)
1-3 days per month	1163	799	364 (all drinkers)
less often than once a month	1187	404	783 (all drinkers)
NA	7025		

Firstly readjusting those with a seven day drinking pattern (pink numbers)
The pink numbers are respondents who say they only drink 1 -3 days per month or less often than once a month but they have drank in the last 7 days. If this were multiplied by 52 it would be an overestimate.

Drink frequency 1 – 3 days per month
Assume for those that drink 1-3 days per month we have caught their one drinking week in the month and multiply by 12 to get their annual consumption. There are 799 people in this category.

Drink frequency less than once per month
Assume for those who drink less often than once a month but who did drink in the last week we have caught their one drinking week in the month. Multiply by 6, to get the annual figure. There are 404 people in this category.

The yellow numbers are ok as respondents report drinking every week and have a seven day drinking pattern.

Readjusting those without a seven day drinking pattern but who say they drink (blue and red numbers)
Drink frequency 5 or more days per week
Use the mean standard drinks for drinkers (and binge drinkers) who reported the same frequency but who do have a seven day pattern, there are 266 people (yellow). This is computed separately for sex and drinker group.
Drink frequency 1 - 4 days per week
Use the mean standard drinks for drinkers (and binge drinkers) who report the same frequency but who do have a seven day pattern, there are 565 people (yellow). This is computed separately for sex and drinker group.
Drink frequency 1 – 3 days per month
Use the mean adjusted annual drinks (adjusted above) of the equivalent frequency group who did report a drinking pattern, said no to drinking 5 or more, split by sex. This uses observations from 799 people (pink).
Drink frequency less than once per month
Use the mean adjusted annual drinks (adjusted above) of the equivalent frequency group who did report a drinking pattern, said no to drinking 5 or more, and split by sex, This uses observations from 404 people (pink).

Alternatively those who drink less than once a month, assume they drink 2.5 standard drinks (midpoint of 1 – 4) 6 times a year. This would mean they drink 2.5 drinks * 6 months = 15 standard drinks, again this is of a similar magnitude.

For peer review only

Process of adjusting peak drinks
Using the same process as above we also applied a peak drink to those observations which did not have one. However before this we checked all those who reported binge drinking had a peak drink at minimum of 5, which they did.

Comparing the adjusted SADHS data with the estimates using only 7 day recall we see that as expected prevalence of drinking increases and per capita estimates reduce (Table 3).

Table 3: Comparing adjusted with unadjusted statistics

	Prevalence of drinking		Sample size: drinkers	Annual litres of alcohol - per capita			Annual litres of alcohol - Just drinkers		
	Female	Male		Total	Female	Male	Total	Female	Male
**SADHS (7 day recall only)**	9.3%	32.7%	n = 1949 (report drinks	2.2	0.65	4.5	10.6	6.54	12.2

**Population weights applied**			in the 7 day recall) females = 571 males = 1378						
**SADHS adjusted (7 day recall plus adjustments based on frequency questions) Population weights applied**	18%	54%	n = 3311 females = 1125 males = 2186	1.65	0.50	3.4	5.0	2.59	6.25

Figure 1: Density plot of female drinkers before and after the shift

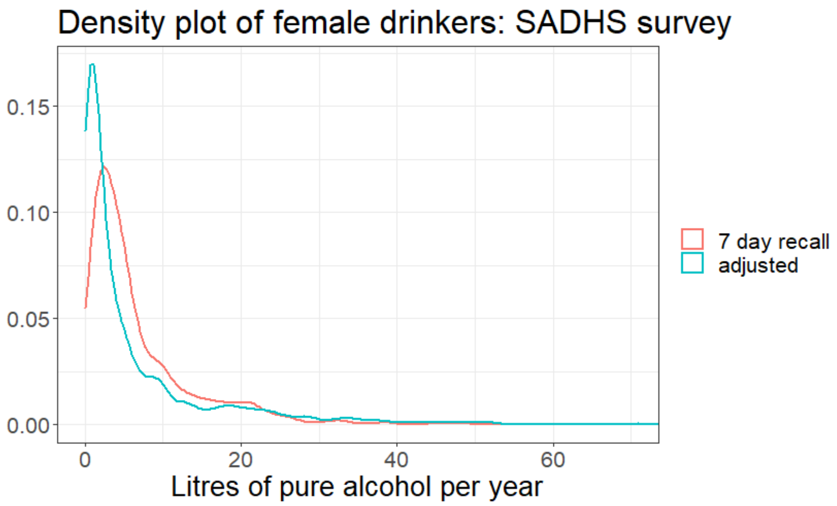

Figure 2: Density plot of male drinkers before and after the shift

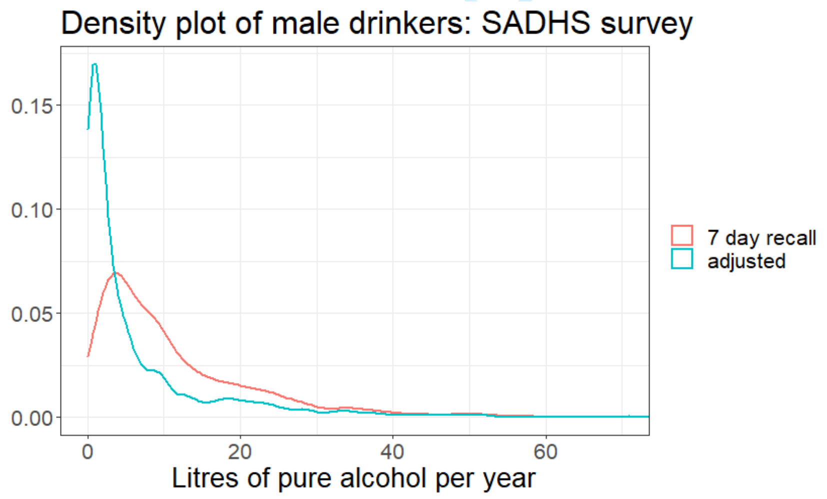

Incorporating the frequency data into the seven day recall moves the distribution towards the left (Figures 1 and 2). This is logical as the sample will now include those drinkers who stated that they drink but did not record any for the last seven days, it also adjusted down those who claim to drink less than weekly but who did recall drinks for the last seven days. This pattern gives some confidence in the dataset and utilises the strengths of capturing heavy drinking well and including occasional drinkers.

For peer review only

2. Uplifting consumption

Surveys provide important data about drinking patterns within the population but, as previously mentioned, they provide total consumption estimates that are far smaller than that indicated by government sources or market research data [4]. There are established ways of adjusting alcohol consumption survey data to account for this via statistical calibration techniques [2,3]. The following steps outline how we tailored the methods to the South Africa context:

- First a cap was applied to all drinkers of 68 litres of alcohol per year or 150 grams of alcohol per day. As the model includes long term effects (20 years) the cap is needed as a higher level of alcohol cannot be sustained in the long term [5]. This cap impacted one woman and ten men. Of this small group only two men drunk both homebrew and recorded alcohol and so their total consumption was reduced to 68 litres and then split into recorded and homebrew using their previous percentage split.

- Survey coverage level was calculated as the difference between total per capita consumption recorded in the SADHS survey and per capita consumption using Euromonitor recorded sales data for 2018. 80% of the sales data is used to account for spillage, stockpiling and tourist consumption. This sales figure was then increased to take account of the 4.15% of total alcohol consumed in the survey reported as homebrew (representing unrecorded alcohol in the model). The comparison of total consumption according to the survey and the adjusted official sales data was used to create the multiplication factor.

- For female and male subgroups the mean litres of alcohol was adjusted by the multiplication factor.

- This new mean and the relationship between mean and standard deviation, established in the literature [2], was used to compute a standard deviation and then fit a "shifted" gamma distribution (maintaining the cap of 68 litres) using the mean and standard deviation, calculated for male and females separately.

$$\hat{\sigma}_{shifted} = 1.174 \times \hat{\mu}_{shifted} + 1.003 \times sex$$

- It is possible to stop at this point; however, there are two limitations with this method. Firstly, there is no empirical evidence that under coverage is distributed as implied by the shifts needed to fit the adjusted consumption to the gamma. Secondly, that shifting consumption to a gamma can artificially reduce the long tail of heavy drinkers [3].
- Therefore, a gamma distribution was fitted to the original sample of drinkers by sex and percentiles were taken across both distributions. Percentage differences in consumption were calculated at each percentile. These increases were then applied to the percentiles of the original survey sample
- Each individuals total consumption was split into homebrew and recorded alcohol using the original percentage split (this assumes underreporting is equal across homebrew and recorded alcohol)
- Results were compared (Table 4 and Figures 3 and 4). There is a fairly small difference between the two methods, more visible for males than females. It appears adjusting by percentiles only makes a difference at the extremes, lowering the left hand peak slightly but also falling below the Gamma shifted distribution after 60litres of alcohol per year meaning there is a smaller number of the very high drinkers. The percentile adjusted distribution was used for the main model.

Table 4: Comparing pre and post shift data

Females – litres of alcohol per year	Mean	Min	Max
**SADHS Survey data (weighted mean and capped)**	2.57	0.09	68
**Gamma fitted to survey (difference due to weights)**	2.50	0.09	68
**Gamma shift**	10.78	1	68
**Adjusting each percentile (weighted mean)**	10.74	0.5	68
Males – litres of alcohol per year			
**SADHS Survey data (weighted mean and capped)**	6.13	0.09	68
**Gamma fitted to survey (difference due to weights)**	5.6	0.09	68
**Gamma distribution shifted**	18.55	1	68
**Adjusting each percentile (weighted mean)**	19.2	0.5	68

For peer review only

Figure 3: Comparing distributions pre and post shift females

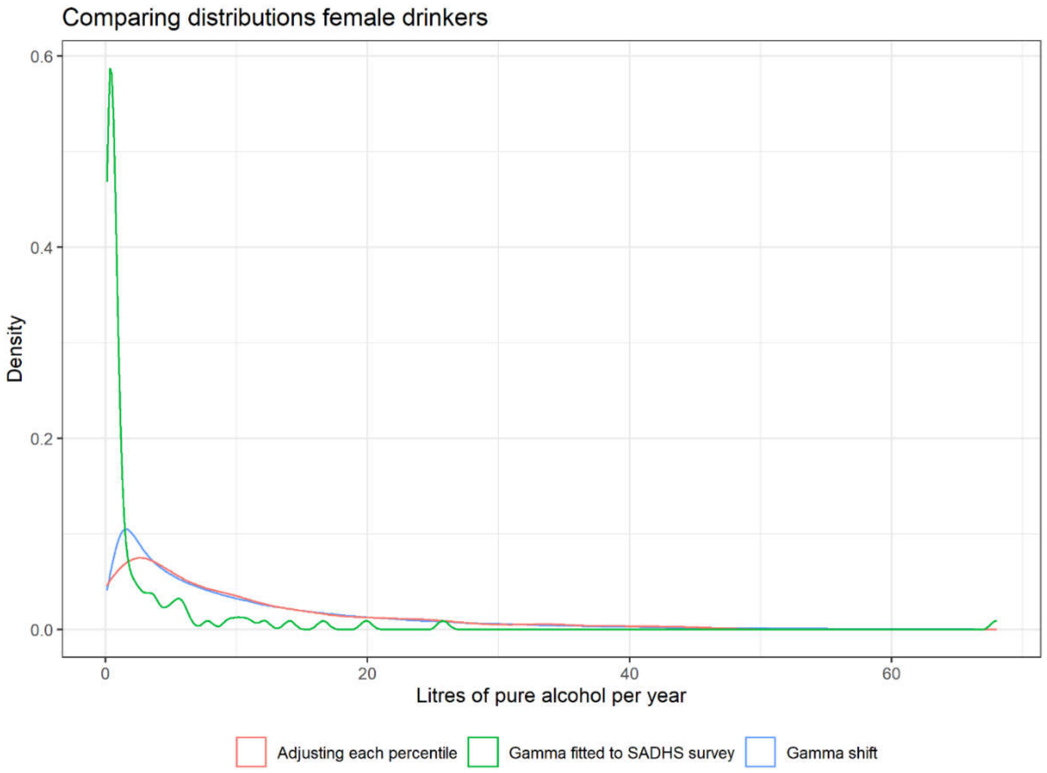

Figure 4: Comparing distributions pre and post shift males

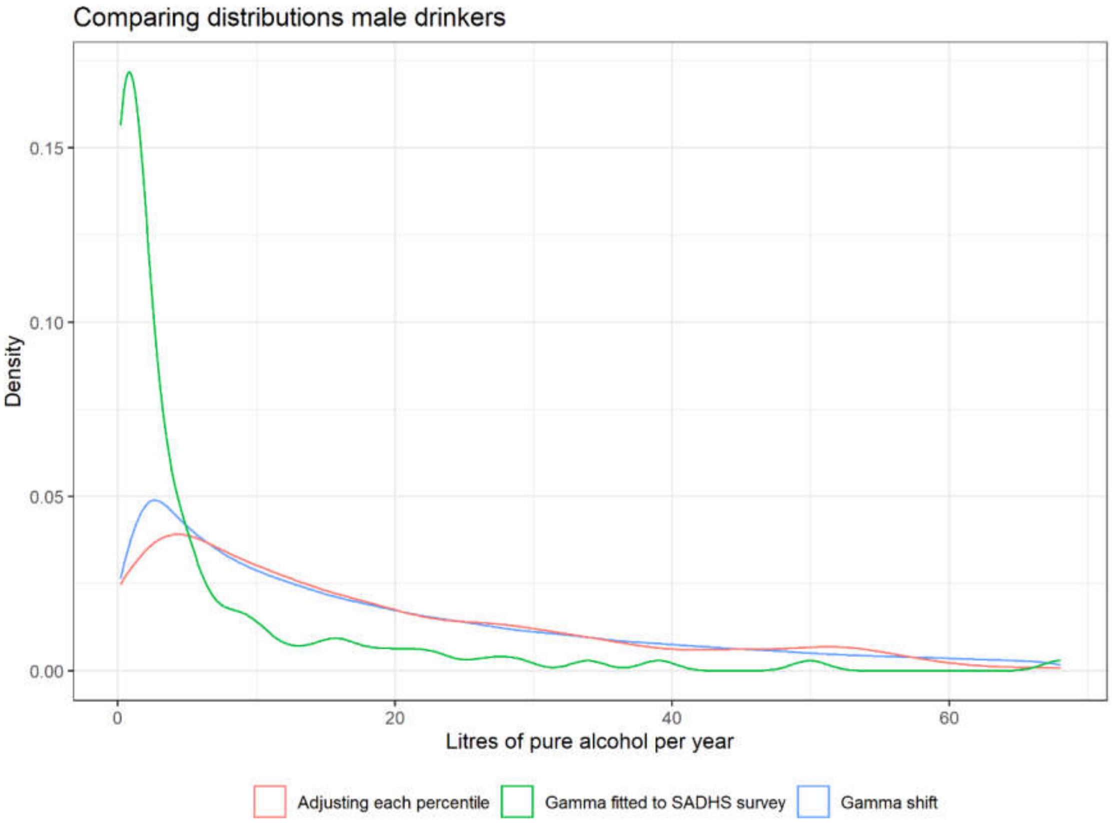

3. Uplifting peak consumption

Peak drinking is important as it will influence health harms directly. Following the method used in the Sheffield Alcohol Policy Model [6], the following linear regression model was fitted, for all drinkers, to the non-shifted SADHS data, relating peak drinking to mean consumption, age and sex.

```
peak_model <- lm(dk_peak_SADHS ~ lit_SADHS + age_band_4 + sex)
```

The model was used to compute fitted values for the non-shifted data. The model assumes there is a linear relationship between peak and mean consumption, the magnitude of which is allowed to vary by age and sex.

After the mean consumption was shifted as above the corresponding new peak consumption was computed using the following formula:

$$peak_{ij}\ (shifted) = peak_{ij}\ (SADHS) \times (E(peak_{ij}\ (shifted))/E(peak_{ij}\ (SADHS))$$

The linear relationship between mean and peak discovered in the SADHS survey is maintained for the shifted mean and peak consumption which assumes individuals under reported peak and mean consumption by the same magnitude. The prediction error for the model is of the same magnitude for all levels of consumption.

The predictions were checked to ensure that peak drinking was never predicted below mean daily drinking. There were 88 people (out of the 3311 drinkers) for whom this was true. These people had their peak drinking increased to match their mean daily drinking.

For peer review only

4. Wealth quintiles

In order to match wealth groups between the two datasets an ordered choice model was created using SADHS data with wealth quintile (1 – 5) as the dependent variable, using the MASS package in R [7]. Wealth groups were chosen as the best available measure to capture socioeconomic status that allowed us to match between the SADHS and IAC dataset. Although income was asked in the IAC dataset many of the respondents refused to answer resulting in a very small sample.

All the variables that were common across the two datasets were included in the initial model, these were not just asset ownership but also age, sex, educational level and population group (race). Stepwise regression was performed using the step.AIC function. This chooses the best variables to include by running the regression with all variables in and then taking one out and computing a goodness of fit measure (the AIC). If the goodness of fit measure is improved then that model is preferred, it runs this for many models until it finds the model with the highest AIC. This method resulted in the selection of the following variables: age, sex, population group, education level, car, landline, electricity, fridge, computer, radio, tv. The only variable it removed was mobile phone which fitted anecdotally with conversations we had with stakeholders in South Africa regarding how much poorer people prioritise mobile phones.

polr(formula = wealth ~ car + electricity + tv + fridge + radio + telephone + computer + age + edu + pop_group, data = SADHS, Hess = TRUE)

The goodness of fit matrix evaluates the success of the model, comparing the closeness of the predicted and observed outcome (Table 5). The model never predicts the poorest as the richest or the richest as the poorest.

Table 5: Goodness of fit matrix

	**Prediction**					
**Actual**		Poorest	Poorer	Middle	Richer	Richest
	Poorest	1300	593	196	9	0
	Poorer	299	975	744	192	17
	Middle	62	612	1042	595	26
	Richer	5	236	763	818	244
	Richest	0	10	108	422	1068

For peer review only

5. International Alcohol Control Study 2014 for prices

The IAC dataset provides prices by drinking location by beverage, by container size and also asks whether the individual binge drinks, demographic data is also collected. The survey asked for the price in Rands by location, for example they ask for the price of a beer paid at a pub for each container size. There are 17 drinking locations (12 on trade and 5 off trade) and 12 drink types. On-trade is where the alcohol is consumed on the premises it is purchased (e.g. hotels, restaurants, pubs), off-trade is where the alcohol is consumed off the premises it was purchased at (e.g. supermarket or bottle store).

Prices were disaggregated by population subgroups rather than by drink type (wine/beer/spirits etc). This was consistent with the South Africa specific price elasticities which were calculated for drinker groups whilst treating alcohol as a single commodity. The IAC respondents were categorised into drinker groups using the definitions above. Each price was weighted by the number of units (e.g. bottles, glasses, cans) sold, the container size of those units and the number of drinking occasions in 6 months (Figure 5). Every price observation was validated using data from the South African Consumer Price Index. Prices were increased to 2018 to account for inflation.

Figure 5: Distribution of off-trade and on-trade prices, standard drink is 15ml or 12grams of pure ethanol.

On-trade is where the alcohol is consumed on the premises it is purchased (e.g. hotels, restaurants, pubs), off-trade is where the alcohol is consumed off the premises it was purchased at (e.g. supermarket or bottle store).

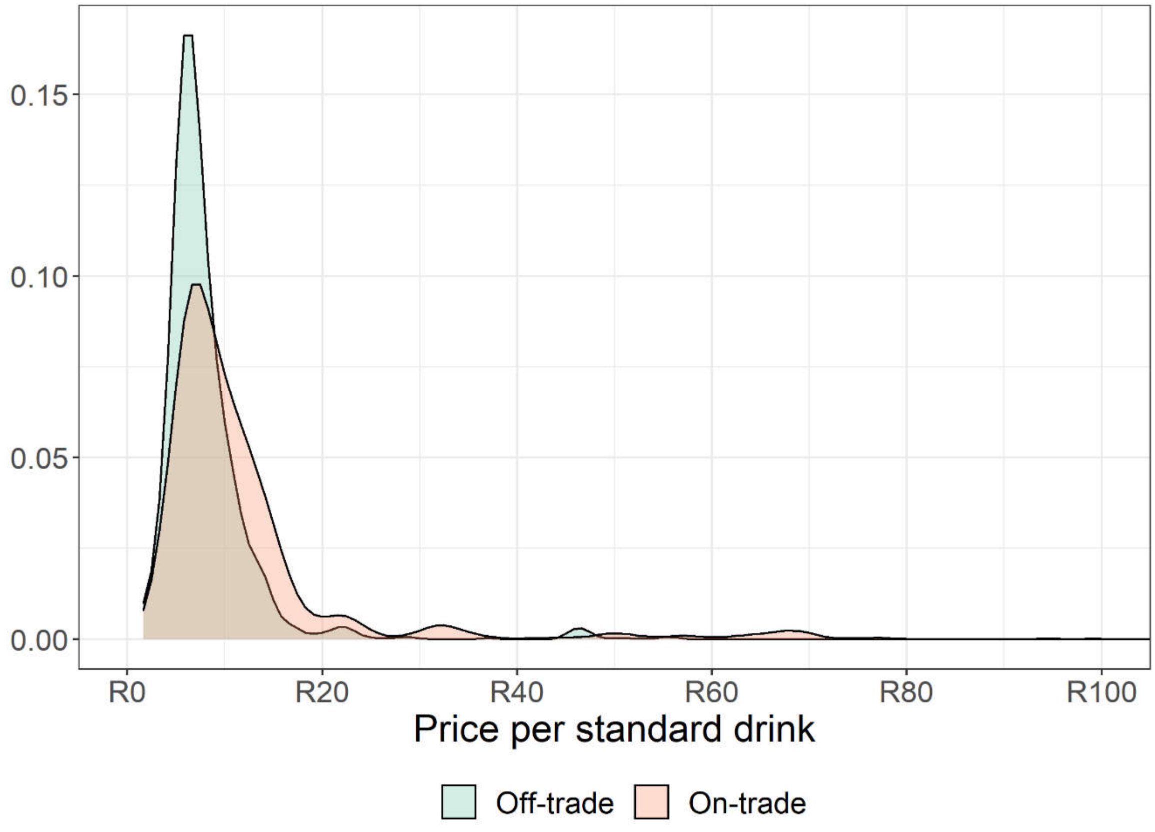

The off-trade wine prices were adjusted using data from the South Africa Wine Industry Statistics [8] who report the proportions of still wine sold (which makes up 93% of total volume of wine sold) in the off-trade in 2018 that falls within different price bands, this data was used to adjust downwards the off-trade wine (Table 6). The price observations were sorted in ascending order and a cumulative volume variable created. The price closest to the 49th percentile was then adjusted down to R3.74 and all prices below adjusted using the same proportion.

The prices at the very bottom were adjusted so they could not go below R2.50. The same adjustment process was applied to each of the four groups.

Table 6: Price distribution for off-trade wine

Retail price per litre of wine for 2018	Price per standard drink (15ml) assuming 12% abv	Cumulative percentage of total still wine sold at price SAWIS data	Cumulative percentage of IAC data for off-trade wine pre-adjustment	Cumulative percentage of IAC data for off-trade wine post-adjustment
**Less than R30**	Less than R3.75	49%	33%	51%
**> R30 – R48**	> R3.75 – R6	82%	60%	83%
**> R48 - R72**	> R6 – R9	89%	77%	89%
**> R72 - 108**	> R9 – R13.5	95%	89%	95%
**> R108**	> R13.5	100%	100%	100%

As the Tschwane prices were collected in one locality, they were validated against national data sources. Beer is by far the most popular drink, accounting for over 50% of the alcohol sold so beer prices are critical. We accessed data from the South Africa Consumer Price Index for January 2020 to compare the Gauteng province (where Tshwane is located) with other provinces. Beer, which accounts for over 50% of alcohol sold in South Africa, Guateng is at R13.76 for a 330ml can. The average across the eight prices listed above is R13.66 which is very close to Guateng's price, therefore we assume the same price distributions across the whole of South Africa.

Finally, prices were validated with all stakeholders including individuals resident in townships who could provide anecdotal evidence relating to cheap alcohol available at shebeens.

For peer review only

6. Base prices by subgroup

All IAC drinkers were now categorised by drinker type and by wealth quintile (Table 7). Wealth quintile was predicted using the ordered choice model created using the SADHS data. Drinkers in the lowest wealth quintile appear the least likely to drink in moderation leaving a very small sample size (this is not weighted by number of drinks). It is therefore not possible to create price distributions for all 15 categories.

Table 7: Count of IAC price observations and respondents within each category

	Moderate obs (individuals)	Occasional Binge obs (individuals)	Heavy obs (individuals)
**Poorest**	2 (2)	29 (23)	35 (24)
**Poorer**	8 (8)	23 (18)	28 (20)
**Middle**	11 (11)	132 (90)	88 (40)
**Richer**	23 (20)	95 (59)	60 (30)
**Richest**	93 (68)	135 (93)	101 (50)

The mean price for each of these drinker categories demonstrates there is wealth gradient (Table 8).

Table 8 Mean price of standard alcoholic drink (15ml of pure alcohol) for each subgroup

	Moderate	Occasional Binge	Heavy
**Poorest**	R6.79	R7.97	R7.78
**Poorer**	R9.43	R10.0	R9.65
**Middle**	R10.2	R10.1	R9.23
**Richer**	R11.3	R13.4	R10.6
**Richest**	R11.7	R11.1	R12.8

In order to ensure adequate sample size the poorest/poorer/middle and richer/richest categories were aggregated for moderate drinkers (Table 9). This represents the final group of prices used in the model.

Table 9 Mean price of standard alcoholic drink (15ml of pure alcohol) within each subgroup

	Moderate	Occasional Binge	Heavy
**Poorest**	R9.13	R7.97	R7.78
**Poorer**	R9.13	R10.0	R9.65
**Middle**	R9.13	R10.1	R9.23
**Richer**	R11.6	R13.4	R10.6
**Richest**	R11.6	R11.1	R12.8

7. Adjusting the elasticities

The starting point for elasticities -0.4, -0.22 and -0.18 for moderate, occasional binge and heavy drinkers respectively [9]. We adjusted these elasticities to incorporate an income gradient using -0.86 and -0.5 elasticity for low and high socioeconomic status [10]. To remain on the conservative side we will count the bottom two quintiles as low SES and the top three as high.

Table 10: Elasticities by wealth and drinker group

Drinker type	Q1	Q2	Q3	Q4	Q5
Moderate	-0.53	-0.53	-0.31	-0.31	-0.31
Occasional binge	-0.29	-0.29	-0.17	-0.17	-0.17
Heavy drinkers	-0.24	-0.24	-0.14	-0.14	-0.14

For peer review only

8. Individual spend, tax and retail revenue

Spend

The total retail spend at baseline, and each scenario, was computed by adding up all the individual spends multiplied by their population weights. This figure was then increased by 1.25 (100/80) to take account for the fact that consumption had only been calibrated to 80% of official sales volume data. This was repeated at R5, R10 and R15.

Government revenue, VAT, excise tax and retail revenue

The following steps were taken in our calculations:

- Calculate VAT by assuming 15% of the base retail spend is VAT
- Import base excise tax from Treasury Budget Report for 2018
- Calculate total volume consumed of alcohol at all four scenarios
- Calculate the percentage change in volume from baseline for each of the three policies
- Apply the percentage change in volume to base excise tax (this assumes a fixed ratio between volume and excise tax)
- Calculate retail revenue by: spend - vat - excise tax

It is likely this is conservative for excise tax revenue as generally the cheaper alcohol, which this policy targets, generates a lower proportion of excise tax than the more expensive so we can consider this a lower band on the excise tax revenue.

For peer review only

Consumption to harm

9. Relative risks

Relative risks were calculated for each of the health outcomes of interest at baseline, and each policy scenario using published relative risk equations [11,12]. The same relative risk equations are used for morbidity (or prevalence) and mortality. HIV risk is derived from a stepped function for mean drinking, intentional injuries and road injury from a continuous function of mean drinking differing by whether the individual binge drinks, liver cirrhosis and breast cancer from a continuous function of mean drinking, for breast cancer this is only for females (Table 10).

Table 11: Relative risk equations used

**Health Condition**	**Relative risk Current drinkers**	**Relative risk former drinkers**	**ICD-10 codes**
**HIV**	Low SES<br>$RR = 2.99$<br>if x > 61/49 grams per day (males/females)<br>$RR = 1.94$ if x > 0<br>$RR = 1$ otherwise<br><br>Higher SES<br>$RR = 1.54$<br>if x > 61/49 grams per day (males/females)<br>$RR = 1$ otherwise	RR = 1	B20-24
**Intentional Injuries**<br><br>**(self-harm and interpersonal violence)**	Drinkers<br>$RR = \exp(00199800266267306 \,.\, x)$<br><br>Heavy episodic drinkers (HED)<br>$RR = \exp(00199800266267306 \,.\, x + 0.647103242058538)$	RR = 1	ICD-10 codes: X60 – Y09<br>Y35 –36<br>Y870<br>Y871
**Road Injury**<br><br>**(pedestrian, cyclist, motorcyclist, motor vehicle, other road)**	Drinkers<br>$RR = \exp(0.00299550897979837 \,.\, x)$<br><br>Heavy episodic drinking<br>$RR = \exp(0.00299550897979837 \,.\, x + 0.959350221334602)$	RR = 1	V01–04,<br>V06,<br>V09–80,<br>V87,<br>V89,<br>V99
**Breast Cancer**	Females only<br>$RR = \exp(0.01018 \,.\, x)$	RR = 1	C50
**Liver**	if x <= 1<br>$1 + x.\exp((\beta_1 + \beta_2)\,.\,\sqrt{\frac{1 + 0.1699981689453125}{100}})$<br><br>If x > 1<br>$\exp((\beta_1 + \beta_2)\,.\,\sqrt{\frac{x + 0.1699981689453125}{100}})$	RR = 3.26 for both females and males	K70, K74

For peer review only

	Female<br>b1 = 2.351821<br>b2 = 0.9002139<br><br>Male<br>b1 = 1.687111<br>b2 = 1.106413		
x = grams of alcohol consumed per day among current drinkers<br>HED = drinking 60 grams or more on one drinking occasion			

For peer review only

10. Potential impact fractions

Potential impact fractions (PIFs) were calculated by dividing relative risk under each policy by relative risk at baseline. These incorporated population weights and were computed by sex *(i)*, wealth group *(j)* and drinker group *(k)*.

$$PIF_{ijk} = \frac{\text{relative risk}_{ijk}\ \text{(policy)}}{\text{relative risk}_{ijk}\ \text{(baseline)}}$$

For peer review only

11. Socioeconomic gradients of ill health

Health outcomes in South Africa are not evenly distributed throughout the population, with the poor often bearing a higher burden of disease, depending on the illness. Data analysis was carried out using General Household Survey (GHS) data for 2018. The ordered choice regression model computed previously, using SADHS data, was applied to the GHS data to split the survey population into wealth quintiles compatible with the foundational dataset (SADHS). Percentage within each wealth quintile with the disease was computed (Table 11). Liver cirrhosis was not one of the health conditions included in the survey and breast cancer was not specifically included although the broader category of cancer was. Sensitivity analysis was carried out using alternative gradients.

Table 12: Raw count of General Household Survey data 2018

	poorest	poorer	middle	richer	richest
**15+ raw count**<br>(648 NAs)	4966	11462	14396	9633	7630
**HIV**<br>raw count<br>percentage	395<br>0.08	684<br>0.06	614<br>0.04	155<br>0.02	41<br>0.005
**Intentional injuries***<br>raw count<br>percentage	11<br>0.002	30<br>0.0027	24<br>0.0018	11<br>0.0012	3<br>0.0002
**Road injuries****<br>raw count<br>percentage	7<br>0.0016	26<br>0.0022	22<br>0.0016	32<br>0.0033	13<br>0.00015
**Cancer**<br>raw count<br>percentage	2<br>0.00038	27<br>0.0012	41<br>0.0026	27<br>0.0029	68<br>0.008

nb: percentages within each quintile were calculated incorporating the survey weights

* gunshot wounds; severe trauma due to violence, assault, beating; intentional poisoning; accidental poisoning; fire and burn; crime related injury – left out sports related, disability related and other
** motor vehicle -occupant, motor vehicle – pedestrian, bicycle related

For peer review only

12. Distributing baseline deaths and cases and calculating probabilities

The deaths/cases (which come disaggregated by sex) at baseline is split between the five wealth quintiles using the GHS data to account for the socioeconomic gradient, as explained above. However, a preparatory step was necessary as the proportions of the population (using the SADHS proportions) in each quintile were not perfectly equal, for example for Q1, Q2, Q3, Q4, Q5 corresponded to 0.19, 0.19, 0.20, 0.21, 0.21 for females and 0.19, 0.20, 0.21, 0.20, 0.21 for males. The probability of death was calculated for each quintile first by assuming the population was split into quintiles of equal size. The total deaths/cases for each quintile using the SADHS proportions was then calculated by applying the relevant probability of death/cases for that part of the quintile which overlapped with the underlying equally sized quintile. This concept can be best illustrated on a graph.

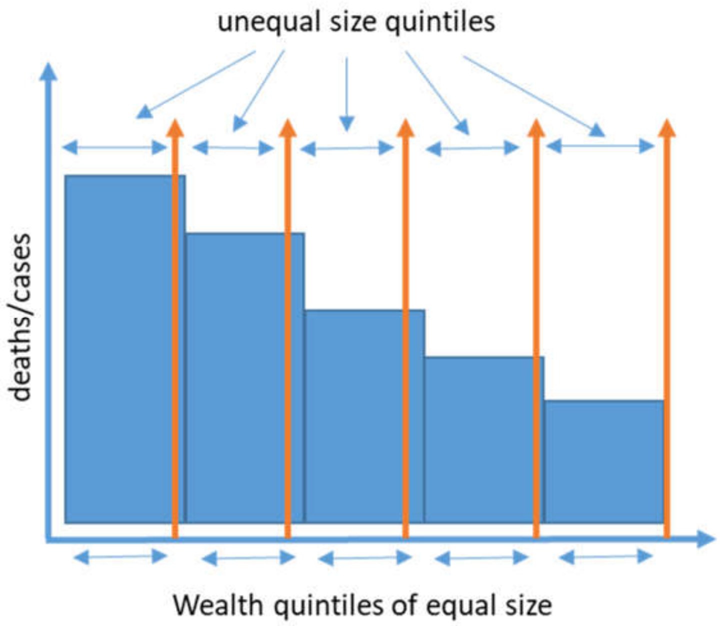

$$Number_{cases}(SADHS_{Q1}) = Pop_{SADHSQ1} \times Prob_{EqualQ1}$$

$$Number_{cases}(SADHS_{Q2}) = (Pop_{EqualQ1} - Pop_{SADHSQ1}) \times Prob_{EqualQ1} + (Pop_{SADHSQ1} + Pop_{SADHSQ2} - Pop_{EqualQ1}) \times Prob_{EqualQ2}$$

$\dots .\, and\ so\ on$

The existence of relative risk equations implies that the baseline mortality/morbidity will also not be distributed equally between drinker groups, one would expect a higher proportion of the baseline cases to exist amongst heavy drinkers, followed by occasional binge, moderate then abstainers. In order for the baseline mortality/morbidity to vary by drinker group the total risk, for each disease, is calculated for each drinker group group, by sex and wealth quintile. The proportional share of risk between drinker groups is then calculated and used to distribute the mortality/morbidity, which has already been assigned to each quintile, between each drinker group within that quintile.

The model uses iHME data for deaths and cases of disease and population statistics (Statistics South Africa) from 2018. Life tables to get the probability of death by single year of age were only available for 2017 from iHME so these were used. The 2018 population is split proportionally into the sex/wealth/drinker groups using the SADHS proportions.

The probability of death for each disease is calculated for the baseline scenario and taken away from overall probability of death for each single year of age given in the life table to give a probability of death from non-modelled causes. This probability of death from non-modelled causes remains constant at every policy scenario. The probability of death from the five diseases of interest then vary according to the policy level and the corresponding potential impact fraction.

We model counterfactual population structure (i.e. in the absence of the policy) over 20 years, starting from 2018 using current population estimates from Statistics South Africa, plus birth projections for 2020 to 2023 and assume current age-, sex- and wealth-specific mortality rates remain constant [13]. Birth cohorts for years beyond 2023 are not modelled as they would not have reached the age at which we model alcohol consumption (15+) within the time horizon.

We create multistate life tables in which the population faces a probability of mortality for each of the five disease/injury conditions and for other cause mortality each year. This approach allows us to simulate prevalence of and mortality from multiple diseases simultaneously, assuming diseases are independent of one another. The model generates alternative population impact fractions (as above) for baseline and for each policy scenario. Using the relevant population impact fraction and rerunning the multistate life table enables a calculation of the difference between baseline and the policy.

For peer review only

13. Baseline health and lagged health impact

HIV, road injuries and intentional injuries realise the full impact of the reduction in drinking immediately whereas the health impact on liver cirrhosis and breast cancer are subject to lags in the effect, meaning the reduced drinking does not translate to a reduced health risk immediately [14]. Breast cancer starts to see an impact at year 11 and it is 20 years until full effect, liver cirrhosis sees some impact from year one but does not realise the full effect until year 20 (Appendix part 9).

The life tables for the 20 year time horizon are saved for each of the policy scenarios. They are then used in combination with the probability of having the disease and the potential impact fraction under each policy, to estimate the number of cases.

HIV, road injuries and intentional injuries realise the full impact of the reduction in drinking from the first year of the drinking reduction whereas liver cirrhosis and breast cancer are subject to lags in the effect. Breast cancer only starts to see an impact at year 11 and it is 20 years until full effect, liver cirrhosis sees some impact from year one but does not realise the full effect until year 20 (Table 12).

For peer review only

Table 13: Modelled time-lags by condition – proportion of overall change in risk experienced in each year following a change in consumption (Holmes et al., 2012)

**Year**	**1**	**2**	**3**	**4**	**5**	**6**	**7**	**8**	**9**	**10**	**11**	**12**	**13**	**14**	**15**	**16**	**17**	**18**	**19**	**20**
**Breast cancer**	0	0	0	0	0	0	0	0	0	0	10	20	30	40	50	60	70	80	90	100
**Liver Cirrhosis**	21	34	43	50	56	61	65	69	73	76	79	82	85	88	90	92	94	96	98	100

14. Hospital multipliers and costs

The prevalence of disease/injury at each policy scenario for each year of the 20 year time horizon was multiplied by the proportion who would then go on to receive hospital treatment (Table 13) and the relevant hospital cost applied (Table 14). The costs taken from the literature were increased by inflation where necessary to reach the baseline year of 2018. Future costs were discounted at 5% as recommended by the Department of Health in the guidelines for pharmacoeconomic submissions [15].

Table 14: Estimated multiplier from population prevalence to hospital admission

Condition	Multiplier (cases in population who go on to receive healthcare treatment)	Source
**HIV**	0.62	UNAIDS estimates that 62% of people living with HIV in 2018 in South Africa were on treatment [16]
**Intentional Injury**	0.41	Survey estimating trauma admissions [17] combined with iHME data from the same year to predict multipliers.
**Road injury**	0.19	Survey estimating trauma admissions [17] combined with iHME data from the same year to predict multipliers.
**Liver Cirrhosis**	0.5	Paper on liver cirrhosis in sub-Saharan Africa suggests 50% of patients are admitted to hospital with end-stage liver disease [18].
**Breast Cancer**	0.75	All studies found estimate what proportion present with late stage breast cancer (51%) but not what proportion never receive hospital treatment [19]. Therefore an estimate of 0.75 is used.

Table 15: Hospital costs and sources

Condition	Cost per patient	Source
**HIV**	R 3,318.62 (2017/18)	This is the annual cost. Taken from a systematic literature review of per patient costs of HIV services in South Africa [20]. There are many different levels of treatment, this cost is only for first-line treatment, so this is conservative.
**Intentional Injury**	R58,928 (2013)	This retrospective case note review included 143 violence related emergency hospital admissions from January to March 2013. Average inpatient stay was 9.8 days with treatments including emergency surgery, intensive care and resuscitation beds on admission [21].
**Road injury**	R56,592.17 (2012)	A prospective cohort study followed 100 patients admitted following a Road traffic injury between late 2011 and early 2012 at Edendale Hospital Pietermartizburg [22].
**Liver Cirrhosis**	R2,967 (2018)	50% multiplier used above comes from paper suggesting 50% of liver cirrhosis patients get admitted to hospital with end stage liver disease. Treatment for end stage liver disease includes<br><br>A specific study on liver cirrhosis was not found so general costs have been used from the district health baromenter. Expenditure per patient day equivalent (district hospitals) was R2967 (average taken from

		across the 9 provinces). This assumes just one patient day. Conservative. [23]
**Breast Cancer**	Early stage R14,915<br>Late stage R16,869<br>(2015)	This retrospective case review included 200 women at a government hospital in South Africa. The average cost is different depending on whether they were diagnosed at an early (56%) or late (44%) stage [24].

For peer review only

For peer review only

15. Sensitivity Analysis

Parameter	Central estimate	Alternative plausible values	Rationale	Results
**Price elasticities**	-0.53 moderate Q1, Q2<br>-0.31 moderate Q3, Q4, Q5<br><br>-0.29 occasional binge Q1, Q2<br>-0.17 occasional binge Q3, Q4, Q5<br><br>-0.24 heavy Q1, Q2<br>-0.14 heavy Q3, Q4, Q5	Scenario 1<br>-0.40 moderate<br>-0.22 occasional binge<br>-0.18 heavy<br><br>Scenario 2<br>-0.5 for high income drinkers (applied to quintiles 3, 4, 5)<br><br>-0.86 for low income drinkers (applied to quintiles 1,2 to be conservative)<br><br>Scenario 3<br>-0.8	Scenario 1<br>Applies estimates based only on drinker type, removing any wealth gradient.<br><br>Scenario 2<br>Estimates using NIDs data for two subsets of the population, the top 50% and bottom 50% of households by total household expenditure [10].<br><br>Scenario 3<br>Van Walbeek and Blecher [10] literature review of South African specific price elasticities found Selvanathan and Selvanathan [25] estimated -0.8 which corresponds closely to price elasticity estimates for beer (-0.8), wine (-0.9) and spirits (-0.9) produced by SALBA (2010).	Central estimates<br>Consumption - 4.40%<br>Spend 18.09 %<br>Lives saved 20,585<br>Cases saved 900,332<br><br>Scenario 1<br>Consumption - 4.50%<br>Spend 17.86%<br>Lives saved 18,717<br>Cases saved 825,935<br><br>Scenario 2<br>Consumption - 14.16%<br>Spend 5.4%<br>Lives saved 52,419<br>Cases saved 2,331,362<br><br>Scenario 3<br>Consumption - 17.96%<br>Spend 0.1 %<br>Lives saved 64,494<br>Cases saved 2,891,284
**Proportion of abstainers in the population**	82% female non-drinkers<br>45% male non-drinkers	67% female non-drinkers<br>36% male non-drinkers	Stakeholders have indicated scepticism about the prevalence of non-drinking	Central estimates<br>Consumption - 4.40%<br>Spend 18.09 %

For peer review only - http://bmjopen.bmj.com/site/about/guidelines.xhtml

			reported in SADHS (and all alcohol studies). Currently the model only adjusts the consumption of those who report anything at all. We will increase the survey weightings of drinkers in the SADHS so that 67% of females do not drink and 36% of males. Based on a South African study which used both surveys and biomarkers [26].	Lives saved 20,585<br>Cases saved 900,332<br><br>Alternative scenario<br>Consumption - 4.48%<br>Spend 17.77%<br>Lives saved 15,616<br>Cases saved 678,929
**HIV baseline estimates**	iHME 2018 estimates<br><br>female<br>77,499 deaths<br>4,772,473 cases<br><br>male<br>70,186 deaths<br>2,799,754 cases	Thembisa 2018 estimates<br><br>female<br>35,487 deaths<br>4,542,677 cases<br><br>male<br>36,345 deaths<br>2,578,747 cases	Stakeholders highlighted the difference between GBD estimates and local estimates for HIV deaths. The Thembisa model was built by local academics and is used by UNAIDs [27].	Central estimates<br>Lives saved 20,858<br>Cases saved 900,332<br>HIV lives saved 10,229<br>HIV cases averted 429,205<br><br>Alternative scenario<br>Lives saved 16,086<br>Cases saved 907,930<br>HIV lives saved 5,486<br>HIV cases averted 423,850
**Socioeconomic gradients of ill health**	HIV<br>Q1 (poorest) – 20%<br>Q2 – 36%<br>Q3 – 32%<br>Q4 – 9%<br>Q5 – 3%<br><br>Intentional Injury/Road Injury/Liver Cirrhosis<br>Q1 – 9%<br>Q2 – 29%<br>Q3 – 26%<br>Q4 – 26%<br>Q5 – 10%	Scenario 1<br>Changing the liver cirrhosis gradient to match the one used for breast cancer	Scenario 1<br>Stakeholders indicated that for long-term conditions like cirrhosis wealthier groups could well be over-represented in SA. They suggested sensitivity analysis by applying values for a condition that is less concentrated amongst the poor.	Central estimates<br>Liver cirrhosis<br>lives saved/cases averted<br>Q1 133 / 3,528<br>Q2 432 / 11,298<br>Q3 295 / 7,801<br>Q4 288 / 7,639<br>Q5 82 / 2,158<br><br>Scenario 1<br>Liver cirrhosis lives saved/cases averted<br>Q1 95 / 2509<br>Q2 104 / 2722

For peer review only

	Breast cancer<br>Q1 – 7%<br>Q2 – 7%<br>Q3 – 22%<br>Q4 – 18%<br>Q5 – 47%	Scenario 2<br>HIV<br>Q1 (poorest) – 25%<br>Q2 – 22%<br>Q3 – 20%<br>Q4 – 18%<br>Q5 – 14%<br><br>Intentional injury/ Road injury/Liver cirrhosis<br>Q1 – 20%<br>Q2 – 20%<br>Q3 – 19%<br>Q4 – 20%<br>Q5 – 22%<br><br>Breast cancer<br>Q1 (poorest) – 21%<br>Q2 – 21%<br>Q3 – 20%<br>Q4 – 19%<br>Q5 – 18%	Scenario 2<br>Recent data from another South African survey is used to provide plausible alternative socioeconomic gradients across all the conditions used in the model [28].	Q3 235 / 6203<br>Q4 200 / 5316<br>Q5 359 / 9563<br><br>Central estimates aggregate lives saved / cases averted<br>Q1 4,088 / 176,663<br>Q2 7,375 / 313,360<br>Q3 4000 / 177,604<br>Q4 3,759 / 167,934<br>Q5 1,364 / 64,771<br><br>Scenario 2 aggregate lives saved / cases averted<br>Q1 2,858 / 127,516<br>Q2 5,246 / 225,067<br>Q3 5,758 / 255,667<br>Q4 3,153 / 139,2253<br>Q5 3,969 / 197,191
**Discount rates for costs**	5% discount rate	Scenario 1<br>0% discount rate	Discount rate was changed to 0%	Central estimate<br>Health costs saved<br>R6.88 billion<br><br>Scenario 1<br>Health costs saved<br>R11.10 billion

For peer review only

**Homebrew switching**	30%	Scenario 1<br>0%<br><br>Scenario 2<br>100%	The assumption that drinkers will make up 30% of the reduction in drinking recorded alcohol with homebrew comes from consultation with the stakeholders at workshop two. To test the importance of this assumption on the results a null impact and a 100% impact are introduced. 100% would mean that any homebrew drinkers will not receive any positive health impacts from the policy as all of their reduction in recorded alcohol will be replaced with homebrew alcohol.	Central estimate<br>Consumption - 4.40%<br>Lives saved 20,585<br>Cases saved 900,332<br><br>Scenario 1<br>Consumption - 4.56%<br>Lives saved 21,479<br>Cases saved 937,507<br><br>Scenario 2<br>Consumption - 4.03 %<br>Lives saved 19,156<br>Cases saved 844,471

For peer review only

16. Healthcare cost savings by quintile

Table 16: Health care costs for each of the three policy scenarios split by wealth quintile

	Q1	Q2	Q3	Q4	Q5
			**R5 MUP**		
**HIV**	-R0.01	-R0.07	-R0.04	-R0.03	-R0.01
**Intentional injury**	R1.41	R5.22	R5.42	R12.8	R7.72
**Road injury**	R0.71	R2.73	R2.80	R6.39	R3.82
**Liver cirrhosis**	R0.02	R0.12	R0.11	R0.27	R0.14
**cancer**	R0.00	R0.00	R0.01	R0.05	R0.15
			**R10 MUP**		
**HIV**	R162.00	R291.00	R71.10	R8.72	R33.3
**Intentional injury**	R495.57	R801.23	R1150.94	R1487.35	R369.03
**Road injury**	R232.98	R399.34	R520.70	R658.80	R163.64
**Liver cirrhosis**	R3.03	R9.64	R6.62	R6.45	R1.86
**cancer**	R0.30	R0.22	R0.80	R0.93	R1.75
			**R15 MUP**		
**HIV**	R403.19	R618.29	R190.50	R79.85	R64.67
**Intentional injury**	R1136.23	R2029.50	R2558.09	R2350.20	R1014.96
**Road injury**	R536.83	R1013.46	R1173.35	R1080.17	R4618.76
**Liver cirrhosis**	R7.42	R23.50	R17.60	R15.20	R4.51
**cancer**	R0.76	R0.65	R2.24	R2.30	R4.65

For peer review only

References

1. Kehoe T, Gmel G, Shield KD, Gmel G, Rehm J. Determining the best population-level alcohol consumption model and its impact on estimates of alcohol-attributable harms. *Population health metrics* 2012; **10**(1): 6.
2. Rehm J, Kehoe T, Gmel G, Stinson F, Grant B, Gmel G. Statistical modeling of volume of alcohol exposure for epidemiological studies of population health: the US example. *Population Health Metrics* 2010; **8**(1): 3.
3. Meier PS, Meng Y, Holmes J, et al. Adjusting for unrecorded consumption in survey and per capita sales data: quantification of impact on gender-and age-specific alcohol-attributable fractions for oral and pharyngeal cancers in Great Britain. *Alcohol and Alcoholism* 2013; **48**(2): 241-9.
4. Probst C, Shuper PA, Rehm J. Coverage of alcohol consumption by national surveys in South Africa. *Addiction* 2017; **112**(4): 705-10.
5. Gmel G, Shield KD, Kehoe-Chan TA, Rehm J. The effects of capping the alcohol consumption distribution and relative risk functions on the estimated number of deaths attributable to alcohol consumption in the European Union in 2004. *BMC medical research methodology* 2013; **13**(1): 24.
6. Brennan A, Meier P, Purshouse R, et al. The Sheffield alcohol policy model–a mathematical description. *Health economics* 2015; **24**(10): 1368-88.
7. Venables WN, Ripley BD. Modern Applied Statistics with S. Fourth Edition. New York: Springer; 2002.
8. SA Wine Industry Information and Systems. SA WINE INDUSTRY 2019 STATISTICS NR 44. South Africa, 2019.
9. Van Walbeek C, Chelwa G. Using price-based interventions to reduce abusive drinking in the Western Cape Province. 2019.
10. Van Walbeek C, Blecher E. The economics of alcohol use, misuse and policy in South Africa. 2014. http://www.tobaccoecon.uct.ac.za/sites/default/files/image_tool/images/405/People/the-economics-of-alcohol-policy-in-south-africa.pdf (accessed 10/03/2019.
11. Shield K, Manthey J, Rylett M, et al. National, regional, and global burdens of disease from 2000 to 2016 attributable to alcohol use: a comparative risk assessment study. *The Lancet Public Health* 2020; **5**(1): e51-e61.
12. Probst C, Parry CD, Rehm J. HIV/AIDS mortality attributable to alcohol use in South Africa: a comparative risk assessment by socioeconomic status. *BMJ open* 2018; **8**(2): e017955.
13. Rebublic of South Africa. Mid-year population estimates, 2019. In: Statistics South Africa, editor.; 2019.
14. Holmes J, Meier PS, Booth A, Guo Y, Brennan A. The temporal relationship between per capita alcohol consumption and harm: a systematic review of time lag specifications in aggregate time series analyses. *Drug and alcohol dependence* 2012; **123**(1-3): 7-14.
15. Republic of South Africa. Guidelines for Pharmacoeconomic Submissions. In: Department of Health, editor.: Government Gazette; 2012.
16. UNAIDS. UNAIDS South Africa: Overview. 2020. https://www.unaids.org/en/regionscountries/countries/southafrica (accessed 27/02/2020.
17. Matzopoulos RG, Prinsloo M, Butchart A, Peden MM, Lombard CJ. Estimating the South African trauma caseload. *International Journal of Injury Control and Safety Promotion* 2006; **13**(1): 49-51.
18. Vento S, Dzudzor B, Cainelli F, Tachi K. Liver cirrhosis in sub-Saharan Africa: neglected, yet important. *The Lancet Global Health* 2018; **6**(10): e1060-e1.
19. Joffe M, Ayeni O, Norris SA, et al. Barriers to early presentation of breast cancer among women in Soweto, South Africa. *PloS one* 2018; **13**(2): e0192071.
20. Meyer-Rath G, van Rensburg C, Chiu C, Leuner R, Jamieson L, Cohen S. The per-patient costs of HIV services in South Africa: Systematic review and application in the South African HIV Investment Case. *PloS one* 2019; **14**(2): e0210497.
21. Bola S, Dash I, Naidoo M, Aldous C. Interpersonal violence: quantifying the burden of injury in a South African trauma centre. *Emergency medicine journal* 2016; **33**(3): 208-12.

For peer review only - http://bmjopen.bmj.com/site/about/guidelines.xhtml

22. Parkinson F, Kent S, Aldous C, Oosthuizen G, Clarke D. The hospital cost of road traffic accidents at a South African regional trauma centre: A micro-costing study. *Injury* 2014; **45**(1): 342-5.
23. Health Systems Trust. District Health Barometer 2018/19, 2020.
24. Guzha N, Thebe T, Butler N, Valodia P. Development of a method to determine the cost of breast cancer treatment with chemotherapy at Groote Schuur Hospital, Cape Town, South Africa. *South African Medical Journal* 2020; **110**(4): 296-301.
25. Selvanathan S, Selvanathan EA. The Demand for Alcohol, Tobacco and Marijuana: International Evidence. Aldershot, England: Ashgate Publishing; 2005.
26. Pisa PT, Vorster HH, Kruger A, Margetts B, Loots DT. Association of alcohol consumption with specific biomarkers: A cross-sectional study in south africa. *Journal of health, population, and nutrition* 2015; **33**(1): 146.
27. Johnson LF, May MT, Dorrington RE, et al. Estimating the impact of antiretroviral treatment on adult mortality trends in South Africa: A mathematical modelling study. *PLoS medicine* 2017; **14**(12): e1002468.
28. Kabudula CW, Houle B, Collinson MA, et al. Socioeconomic differences in mortality in the antiretroviral therapy era in Agincourt, rural South Africa, 2001–13: a population surveillance analysis. *The Lancet Global Health* 2017; **5**(9): e924-e35.

For peer review only

CHEERS Checklist

Section/topic	#	Recommendation	Reported on page #/ line #
**TITLE AND ABSTRACT**			
Title	1	Identify the study as an economic evaluation or use more specific terms such as "cost-effectiveness analysis", and describe the interventions compared.	This is a modelling study, of all the checklists offered this was the most relevant one
Abstract	2	Provide a structured summary of objectives, perspective, setting, methods (including study design and inputs), results (including base case and uncertainty analyses), and conclusions.	page 2 lines 12:17
Abstract			
**INTRODUCTION**			
Background and objectives	3	Provide an explicit statement of the broader context for the study.<br>Present the study question and its relevance for health policy or practice decisions.	Page 4
**METHODS**			
Target population and subgroups	4	Describe characteristics of the base case population and subgroups analysed, including why they were chosen.	Page 5
Setting and location	5	State relevant aspects of the system(s) in which the decision(s) need(s) to be made.	Page 5 Figure 1
Study perspective	6	Describe the perspective of the study and relate this to the costs being evaluated.	Costs were chosen by stakeholders page 5 figure 1
Comparators	7	Describe the interventions or strategies being compared and state why they were chosen.	Page 5 Lines 50 - 55

For peer review only

CHEERS Checklist

Time horizon	8	State the time horizon(s) over which costs and consequences are being evaluated and say why appropriate.	Page 6 lines 40:49
Discount rate	9	Report the choice of discount rate(s) used for costs and outcomes and say why appropriate.	Page 6 lines 57:58
Choice of health outcomes	10	Describe what outcomes were used as the measure(s) of benefit in the evaluation and their relevance for the type of analysis performed.	Page 6 lines 29:38
Measurement of effectiveness	11a	*Single study-based estimates*: Describe fully the design features of the single effectiveness study and why the single study was a sufficient source of clinical effectiveness data	NA
	11b	*Synthesis-based estimates*: Describe fully the methods used for identification of included studies and synthesis of clinical effectiveness data.	NA
Measurement and valuation of preference based outcomes	12	If applicable, describe the population and methods used to elicit preferences for outcomes	NA

Section/topic	#	Checklist item	Reported on page #
Estimating resources and costs	13a	*Single study-based economic evaluation*: Describe approaches used to estimate resource use associated with the alternative interventions. Describe primary or secondary research methods for valuing each resource item in terms of its unit cost. Describe any adjustments made to approximate to opportunity costs.	NA
	13b	*Model-based economic evaluation*: Describe approaches and data sources used to estimate resource use associated with model health states. Describe primary or secondary research methods for valuing each resource item in terms of its unit cost. Describe any adjustments made to approximate to opportunity costs.	Page 6 lines 54-59
Currency, price date, and conversion	14	Report the dates of the estimated resource quantities and unit costs. Describe methods for adjusting estimated unit costs to the year of reported costs if necessary. Describe methods for converting costs into a common currency base and the exchange rate.	Page 6 line 57
Choice of model	15	Describe and give reasons for the specific type of decision-analytical model used. Providing a figure to show model structure is strongly recommended.	Page 5 figure 1
Assumptions	16	Describe all structural or other assumptions underpinning the decision-analytical model.	Page 5 -6
Analytical models	17	Describe all analytical methods supporting the evaluation. This could include methods for dealing with skewed, missing, or censored data; extrapolation methods; methods for pooling data; approaches to validate or make adjustments (such as half cycle corrections) to a model; and methods for handling population heterogeneity and uncertainty.	Appendix parts 1 - 12
**RESULTS**			

For peer review only

CHEERS Checklist

Study parameters	18	Report the values, ranges, references, and, if used, probability distributions for all parameters/ Report reasons or sources for distributions used to represent uncertainty where appropriate. Providing a table to show the input values is strongly recommended.	Page 5 line 60 appendix arts 2,6,9,11
Incremental costs and outcomes	19	For each intervention, report mean values for the main categories of estimated costs and outcomes of interest, as well as mean differences between the comparator groups. If applicable, report incremental cost-effectiveness ratios.	Page 9 table 1,2 Page 10 table 3
Characterising uncertainty	20a	*Single study-based economic evaluation*: Describe the effects of sampling uncertainty for the estimated incremental cost and incremental effectiveness parameters, together with the impact of methodological assumptions (such as discount rate, study perspective).	NA
	20b	*Model-based economic evaluation*: Describe the effects on the results of uncertainty for all input parameters, and uncertainty related to the structure of the model and assumptions.	NA
Characterising heterogeneity	21	If applicable, report differences in costs, outcomes, or cost-effectiveness that can be explained by variations between subgroups of patients with different baseline characteristics or other observed variability in effects that are not reducible by more information.	Page 9/10 Tables 1 - 3 Figures 3&4
**Section/topic**	**#**	**Checklist item**	**Reported on page #**
**DISCUSSION**			
Study findings, limiations, generalisability, and current knowledge	22	Summarise key study findings and describe how they support the conclusions reached. Discuss limitations and the generalisability of the findings and how the findings fit with current knowledge.	Page 11-12
**Other**			
Source of funding	23	Describe how the study was funded and the role of the funder in the identification, design, conduct, and reporting of the analysis. Describe other non-monetary sources of support.	Page 12 lines 30:39
Conflicts of interest	24	Describe any potential for conflict of interest of study contributors in accordance with journal policy. Int eh absence of a journal policy, we recommend authors comply with International Committee of Medical Journal Editors recommendations.	Page 12

For peer review only
